# *cis*‐Carboxylate Enhancement of Mn(III)‐OH Hydrogen Atom Abstraction through PCET Thermodynamics

**DOI:** 10.1002/advs.77944

**Published:** 2026-10-08

**Authors:** Yuri Lee, Yujin Sim, Sehun An, Kyung‐Bin Cho, Jaeheung Cho

**Affiliations:** ^1^ Department of Chemistry Ulsan National Institute of Science and Technology (UNIST) Ulsan Republic of Korea; ^2^ Department of Chemistry and Research Institute of Materials and Energy Sciences Jeonbuk National University Jeonju Republic of Korea

**Keywords:** carboxylate ligand effect, hydrogen atom abstraction, hydrogen bonding, manganese hydroxo complexes, proton‐coupled electron transfer

## Abstract

Manganese(III)‐hydroxo intermediates are proposed as key hydrogen atom abstraction (HAA) species in manganese‐dependent enzymes, yet how first‐sphere carboxylate ligands control their proton‐coupled electron transfer (PCET) thermodynamics remains poorly understood. Here, a *cis*‐(carboxylato)(hydroxo) manganese(III) complex, [Mn^III^(TBDAP)(OH)(C_6_H_5_COO)]^+^ (**1**), is investigated as a functional model of the manganese lipoxygenase active site. Comparison with an alkoxide analogue, [Mn^III^(TBDAP)(OH)(C_6_H_5_CH_2_O)]^+^ (**2**), reveals a 3000‐fold enhancement in HAA reactivity and a large kinetic isotope effect consistent with tunneling‐assisted hydrogen transfer. Redox titration experiments and density functional theory (DFT) calculations support a cooperative thermodynamic contribution involving a positive shift in Mn^III/II^ redox potential and a computationally predicted increase in hydroxo p*K*_a_ associated with intramolecular hydrogen bonding, leading to an increased O–H bond dissociation free energy. *Para*‐substituted benzoate derivatives show only modest changes in HAA rates despite electronic perturbations, consistent with possible partial thermodynamic compensation. The results highlight the role of first‐sphere ligand environments in regulating metal‐hydroxo reactivity through PCET thermodynamics and provide design principles for biological and synthetic oxidation chemistry.

A structurally defined *cis*‐carboxylate dramatically enhances the hydrogen atom abstraction reactivity of a Mn(III)–OH complex. A 3000‐fold rate acceleration, combined with redox measurements and DFT calculations, reveals cooperative control of PCET thermodynamics and provides insight into how first‐sphere carboxylate ligands tune biologically relevant metal–hydroxo intermediates.

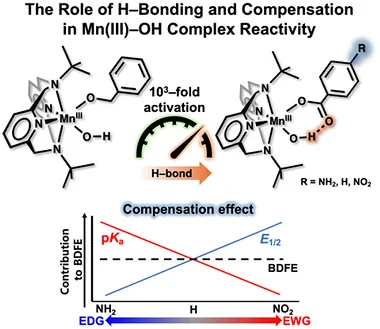

## 1 Introduction

Metalloenzymes play essential roles in a variety of biological catalytic processes [1, 2, 3, 4, 5, 6, 7, 8, 9, 10], often mediating redox transformations and hydrocarbon activation using dioxygen. Over the past few decades, researchers have synthesized a series of model complexes using first‐row transition metals to mimic the active site of metalloenzymes [11, 12, 13, 14, 15]. The synthetic model complexes have provided valuable insights for understanding the mechanisms and reactivities involved in the enzymatic catalytic cycles.

In particular, manganese‐containing enzymes are involved in essential biological transformations such as phosphorylation, decarboxylation, and hydroxylation [1, 3, 16, 17, 18]. For example, manganese superoxide dismutase (MnSOD) catalyzes the disproportionation of superoxide into dioxygen and hydrogen peroxide, protecting cells from oxidative stress [19, 20, 21]. Manganese lipoxygenase (MnLOX), on the other hand, facilitates the oxidation of polyunsaturated fatty acids to generate fatty acid hydroperoxides, initiating biosynthetic pathways for jasmonic acid formation, a key growth‐regulating compound [18]. In MnLOX, the catalytic cycle is proposed to involve a manganese(III)–hydroxo intermediate that initiates hydrogen atom abstraction (HAA) from substrate C─H bonds, followed by subsequent rearrangements leading to oxygenated products [22, 23, 24].

In both MnSOD and MnLOX, the Mn─OH unit has been proposed as a key reactive intermediate. In both enzyme active sites, a carboxylate ligand is positioned *cis* to the hydroxo group, providing structural anchoring as well as electronic modulation of the Mn center. A *cis*‐bound carboxylate is suggested to play a critical role in modulating reactivity and stabilizing transient species [22, 23, 24]. To gain a deeper understanding of the factors governing Mn─OH reactivity, recent efforts have focused on the influence of first‐ and second‐sphere coordination environments surrounding the Mn─OH moiety [25, 26, 27, 28, 29, 30]. For instance, Jackson and co‐workers modulated hydrogen bonding interactions and the electronic properties of Mn(III)−OH complexes to examine HAA behavior [29]. In [Mn^III^(PaPy_2_N)(OH)]^+^ (PaPy_2_NH = *N*,*N*‐bis(2‐pyridinylmethyl)amine‐*N*‐ethyl‐1,8‐naphthyridine‐2‐carboxamide), a nitrogen atom in the adjacent naphthyridine group forms a hydrogen bond with the hydroxy ligand, leading to enhanced HAA reactivity compared to [Mn^III^(PaPy_2_Q)(OH)]^+^ (PaPy_2_QH = *N,N*‐bis(2‐pyridinylmethyl)amine‐*N*‐ethyl‐2‐quinoline‐carboxamide), which lacks such an interaction [30]. However, the specific role of a *cis*‐bound carboxylate ligand, which can simultaneously engage in intramolecular hydrogen bonding and electronic delocalization within the first coordination sphere, remains insufficiently explored. Beyond MnSOD and MnLOX, a closely analogous *cis*‐(carboxylato)(hydroxo) metal(III) moiety has been proposed for nonheme iron oxygenases. In TauD, hydrogen atom transfer from the substrate to a high‐spin Fe(IV)−oxo−succinate species generates an Fe(III)−OH−succinate intermediate, which participates in the catalytic cycle but has so far eluded definitive structural characterization [31, 32]. Therefore, the development of functional model complexes that reproduce the geometric and electronic features of the MnLOX active site is essential for elucidating how carboxylate‐hydroxo interactions govern enzymatic HAA reactivity.

The present study reports the synthesis and characterization of a *cis*‐(carboxylato)(hydroxo)manganese(III) complex, [Mn^III^(TBDAP)(OH)(C_6_H_5_COO)]^+^ (**1**, TBDAP = *N,N’*‐di‐*tert*‐butyl‐2,11‐diaza‐[3.3](2,6)‐pyridinophane), to probe the role of the *cis*‐carboxylate ligand in modulating Mn‐OH reactivity relevant to MnLOX. To elucidate the contributions of hydrogen bonding and resonance stabilization, a *cis*‐(alkoxo)(hydroxo)manganese(III) complex, [Mn^III^(TBDAP)(OH)(C_6_H_5_CH_2_O)]^+^ (**2**), was also prepared. Comparative HAA studies using phenolic substrates reveal that **1** reacts with 4‐methoxy‐2,6‐di‐*tert*‐butylphenol (4‐MeO‐2,6‐DTBP) significantly faster than **2**, highlighting the critical role of the carboxylate ligand. In addition, a series of *para*‐substituted carboxylate complexes, [Mn^III^(TBDAP)(OH)(4‐R−C_6_H_4_COO)]^+^ (R = NH_2_ (**3**) and NO_2_ (**4**)), enables systematic evaluation of electronic effects. Despite substantial electronic perturbations, only modest changes in HAA rates are observed, consistent with a partial compensation between the calculated redox potential and p*K*_a_.

## 2 Results and Discussion

### 2.1 Synthesis and Characterization of **1** and **2**

The precursor complex, [Mn^III^(TBDAP)(OH)_2_]^+^, was prepared according to the literature method [33, 34, 35]. The target *cis*‐(carboxylato)(hydroxo)manganese(III) complex, [Mn^III^(TBDAP)(OH)(C_6_H_5_COO)]^+^ (**1**), was synthesized by the reaction of [Mn^III^(TBDAP)(OH)_2_]^+^ with 2 equiv of benzoic acid under N_2_ in acetonitrile (CH_3_CN) at 25 °C, in which the color of the solution changed from dark green to yellowish green (Scheme 1). The reaction stoichiometry was confirmed through absorption spectral titration (Figure S1). **1** was found to be sufficiently stable at ambient temperature to allow detailed spectroscopic, structural, and reactivity studies.

**SCHEME 1:**
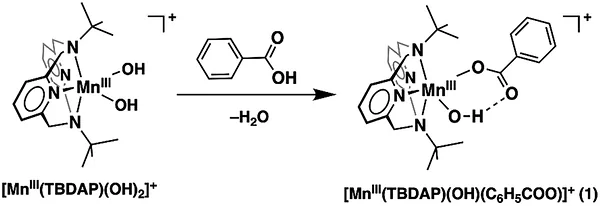
A synthetic procedure for [Mn^III^(TBDAP)(OH)(C_6_H_5_COO)]^+^.

The UV−vis spectrum of **1** in CH_3_CN at 25 °C displays weak absorption bands at 385 (ε = 90 cm^−1^ M^−1^) and 425 nm (ε = 90 cm^−1^ M^−1^) (Figure 1a). The electrospray ionization mass spectrometry (ESI‐MS) spectrum of **1** reveals a positive signal at a mass‐to‐charge ratio (*m/z*) of 545.2, consistent with the calculated mass and isotope distribution pattern of [Mn^III^(TBDAP)(OH)(C_6_H_5_COO)]^+^ (calculated *m/z* 545.2) (Figure 1b). The electron paramagnetic resonance (EPR) spectrum of **1** in a frozen CH_3_CN solution at 100 K is silent, indicating an integer spin state of the Mn center (Figure 1a, inset). The effective magnetic moment (*µ*_eff_) of **1** is determined to be 4.8 *µ*_B_, measured via the ^1^H NMR method of Evans, which indicates a high‐spin state (*S* = 2) of the Mn^III^ ion [36, 37, 38, 39, 40]. The X‐ray crystal structure of **1**‐OTf reveals the mononuclear manganese hydroxide complex with the *cis*‐oriented carboxylate in distorted octahedral geometry (Figure 1c and Tables S1–S3). The length of the Mn─OH bond in **1** (1.799(2) Å) is similar to that found in previously reported manganese(III)‐hydroxo complexes [Mn^III^(PaPy_2_Q)(OH)]^+^ (1.818 Å) and [Mn^III^(PY_5_)(OH)]^2+^ (1.807 Å, PY_5_ = 2,6‐bis(bis(2‐pyridyl)methoxymethane)pyridine) [29, 36]. It is also in good agreement with the DFT‐calculated distance (1.80 Å, vide infra). The Mn─O_benzoate_ bond distance of **1** (1.919(2) Å) is comparable to those observed in [Fe^III^(TBDAP)(OH)(C_6_H_5_COO)]^+^ (1.95−1.96 Å) [41]. The hydrogen atom within the hydroxo group of **1** was successfully refined, providing further confirmation of the Mn^III^−(OH)(C_6_H_5_COO) moiety (Figure S2). Taken together, the spectroscopic and structural analyses confirm the assignment of **1** as a mononuclear *cis*‐(carboxylato)(hydroxo)manganese(III) complex.

**FIGURE 1:**
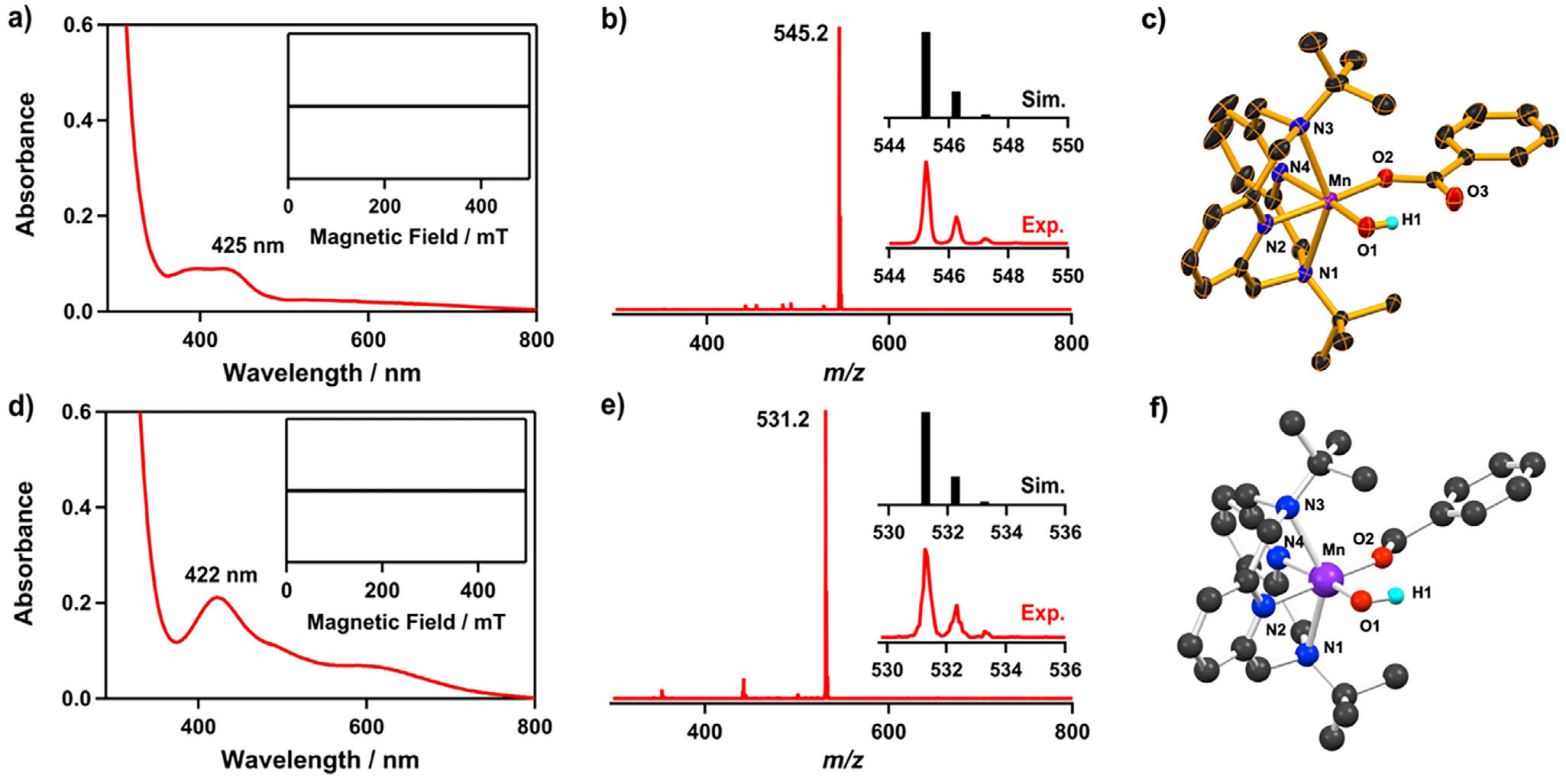
(a) UV−vis spectrum of **1** in CH_3_CN at 25 °C. Inset shows the EPR spectrum of **1** in frozen CH_3_CN at 100 K. (b) ESI‐MS spectrum of **1**. Inset shows the simulated (upper) and experimental (lower) isotope distribution patterns for [Mn^III^(TBDAP)(OH)(C_6_H_5_COO)]^+^. (c) ORTEP diagram of **1** with thermal ellipsoids drawn at the 50% probability (gray, C; cyan, H; blue, N; red, O; violet, Mn). Only the hydrogen atom of the hydroxo group is shown for clarity. (d) UV−vis spectrum of **2** in CH_3_CN at 25 °C. Inset shows the EPR spectrum of **2** in frozen CH_3_CN at 100 K. (e) ESI‐MS spectrum of **2**. Inset shows the simulated (upper) and experimental (lower) isotope distribution patterns for [Mn^III^(TBDAP)(OH)(C_6_H_5_CH_2_O)]^+^. (f) DFT‐optimized structure of **2** (color scheme as in panel (c)).

To probe the roles of hydrogen bonding and resonance, a *cis*‐(alkoxo)(hydroxo) analogue, [Mn^III^(TBDAP)(OH)(C_6_H_5_CH_2_O)]^+^ (**2**), was synthesized by adding an excess amount of benzyl alcohol to [Mn^III^(TBDAP)(OH)_2_]^+^ in CH_3_CN at 60 °C. UV−vis spectrum of **2** shows absorption bands at 422 (*ε* = 220 cm^−1^ M^−1^) and 600 nm (*ε* = 70 cm^−1^ M^−1^) (Figure 1d). **2** is stable at room temperature for several days. ESI‐MS spectrum of **2** exhibits a positive signal at *m/z* 531.2 with a mass and isotope pattern corresponding to [Mn^III^(TBDAP)(OH)(C_6_H_5_CH_2_O)]^+^ (calculated *m/z* 531.3) (Figure 1e). Similar to **1**, **2** is EPR silent at 100 K and its effective magnetic moment (*µ*_eff_ = 5.2 *µ*_B_) supports a high‐spin (*S* = 2) Mn(III) center (Figure 1d, inset). The IR spectrum of **2** exhibits a blue‐shifted O─H stretching vibration relative to **1**, indicating weaker intramolecular hydrogen bonding (Figure S3).

### 2.2 Redox Potentials of **1** and **2**

The one‐electron redox potentials (*E*_red_) of **1** and **2** were determined by absorption spectral titration with decamethylferrocene (Me_10_Fc) in CH_3_CN at 25 °C. Upon stepwise addition of Me_10_Fc to a solution of **1**, an absorption band at 778 nm, assigned to the oxidized decamethylferrocenium (Me_10_Fc^+^) species, was observed (Figure S4) [42]. **1** undergoes equilibrium electron transfer with Me_10_Fc, as evidenced by the increasing concentration of Me_10_Fc^+^ with increasing initial Me_10_Fc concentration (Figure 2 and Equation 1).

(1) $$\begin{array}{ccc} & & {\left[{\text{LMn}}^{\text{III}}\left(\text{OH}\right)\left(C_{6}H_{5}\text{COO}\right)\right]}^{+}\\ & & \;+{\text{Me}}_{\text{10}}\text{eFc}\overset{K_{\text{et}}}{⇌}\left[{\text{LMn}}^{\text{II}}\left(\text{OH}\right)\left(C_{6}H_{5}\text{COO}\right)\right]+{\text{Me}}_{\text{10}}{\text{Fc}}^{+} \end{array}$$

**FIGURE 2:**
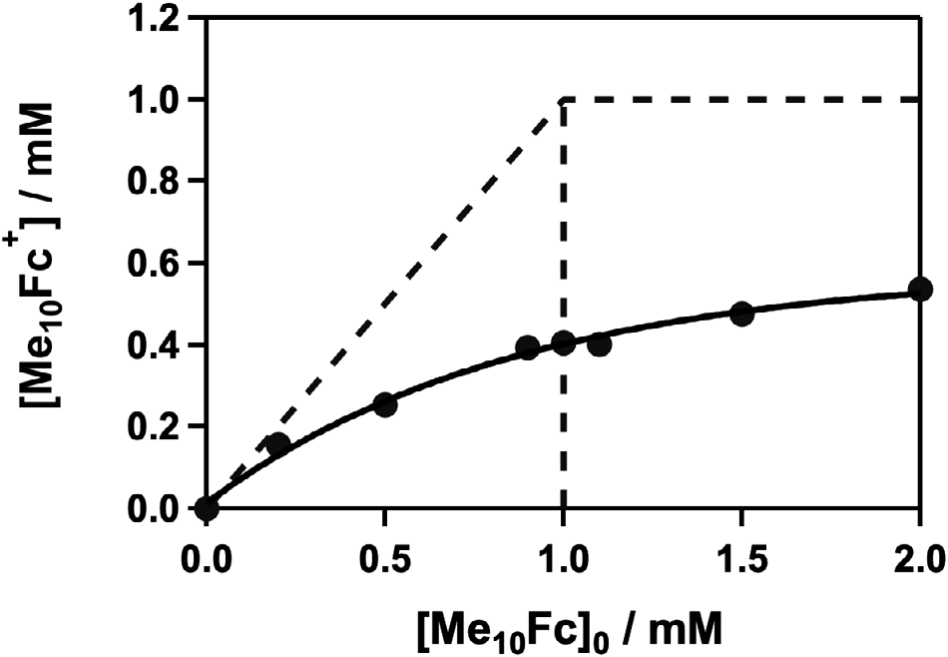
Plot of concentration of Me_10_Fc^+^ produced in electron transfer from Me_10_Fc (−0.096 V vs. SCE) to **1** (1.0 mm) against initial concentration of Me_10_Fc ([Me_10_Fc]_0_) in CH_3_CN at 25 °C. The dotted line represents complete stoichiometric conversion in the absence of equilibrium in the reaction.

The equilibrium constant (*K*_et_) was determined to be 0.43, and the *E*_red_ value of **1** was then calculated as −0.12 V vs. SCE using the Nernst equation (Figures 2 and S5 and Equation 2).

(2) $$E_{\mathrm{red}}=E_{\mathrm{ox}}+\left(\frac{2.3RT}{F}\right)\log K_{et}$$

In contrast, **2** exhibits no evidence of oxidizing Me_10_Fc, even in large excess, whereas **1** readily generates Me_10_Fc^+^ (Figure S6). Furthermore, no spectral changes are observed upon addition of a stronger reductant, chromocene (−0.60 V vs. SCE), indicating that the *E*_red_ value of **2** is more negative than −0.60 V vs. SCE [43]. The results demonstrate that the alkoxo complex **2** is significantly more difficult to reduce than **1**, despite the structural similarity. Among the PCET thermodynamic parameters, the redox properties of **1** and **2** were examined experimentally, with a quantitative *E*_red_ obtained for **1** and a limiting value established for **2**. The p*K*_a_ and BDFE values discussed below (Section 2.4) were derived from DFT calculations because the p*K*_a_ of the Mn─OH_2_ species was not experimentally accessible. All thermodynamic parameters obtained from experiments and calculations are summarized in Table S14.

### 2.3 Reactivity Studies of **1** and **2** with O─H Substrate

To further explore the role of the carboxylate ligand in HAA reactivity, complexes **1** and **2** were examined in O─H bond activation using 4‐MeO‐2,6‐DTBP in CH_3_CN under N_2_. Upon addition of 4‐MeO‐2,6‐DTBP to the solution of **1** at 25 °C, a characteristic absorption band at 407 nm appears, corresponding to the formation of 2,6‐di‐*tert*‐butyl‐4‐methoxyphenoxyl radical (*λ*_max_ = 407 nm, *ε* = 3.1 × 10^3^ M^−1^ cm^−1^ in CH_3_CN) (Figure 3a) [44]. Under excess substrate conditions, the reaction follows pseudo‐first‐order kinetics. A linear correlation between the observed rate constants and 4‐MeO‐2,6‐DTBP concentration yields a second‐order rate constant (*k*_2_) of 3.4(6) M^−1^ s^−1^ (Figure S7). The phenoxyl radical is formed in 90(3)% yield, as determined by UV−vis spectroscopy (Figure 3a) [45]. Product analysis further shows the formation of [Mn^II^(TBDAP)(C_6_H_5_COO)]^+^, confirming that **1** undergoes hydrogen atom abstraction to generate the corresponding Mn(II) species (Figure S8 for ESI‐MS analysis).

**FIGURE 3:**
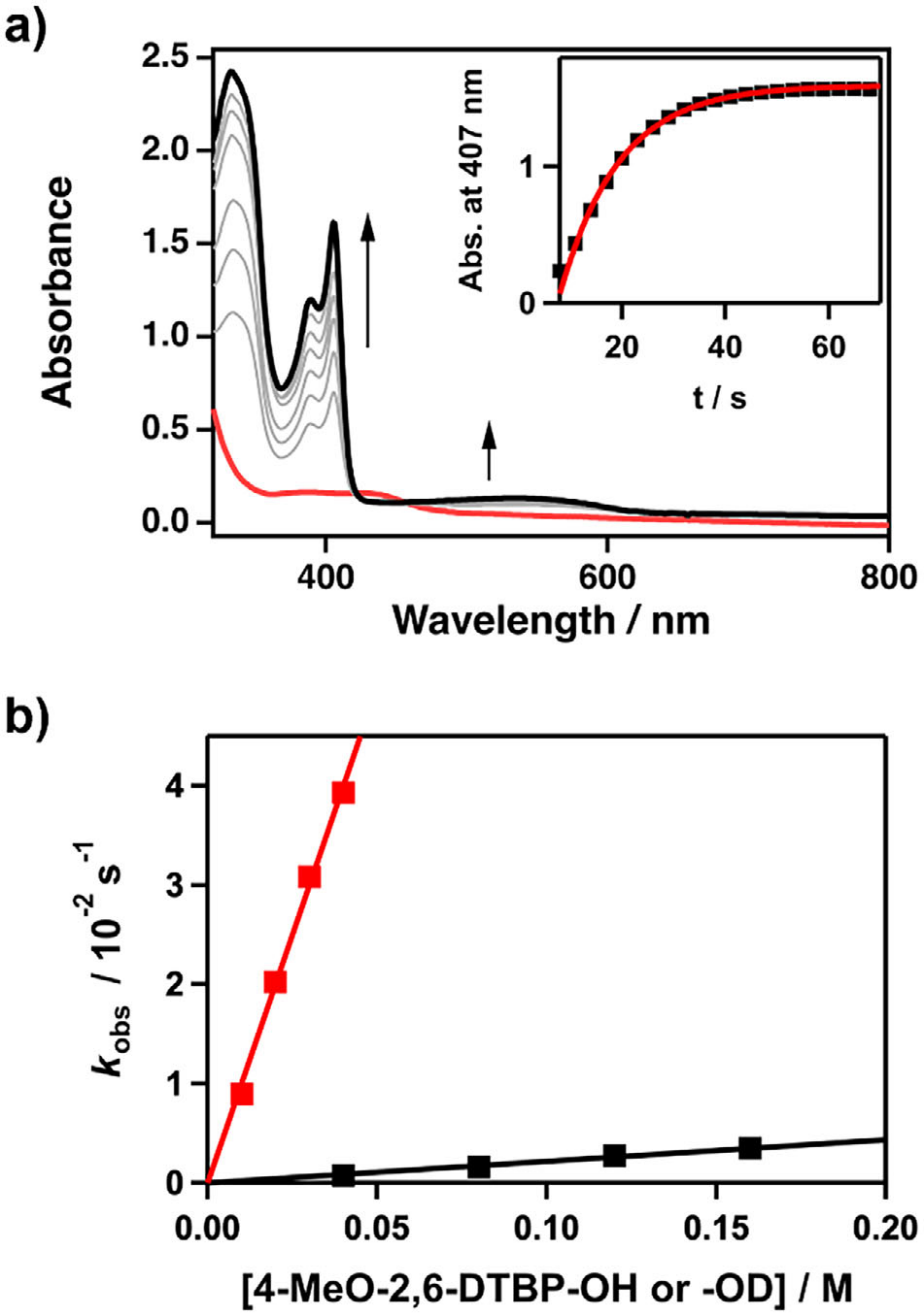
(a) UV−vis spectral changes of **1** (0.5 mM) upon addition of 4‐MeO‐2,6‐DTBP (50 mm) in CH_3_CN at 25 °C. Inset shows the time‐dependent growth of the absorption band at 407 nm. (b) Plots of pseudo‐first‐order rate constants (*k*_obs_) against the concentration of 4‐MeO‐2,6‐DTBP (red squares) and deuterated 4‐MeO‐2,6‐DTBP (black squares) obtained in the presence of excess H_2_O or D_2_O (1.0 M) to determine the kinetic isotope effect (KIE) for **1** (1 mM) at 25 °C.

In contrast, **2** exhibits substantially lower reactivity. While **1** reacted rapidly with 4‐MeO‐2,6‐DTBP at room temperature, **2** showed sluggish reactivity even at a higher temperature and in the presence of excess substrate. Therefore, the reaction of **2** with 4‐MeO‐2,6‐DTBP was investigated at 60 °C in CH_3_CN (Figure S9). Pseudo‐first‐order kinetics were observed, and second‐order analysis gives *k*_2_ value of 1.2(2) × 10^−2^ M^−1^ s^−1^ (Figure S9b). Product analysis confirms the formation of the Mn(II) species, along with the phenoxyl radical in 92(2)% yield (Figure S9a).

The temperature dependence of the reaction rates was examined over the range of 233−263 K for the reaction of **1** with 4‐MeO‐2,6‐DTBP. An Eyring analysis affords activation parameters of Δ*H* ^‡^ = 7.8(3) kcal mol^−1^ and Δ*S* ^‡^ = −29(2) cal mol^−1^ K^−1^ (Figure S10). The observed negative entropy of activation and second‐order kinetics are consistent with a bimolecular associative pathway. Using an Eyring plot, the *k*_2_ value for the O─H bond activation by **1** at 60 °C was determined to be 3.3 × 10^1^ M^−1^ s^−1^. Comparing the *k*_2_ values of **1** and **2** in the reactions with 4‐MeO‐2,6‐DTBP shows a rate acceleration of 3000‐fold for O─H bond activation, highlighting the pronounced effect of the *cis*‐carboxylate ligand on HAA reactivity.

Notably, the kinetic isotope effect (KIE) for **1**, measured using deuterated 4‐MeO‐2,6‐DTBP, is 46 at 25 °C (Figure 3b) [46]. The value significantly exceeds the semiclassical limit commonly associated with a purely over‐the‐barrier transfer, strongly suggesting the involvement of quantum tunneling in the rate‐determining HAA step [47]. The enhanced HAA reactivity of **1** is consistent with a contribution from cooperative PCET thermodynamics involving two factors: i) an experimentally established positive shift in redox potential for **1** relative to **2**, attributed in part to π‐delocalization of the carboxylate ligand, and ii) a computationally predicted increase in p*K*_a_ associated with hydrogen bonding between the hydroxo ligand and the *cis*‐carboxylate. Taken together, these factors are calculated to increase the O─H bond dissociation free energy (BDFE) of the Mn^II^‐OH_2_ species, thereby accelerating HAA (Scheme 2) [48].

**SCHEME 2:**
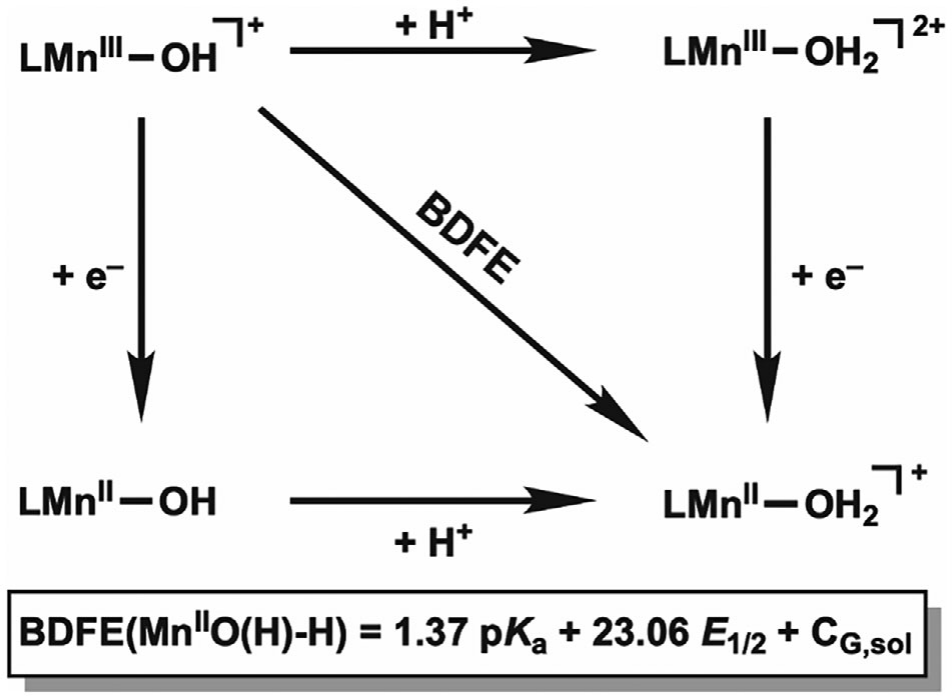
Thermodynamic square scheme for the O─H BDFE of the Mn^III^OH/Mn^II^OH_2_ couple.

### 2.4 DFT Calculations of **1** and **2**

To gain deeper insight into the structural and electronic differences between **1** and **2**, density functional theory (DFT) calculations were performed. Analysis of electronic energies across accessible spin states reveals that the high‐spin quintet (*S* = 2) configuration is the most stable for both **1** and **2** (Table S4), aligning with experimental data and confirming the assignment of the ground spin state of the Mn(III) centers.

The DFT‐optimized structure of **1** closely matches the experimental crystal structure, supporting the reliability of the computational approach (Table S5). The calculated Mn─OH bond length (1.80 Å) is nearly identical to the experimental value (1.799(2) Å), and the Mn─O_benzoate_ bond (1.93 Å) also agrees well with the crystallographic distance (1.919(2) Å). In the case of **2**, which contains an alkoxide ligand instead of a benzoate, the Mn─OH and Mn─O_alkoxide_ bond lengths were calculated to be 1.84 and 1.85 Å, respectively. The Mn─O_benzoate_ bond in **1** was notably longer (1.93 Å) than the Mn─O_alkoxide_ bond in **2** (1.85 Å), which can be attributed to resonance delocalization within the benzoate ligand. The delocalization reduces the negative charge density on the coordinating oxygen, thereby weakening its interaction with the Mn(III) center.

The reaction mechanism of **1** with 4‐MeO‐2,6‐DTBP was further examined. The calculated activation barrier for hydrogen atom abstraction by **1** was 15.3 kcal mol^−1^ (Figure S11 and Tables S6–S8). Analysis of the Mulliken spin density distribution at the transition state (Table S7) reveals that while the structure has its transferring hydrogen in the middle between the oxygens (1.20 Å on each side), the electron transfer is lagging, with only 0.3 *β*‐spin developed so far on the substrate. These findings support a proton‐coupled electron transfer (PCET) pathway, with a proton being transferred first. The reaction ultimately yielded a Mn(II)–aqua complex and a 2,6‐di‐*tert*‐butyl‐4‐methoxyphenoxyl radical, consistent with the experimental detection of radical species by UV–vis spectroscopy.

In order to evaluate the electronic effect of the benzoate ligand, a thermodynamic analysis was performed based on the calculated energies of the three redox/protonation states defined in Scheme 2 (Table S9 and Figures S12 and S13). The Mn(III)–OH (**1** or **2**) and Mn(III)–OH_2_ species represent the deprotonated and protonated forms of the oxidized state, respectively, while Mn(II)–OH and Mn(II)–OH_2_ correspond to the one‐electron reduced counterparts. By comparing the calculated electronic energies between Mn─OH/Mn─OH_2_ species, the redox potential of the Mn(III)/Mn(II) couple of the Mn(III)–OH species and the p*K*_a_ of the Mn(III)–OH_2_ species could be estimated, providing insight into the thermodynamic factors that influence the BDFEs (Figures S12 and S13). The calculated potentials are referenced to SHE, whereas the experimental values in Section 2.2 are referenced to SCE. Given the inherent uncertainty associated with the computational methodology, the calculated p*K*_a_ values, redox potentials, and BDFE values are used primarily to evaluate relative trends within the series rather than as quantitatively accurate absolute values. The calculations show that ligand substitution affects the redox potential more strongly than the acidity. The energy difference between Mn(III)–OH and Mn(III)–OH_2_, which reflects the acidity of the oxidized species and was used to estimate the p*K*_a_, showed only a small variation: −269.4 kcal mol^−1^ for **1** and −272.7 kcal mol^−1^ for **2** (Table S9). In contrast, the energy difference for the Mn(III)–OH to Mn(II)–OH reduction step, which was used to estimate the redox potential, was calculated to be −85.6 kcal mol^−1^ for **1** and −79.4 kcal mol^−1^ for **2**, consistent with the experimentally observed higher redox potential of **1**. A similar redox process of Mn(III)–OH_2_ to Mn(II)–OH_2_ also showed a larger energy difference for **1**‐OH_2_ (−119.4 kcal mol^−1^) than for **2**‐OH_2_ (−109.4 kcal mol^−1^). The large differences in calculated O−H BDFE for **1^red^**‐OH_2_ and **2^red^**‐OH_2_ (65.5 and 56.8 kcal/mol, respectively) correlate well with the reactivity trend (Table S10).

These results suggest that the benzoate ligand primarily modulates the redox potential rather than the proton affinity of the Mn(III)–OH unit. Overall, the DFT results not only support the spectroscopic and electrochemical findings but also provide a mechanistic rationale for the ligand‐dependent modulation of redox properties. Together with experimental data, these findings highlight how ligand electronics govern the reactivity of Mn(III)–OH species, offering guiding principles for designing complexes capable of efficient O─H bond activation. Differences in local geometry, steric accessibility of the hydroxo group, substrate orientation, and reorganization energy may also contribute to the kinetic difference between **1** and **2**. The available data therefore identify PCET thermodynamics as an important contributor to the enhanced HAA reactivity of **1** but do not establish PCET thermodynamics as the sole origin of the rate enhancement.

### 2.5 Synthesis and Characterization of **3** and **4**

To probe how the electronic properties of carboxylate influence Mn(III)‐OH reactivity, two *para*‐substituted benzoate derivatives bearing electron‐donating NH_2_ and electron‐withdrawing NO_2_ groups were incorporated to form [Mn^III^(TBDAP)(OH)(NH_2_−C_6_H_4_COO)]^+^ (**3**) and [Mn^III^(TBDAP)(OH)(NO_2_−C_6_H_4_COO)]^+^ (**4**), respectively. Both complexes were prepared using the same procedures as for **1**, by treating [Mn^III^(TBDAP)(OH)_2_]^+^ in CH_3_CN with 2 equiv of NH_2_−C_6_H_4_COOH or NO_2_−C_6_H_4_COOH. The UV−vis spectra of **3** and **4** closely resembled that of **1** (Figure S14a), indicating similar coordination environments at the metal center. The ESI‐MS spectrum of **3** exhibited a prominent ion peak at *m/z* 560.2, matching the calculated value for [Mn^III^(TBDAP)(OH)(NH_2_−C_6_H_4_COO)]^+^ (calculated *m/z* 560.2) (Figure S14b). Similarly, the ESI‐MS spectrum of **4** showed a major peak at *m/z* 590.2 (calculated *m/z* 590.2), consistent with [Mn^III^(TBDAP)(OH)(NO_2_−C_6_H_4_COO)]^+^ (Figure S14b). Both complexes were EPR silent at 100 K (Figure S155). Single crystal X‐ray diffraction of **3**‐OTf·CH_3_CN and **4**‐OTf confirmed distorted octahedral geometries similar to **1**, with the hydroxo and carboxylate ligands in *cis* positions (Figure 4 and Tables S1–S3). The Mn─OH bond lengths of 1.810(2) Å for **3** and 1.804(2) Å for **4** lie well within the typical range for Mn(III)−OH complexes (1.806−1.854 Å) and are comparable to **1** (1.799(2) Å). The intramolecular H1···O3 contacts of 1.95(5) Å for **3** and 2.03(5) Å for **4** indicate preservation of the first‐sphere hydrogen bonding interaction regardless of substituent electronics [49]. Other bond lengths and angles in **3** and **4** were essentially unchanged relative to **1**, confirming structural consistency across the series.

**FIGURE 4:**
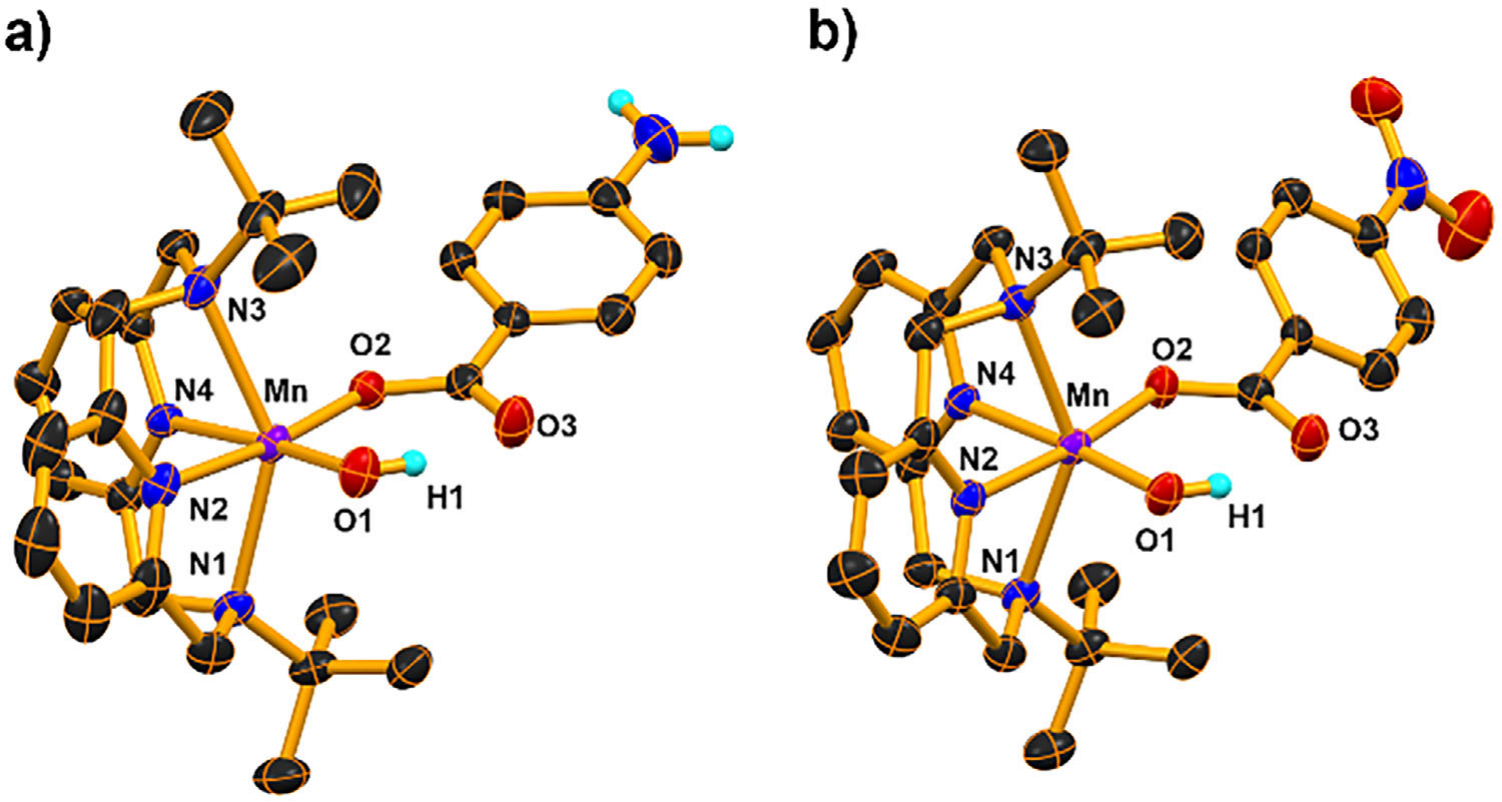
ORTEP diagrams of (a) **3** and (b) **4** with thermal ellipsoids drawn at the 50% probability level (gray, C; cyan, H; blue, N; red, O; violet, Mn). Only hydrogen atoms on the hydroxo and NH_2_ groups are shown for clarity.

### 2.6 Reactivity Studies of **1**, **3**, and **4** with 2,4‐di‐*tert*‐Butylphenol

To evaluate the impact of electronic tuning on HAA reactivity, **1**, **3**, and **4** were reacted with 2,4‐di‐*tert*‐butylphenol (2,4‐DTBP). The addition of 2,4‐DTBP to a CH_3_CN solution of **1** at 25 °C afforded the disappearance of the absorption band at 430 nm due to consumption of **1**, accompanied by the emergence of a new band at 355 nm with an isosbestic point at 382 nm (Figure 5a). The product absorbing at 355 nm was confirmed by ESI‐MS to be a Mn(II) species after HAA (Figure S16b). Analysis of the organic products by ^1^H NMR revealed the formation of 3,3’,5,5’‐tetra‐*tert*‐butylbiphenyl‐2,2’‐diol in 82(12)% yield, confirming phenol dimerization via phenoxy radicals (Figure S17) [46, 50]. Other Mn(III)−OH complexes, **3** and **4**, also react with 2,4‐DTBP in CH_3_CN at 25 °C, exhibiting similar UV−vis spectral changes (Figure S18a), and ESI‐MS spectra confirmed Mn(II) products in each case (Figure S18b). The phenol dimer was formed in yields of 79(13)% for **3** and 88(8)% for **4**, as determined by ^1^H NMR spectroscopy.

**FIGURE 5:**
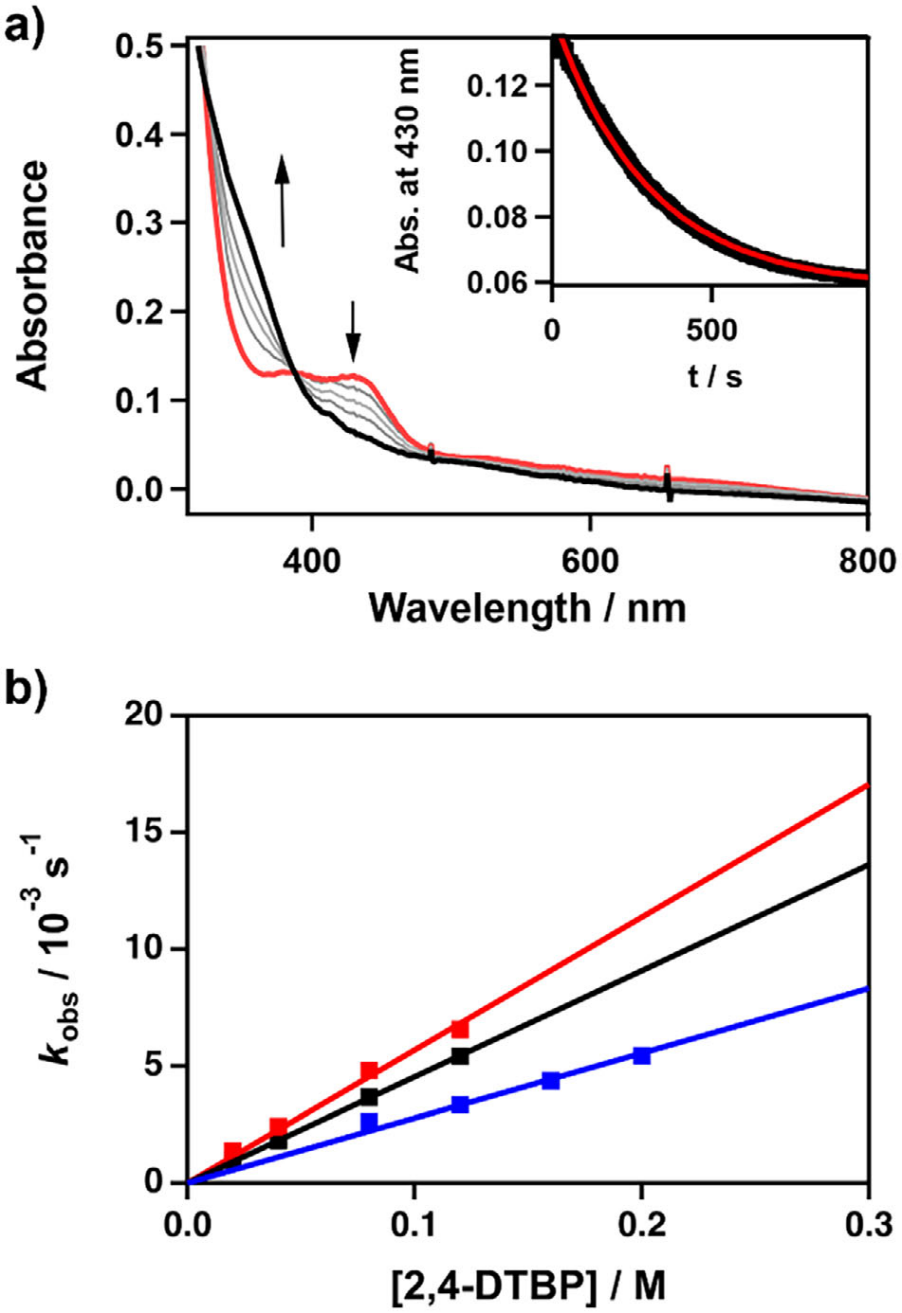
(a) UV−vis spectral changes of **1** (1 mm) upon addition of 2,4‐DTBP (150 mm) in CH_3_CN at 25 °C. The inset shows the time course of the absorption band at 430 nm. (b) Plots of pseudo‐first‐order rate constants (*k*_obs_) against the concentration of 2,4‐DTBP to determine second‐order rate constants for **1** (black), **3** (blue), and **4** (red).

Kinetic analysis showed that all three complexes followed pseudo‐first‐order behavior in the presence of excess 2,4‐DTBP. The *k*_2_ value for the reaction between **1** and 2,4‐DTBP was determined as 4.5(10) × 10^−2^ M^−1^ s^−1^. Reaction rates of **3** and **4** are similar to that of **1**, which were determined to be *k*_2_ = 2.8(5) × 10^−2^ M^−1^ s^−1^ for **3** and *k*_2_ = 5.5(2) × 10^−2^ M^−1^ s^−1^ for **4** (Figure 5b). Despite the significant electronic differences between the substituents, the HAA reactivity remained within a narrow range across the series. The limited variation is consistent with a partial compensation effect in which shifts in redox potential are largely counterbalanced by opposite changes in p*K*_a_, so that the net impact on the overall BDFE is small.

In principle, an electron‐donating substituent (e.g., −NH_2_ in **3**) should strengthen the hydrogen bond and increase the p*K*_a_ of the hydroxo ligand, thereby stabilizing the conjugate acid form and promoting HAA. In contrast, an electron‐withdrawing group (e.g., −NO_2_ in **4**) would be expected to lower the p*K*_a_ and weaken the hydrogen bond. However, such electronic tuning simultaneously affects the Mn(III/II) redox couple inversely: increasing p*K*_a_ often comes at the cost of lowering *E*_red_, and vice versa. As a result, the BDFEs calculated from the DFT‐derived p*K*_a_ and *E*_red_ values using the Bordwell–Mayer relationship remain nearly unchanged across the series (Scheme 2). The results are reminiscent of previously reported compensation phenomena in other metal‐hydroxo systems. For example, Tolman and co‐workers demonstrated that ligand modifications in Cu(III)‐OH complexes produced offsetting changes in redox potential and acidity [51]. A similar phenomenon has also been observed in manganese(V) nitride systems with varying cations [52]. The similar HAA rates of **1**, **3**, and **4** therefore suggest that opposing changes in PCET thermodynamic parameters may partly offset the effect of ligand electronic perturbation within the present series.

### 2.7 DFT Calculations of **1**, **3**, and **4**

A thermodynamic analysis was carried out to further investigate the possible origin of the limited variation in HAA reactivity of **1**, **3**, and **4**. As in the case of **1**, the DFT‐optimized structures of **3** and **4** exhibited good agreement with their crystal structures, particularly regarding the Mn─O bond metrics. The Mn─OH bond lengths were calculated as 1.80 Å in both complexes, closely matching the experimental values of 1.810(2) Å for **3** and 1.804(2) Å for **4**. The Mn─O_benzoate_ distances also aligned well, with DFT values of 1.92 Å for **3** and 1.94 Å for **4**, compared to the crystallographic values of 1.923(2) and 1.936(3) Å, respectively. Moreover, both **3** and **4** were calculated to have a high‐spin quintet (*S* = 2) ground state (Table S4).

To evaluate how *para*‐substitution of the benzoate ligand influences the thermodynamic driving forces for HAA, electronic energy calculations were conducted for **3** and **4**, following the same methodology used for **1** and **2** (Figures S19 and S20). The calculated electronic energy changes for proton transfer and one‐electron reduction were used to estimate the relative p*K*_a_ values and redox potentials, respectively. The p*K*_a_ values, derived from the energy difference between Mn(III)–OH and Mn(III)–OH_2_, followed the trend of **3**‐OH_2_ (−3.19) > **1**‐OH_2_ (−6.19) > **4**‐OH_2_ (−8.07), indicating that **4**‐OH_2_ is the most acidic (Table S10). The most negative energy value observed for **3**‐OH_2_ can be attributed to the electron‐donating NH_2_ group, which stabilizes the protonated Mn(III)–OH_2_ species through strengthening hydrogen bonding. Conversely, the electron‐withdrawing NO_2_ group in **4**‐OH_2_ weakens hydrogen bonding, resulting in a lower p*K*_a_. In contrast, the redox potentials, estimated from the energy differences between Mn(III)–OH and Mn(II)–OH, exhibited the opposite trend compared to the p*K*_a_ values: **4** (−0.31 vs. SHE) > **1** (−0.37 vs. SHE) > **3** (−0.46 vs. SHE) (Table S10). The strongly electron‐donating NH_2_ group in **3** stabilizes the oxidized Mn(III) center, resulting in a lower redox potential, while the electron‐withdrawing NO_2_ group in **4** destabilizes the Mn(III) oxidation state, shifting the Mn(III/II) couple to a more positive potential. Although the HAA reaction proceeds through a stepwise PCET pathway in which electron transfer occurs from the protonated Mn(III)–OH_2_ species, the corresponding Mn(III)–OH_2_ to Mn(II)–OH_2_ redox process shows a consistent energetic trend. The calculated electronic energy differences for **1**‐OH_2_, **3**‐OH_2_, and **4**‐OH_2_ are −119.4 kcal mol^−1^, −113.7 kcal mol^−1^, and −121.7 kcal mol^−1^, respectively, converting to redox potentials of 1.07 V vs. SHE, 0.86 V vs. SHE, and 1.17 V vs. SHE (Tables S9 and S10). The opposing calculated trends in p*K*_a_ and redox potential are consistent with a partial thermodynamic compensation effect, yielding nearly constant O–H BDFEs across the series (Table S10) [53, 54]. The differences among the calculated O−H BDFEs (∼1 kcal/mol) for **1^red^**‐OH_2_, **3^red^**‐OH_2_, and **4^red^**‐OH_2_ are well within the typical error limits of standard DFT calculations. The computed compensation behavior is consistent with the experimentally observed similarity in HAA rates for **1**, **3**, and **4**, despite substantial differences in the *para*‐substituted benzoate ligands.

DFT calculations were additionally performed to examine the reactions of **1**, **3**, and **4** with 2,4‐DTBP (Tables S11–S13). For **1**, the activation barrier for HAA was calculated as 16.8 kcal mol^−1^, and the reaction proceeds via a stepwise PCET mechanism, in which proton transfer is followed by electron transfer (Figure S21). Unlike the reaction with 4‐MeO‐2,6‐DTBP, which is exothermic and results in organic radical species, the HAA process with 2,4‐DTBP is endothermic for all species (Table S11) and is followed by rapid dimerization to form 3,3’,5,5’‐tetra‐*tert*‐butylbiphenyl‐2,2’‐diol. This aligns with the UV–vis data, where distinct radical features were not observed for the 2,4‐DTBP reactions. Complexes **3** and **4** displayed the same stepwise PCET mechanism, with slightly different activation barriers of 17.1 and 16.5 kcal mol^−1^, respectively (Figures S22 and S23). The calculated activation barriers followed the trend of **4** (16.5 kcal mol^−1^) < **1** (16.8 kcal mol^−1^) < **3** (17.1 kcal mol^−1^), which was consistent with the experimentally determined second‐order rate constants: **4** (5.5(2) × 10^−2^ M^−1^ s^−1^) > **1** (4.5(10) × 10^−2^ M^−1^ s^−1^) > **3** (2.8(5) × 10^−2^ M^−1^ s^−1^). Although the calculated barriers by only 0.6 kcal mol^−1^, within typical computational uncertainties, their ordering is in agreement with the experimentally observed reactivity. These findings support that the *para*‐substituent electronics modulate acidity and redox potential in opposing directions, leading to convergence on similar overall reaction barriers. The similar experimental HAA rates and calculated PCET thermodynamic parameters are consistent with partial thermodynamic compensation within the **1**, **3**, and **4** series. Given the narrow range of experimental rate constants and the limited number of complexes examined, thermodynamic compensation is proposed as a plausible interpretation rather than a general substituent relationship. Evaluation of a larger substituent series will be required to test the broader application of the proposed relationship.

## 3 Conclusion

The study presents a systematic investigation of how a *cis*‐carboxylate ligand modulates the reactivity of Mn(III)‐OH complexes, as functional models of the MnLOX active site. The carboxylate‐bound complex, [Mn^III^(TBDAP)(OH)(C_6_H_5_COO)]^+^ (**1**), and its alkoxide analogue, [Mn^III^(TBDAP)(OH)(C_6_H_5_CH_2_O)]^+^ (**2**), enable direct evaluation of hydrogen bonding and resonance effects, while *para*‐substituted carboxylate derivatives, [Mn^III^(TBDAP)(OH)(4‐R−C_6_H_4_COO)]^+^ (R = NH_2_ (**3**), NO_2_ (**4**)) allow assessment of electronic tuning within a conserved coordination environment. **1**–**4** were characterized using various analytical methods, such as UV−vis, ESI‐MS, EPR, ^1^H NMR, and x‐ray crystallography.

Kinetic and thermodynamic analyses reveal that **1** exhibits dramatically enhanced hydrogen atom abstraction (HAA) reactivity compared to **2**. The enhanced HAA reactivity of **1** is consistent with a substantial contribution from cooperative PCET thermodynamics, involving an experimentally established positive shift in redox potential and a computationally predicted increase in BDFE associated with the *cis*‐carboxylate ligand, although additional structural and kinetic factors cannot be excluded. A large kinetic isotope effect (KIE) and negative activation entropy support a bimolecular H‐atom transfer pathway with significant quantum tunneling contributions. In contrast, electronic perturbation in **3** and **4** produced only modest changes in HAA reactivity, consistent with partial compensation in which the calculated redox potential and p*K*_a_ shift in opposing directions, resulting in small changes in BDFE. Overall, the study demonstrates that first‐sphere hydrogen bonding and ligand electronic properties cooperatively govern Mn─OH reactivity by tuning PCET thermodynamics. These findings provide a design principle for tuning PCET thermodynamics in transition metal hydroxo intermediates relevant to biological oxidation and synthetic energy conversion, including Mn(III)‐OH species in manganese enzymes and the proposed Fe(III)‐OH‐succinate intermediates of nonheme iron oxygenases.

## Author Contributions

**Yuri Lee**: methodology, conceptualization, investigation, funding acquisition, writing – original draft. **Yujin Sim**: conceptualization, methodology, investigation. **Sehun An**: methodology, investigation, formal analysis, visualization, writing – original draft. **Jaeheung Cho**: conceptualization, validation, supervision, funding acquisition, writing – review and editing. **Kyung‐Bin Cho**: investigation, validation, supervision, funding acquisition, writing – review and editing.

## Funding

National Research Foundation funded by the Ministry of Science, ICT and Future Planning (RS‐2024‐00333606 & RS‐2026‐25528325 to J.C. and RS‐2024‐00451074 to Y.L.) of Korea. InnoCORE program of the Ministry of Science and ICT (UNIST 1.260040.01) to J.C., RS‐2021‐NR058689 to K.‐B.C., and MoE G‐LAMP Program RS‐2024‐00443714 to K.‐B.C.

## Conflicts of Interest

The authors declare no conflicts of interest.

## Supporting information




**Supporting File**: advs77944‐sup‐0001‐SuppMat.docx.

## Data Availability

The data that support the findings of this study are available in the supplementary material of this article.
