# Supplementary material for: cis‐Carboxylate Enhancement of Mn(III)‐OH Hydrogen Atom Abstraction through PCET Thermodynamics
Source: Adv Sci (Weinh). 2026 Oct 8:e77944. Online ahead of print. doi: 10.1002/advs.77944 (PMC13650034; doi:10.1002/advs.77944)
Supplement: Supplementary file 1 [file ADVS-9999-e77944-s001.docx]

*cis*-Carboxylate Enhancement of Mn(III)-OH Hydrogen Atom Abstraction through PCET Thermodynamics

Yuri Lee,^†^ Yujin Sim,^†^ Sehun An,^†^ Kyung-Bin Cho,* and Jaeheung Cho*

^†^These authors contributed equally

*Corresponding authors: workforkyung@jbnu.ac.kr, jaeheung@unist.ac.kr

**Experimental Section**

**Materials.** All chemicals purchased from Aldrich Chemical Co. or Alfa Aesar Co. were used without additional purification. CH_3_CN and Tetrahydrofuran ((CH_2_)_4_O) were purified through solvent purification columns (JC Meyer Solvent Systems) before use. TBDAP ligand was prepared by reacting 2,6-bis-(chloromethyl)pyridine with 2,6-bis[(N-*tert*-butylamino)methyl]-pyridine at 80 ºC, as previously described in the literature.^S1^ [Mn^II^(TBDAP)(OTf)_2_] was prepared following established literature protocols.^S2^

**Instrumentation.** UV−vis spectra were obtained using a Hewlett Packard 8454 diode array spectrophotometer, which was equipped with a UNISOKU Scientific Instruments for low-temperature experiments or a circulating water bath for temperature control. ESI-MS spectra were collected on a Waters (Milford, MA, USA) Acquity SQD quadrupole Mass instrument, by infusing samples directly into the source using a manual method. The spray voltage was set at 2.5 kV and the capillary temperature at 80 °C. X-band continuous wave electron paramagnetic resonance (CW-EPR) spectra were taken at 100 K using a Bruker EMXplus spectrometer equipped with a dual-mode resonator (ER 4116DM). The low temperature was controlled with the digital temperature system and gas flow control system (ER 4141VT). The experimental parameters for EPR measurement were as follows: Microwave frequency = 9.422 GHz, microwave power = 1.0 mW, modulation amplitude = 5 G, gain = 1.59 × 10^4^, modulation frequency = 100 kHz, time constant = 327.68 ms, and conversion time = 100 ms. Infrared spectra were recorded on a FT-IR spectrometer (Thermo Scientific Nicolet iS10) equipped with a diamond attenuated total reflectance (ATR) accessory. ^1^H NMR spectra were measured with a 600 MHz FT–NMR VNMRS 600 spectrophotometer analyzer at the UNIST Central Research Facilities. The crystallographic analysis was conducted with a R-AXIS RAPID II (Rigaku) equipped with a Mo X-ray tube in UNIST. Product composition was analyzed using a Thermo Fisher Trace 1310 gas chromatograph system equipped with a flame ionization detector.

**Synthesis and Isolation of [Mn^III^(TBDAP)(OH)(C_6_H_5_COO)](OTf) (1-(OTf)). 1** was generated by adding 2 equiv. of benzoic acid into the solution of [Mn^III^(TBDAP)(OH)_2_]^+^ in acetonitrile under N_2_ at 25 °C. [Mn^III^(TBDAP)(OH)_2_]^+^ was synthesized according to the literature method.^S3^ Et_2_O (40 mL) was added to the resulting solution to yield a yellowish green powder, which was washed with Et_2_O, and dried in a vacuo. Yellowish green powder dissolves in CH_3_CN at −40 °C. Yellowish green crystals were obtained by slow diffusion of Et_2_O into the resulting solution at room temperature. UV-Vis in CH_3_CN: *λ*_max_ (*ε*) = 425 nm (90 M^–1^ cm^–1^). ESI-MS in CH_3_CN: *m/z* = 545.2 for [Mn^III^(TBDAP)(OH)(C_6_H_5_COO)]^+^ (calculated *m/z* = 545.2). Anal. Calcd for C_30_H_40_F_3_MnN_4_O_7_S: C, 50.56; H, 5.66; N, 7.86. Found: C, 50.05; H, 5.64; N, 8.00. The effective magnetic moment of *μ*_eff_ = 4.8 *μ*_B_ was determined by the ^1^H NMR Evans method in CD_3_CN at 25 °C.

**Synthesis and Isolation of [Mn^III^(TBDAP)(OH)(C_6_H_5_CH_2_O)](OTf) (2-(OTf)). 2** was generated by adding an excess amount of benzyl alcohol to [Mn^III^(TBDAP)(OH)_2_]^+^ in CH_3_CN at 60 °C. Et_2_O (40 mL) was added to the resulting solution to yield a green powder, which was washed with Et_2_O, and dried in a vacuo. Green powder dissolves in CH_3_CN at −40 °C. UV-Vis in CH_3_CN: *λ*_max_ (*ε*) = 422 nm (217 M^–1^ cm^–1^) and *λ*_max_ (*ε*) = 600 nm (69 M^–1^ cm^–1^). ESI-MS in CH_3_CN: *m/z* = 531.2 for [Mn^III^(TBDAP)(OH)(C_6_H_5_CH_2_O)]^+^ (calculated *m/z* = 531.3). The effective magnetic moment of *μ*_eff_ = 5.2 *μ*_B_ was determined by the ^1^H NMR Evans method in CD_3_CN at 25 °C.

**Synthesis and Isolation of [Mn^III^(TBDAP)(OH)(4-NH_2_−C_6_H_4_COO)](OTf) (3-(OTf)). 3** was synthesized by addition 2 equiv. of NH_2_−C_6_H_4_COOH to the solution of [Mn^III^(TBDAP)(OH)_2_]^+^ in CH_3_CN at 40 °C. Et_2_O (40 mL) was added to the resulting solution to yield a yellowish green powder, which was washed with Et_2_O, and dried in a vacuo. Yellowish green powder dissolves in CH_3_CN at −40 °C. Yellowish green crystals were obtained by slow diffusion of Et_2_O into the resulting solution at −20 °C. UV-Vis in CH_3_CN: *λ*_max_ (*ε*) = 380 nm (390 M^–1^ cm^–1^) and *λ*_max_ (*ε*) = 430 nm (145 M^–1^ cm^–1^). ESI-MS in CH_3_CN: *m/z* = 560.2 for [Mn^III^(TBDAP)(OH)(4-NH_2_−C_6_H_4_COO)]^+^ (calculated *m/z* = 560.2). Anal. Calcd for C_30_H_41_F_3_MnN_5_O_7_S: C, 49.52; H, 5.68; N, 9.62. Found: C, 49.94; H, 5.77; N, 9.59. The effective magnetic moment of *μ*_eff_ = 5.0 *μ*_B_ was determined by the ^1^H NMR Evans method in CD_3_CN at 25 °C.

**Synthesis and Isolation of [Mn^III^(TBDAP)(OH)(4-NO_2_−C_6_H_4_COO)](OTf) (4-(OTf)). 4** was generated by adding 4 equiv. of NO_2_−C_6_H_4_COOH to the solution of [Mn^III^(TBDAP)(OH)_2_]^+^ in CH_3_CN at 0 °C. Et_2_O (40 mL) was added to the resulting solution to yield a yellowish green, which was washed with Et_2_O, and dried in a vacuo. Yellowish green powder dissolves in CH_3_CN at −40 °C. Yellowish green crystals were obtained by slow diffusion of Et_2_O into the resulting solution at −20 °C. UV-Vis in CH_3_CN: *λ*_max_ (*ε*) = 432 nm (86 M^–1^ cm^–1^). ESI-MS in CH_3_CN: *m/z* = 590.2 for [Mn^III^(TBDAP)(OH)(4-NO_2_−C_6_H_4_COO)]^+^ (calculated *m/z* = 590.2). Anal. Calcd for C_30_H_37_F_3_MnN_5_O_8_S: C, 48.72; H, 5.04; N, 9.47. Found: C, 48.60; H, 5.32; N, 9.30. The effective magnetic moment of *μ*_eff_ = 4.9 *μ*_B_ was determined by the ^1^H NMR Evans method in CD_3_CN at 25 °C.

**Magnetic Moment Measurement.** The effective magnetic moments were confirmed by the ^1^H NMR Evans method at 25 °C.^S4^ A WILMAD^®^ coaxial insert (sealed capillary) tube containing the blank acetonitrile-*d*_3_ solvent (with 1.0 % TMS) only was inserted into the normal NMR tube containing the complexes dissolved in acetonitrile-*d*_3_ (with 0.03 % TMS). The chemical shift of the TMS peak (and/or solvent peak) in the presence of the paramagnetic metal complexes was compared to that of the TMS peak (and/or solvent peak) in the inner coaxial insert tube. The effective magnetic moments were calculated using the equation, *μ*_eff_ = 0.0618(Δ*νT*/2*fM*)^1/2^, where *f* is the oscillator frequency (MHz) of the superconducting spectrometer, *T* is the absolute temperature, *M* is the molar concentration of the metal ion, and Δ*v* is the difference in frequency (Hz) between the two reference signals.

**X-ray Crystallography.** Single crystals of **1**-OTf_,_ **3**-OTf·CH_3_CN, and **4**-OTf were isolated from solutions by a nylon loop (Hampton Research Co.) on a hand-made copper plate mounted inside a liquid N_2_ Dewar vessel at *ca*. −40 °C and mounted on a goniometer head in a N_2_ cryostream. Data collections were performed on a Rigaku Rapid Auto CCD diffractometer equipped with a monochromator in the Mo Kα (*λ* = 0.71073 Å) incident beam. The CCD data were integrated and scaled using the Rigaku Rapid Auto software package. The crystal structure was solved by the intrinsic phasing method with SHELX-XT (Ver. 2018/2), and refined by full-matrix least-squares calculation with SHELX-XL (Ver. 2018/3).^S5-7^ All non-hydrogen atoms were refined with anisotropic thermal parameters. The crystallographic data with the selected bond distances and angles for **1**-OTf, **3**-OTf·CH_3_CN, and **4**-OTf are listed in Tables S2 and S3. Hydrogen atoms were located in the calculated positions except H1 for **1**, **3** and **4**, which were found from the Fourier difference map. CCDC-2504550 for **1**-OTf, CCDC-2504551 for **3**-OTf·CH_3_CN and CCDC-2504552 for **4**-OTf contain the supplementary crystallographic data for this paper. These data can be obtained free of charge via www.ccdc.cam.ac.uk/data_request/cif (or from the Cambridge Crystallographic Data Centre, 12, Union Road, Cambridge CB2 1EZ, UK; fax: (+44) 1223-336-033; or [deposit@ccdc.cam.ac.uk](mailto:deposit@ccdc.cam.ac.uk)).

**Spectral Redox Titration for the Equilibrium Constant (*K*_et_) of 1.**

Redox titration of electron transfer from Me_10_Fc to **1** (1.0 mM) was performed in the various concentrations of Me_10_Fc (0.0 – 2.0 mM) in CH_3_CN (2 mM) at 25 °C using UV-vis spectral changes.^S8^ A CH_3_CN solution of Me_10_Fc (0.0 – 2.0 mM) was added to a CH3CN solution containing **1** (1.0 mM) (Figure S4). The concentration of [Me_10_Fc]^+^ was determined by the UV-vis spectral change at 778 nm (Ɛ = 5.4 × 10^2^ M^–1^ cm^–1^). The equilibrium constant (*K*_et_) between **1** and Me_10_Fc is expressed by an equation S1.

*K*_et_ = [**1**^–1^][Me_10_Fc^+^]/[**1**][Me_10_Fc]	(S1)

, where **1**^–1^ denotes the one-electron-reduced **1** (*i.e.*, [Mn^II^(TBDAP)(OH)(C_6_H_5_COO)]). The initial concentrations of [**1**] and [Me_10_Fc] are denoted as [**1**]_0_ and [Me_10_Fc]_0_, respectively, where [**1**]_0_ = [**1**] + [**1**^–1^], [Me_10_Fc]_0_ = [Me_10_Fc] + [Me_10_Fc^+^]. In addition, the concentration of [**1**^-1^] is equal to that of [Me_10_Fc^+^].

Equation S3 is inserted to eq S1, and eq S2 is derived after some rearrangements.

1/*K*_et_ = ([**1**]_0_/[Me_10_Fc^+^] – 1)([Me_10_Fc]_0_/[Me_10_Fc^+^] – 1)	(S2)

Equation S3 is derived from eq S2, where α = [**1**^–1^]/[**1**]_0_ = [Me_10_Fc^+^]/[**1**]_0_.

(α ^–1^ – 1) ^–1^ = *K*_et_([Me_10_Fc]_0_/α[**1**]_0_ – 1)	(S3)

Finally, the *K*_et_ value of 0.43 is determined from the slope of the linear plot between (α^–1^ – 1)^–1^ and [Me_10_Fc]_0_/α[**1**]_0_ – 1 (Figure S5).

**Reactivity Studies.** All reactions were monitored in a 1-cm UV cuvette, with the UV−Vis spectral changes of the reaction solutions under N_2_ atmosphere. Rate constants were determined by fitting the changes in absorbance at 430 nm for the decay of **1**, **3**, and **4**. **1**−**4** exhibited stability for several days at room temperature, which allowed us to use it in reactivity studies. Each reaction was performed at least in triplicate, and the data reported represent the average of these reactions. After the completion of reactions, pseudo-first-order fitting of the kinetic data allowed us to determine *k*_obs_ values. To determine the kinetic isotope effect (KIE), D₂O was employed as the deuterium source, which necessitated the presence of H₂O/D₂O in the reaction medium. Under these conditions, the reaction of **1** with 4-MeO-2,6-DTBP obeyed pseudo-first-order kinetics and afforded a second-order rate constant of *k*₂ = 0.9975 M⁻¹ s⁻¹. This value is slightly slower than that obtained under anhydrous conditions (*k*₂ = 3.4 M⁻¹ s⁻¹), indicating a modest decrease in reaction rate upon the addition of water.

**Product Analysis.** Product analysis was performed by UV−vis spectroscopy and ^1^H NMR spectroscopy. Products formed in the oxidation reactions of 4-MeO-2,6-DTBP with **1** and **2** were analyzed by UV−vis spectroscopy. A dichloromethane solution of 4-MeO-2,6-DTBP was added to a solution of **1** and **2** (0.5 mM) in CH_3_CN. The oxidation product of 2,6-di-*tert*-butyl-4-methoxyphenoxyl radical was produced in yields of 90(3) and 92(2)% for **1** and **2**, respectively. Furthermore, the products formed in the oxidation reactions of 2,4-DTBP with **1**, **3**, and **4** were analyzed by ^1^H NMR spectroscopy. 2,4-DTBP was added to a solution of **1**, **3**, and **4** (1 mM) at 25 °C. After 12 hours, samples were measured. The oxidation product of 2,4-DTBP, 3,3’,5,5’-tetra-tert-butylbiphenyl-2,2’-diol, was produced in 82(12)% for **1**, 79(13)% for **3**, and 88(8)% for **4**, respectively. All product yields were calculated by the electron balance of the substrate oxidations to prevent confusion.

**Computational details.** Theoretical calculations were conducted with density functional theory (DFT)^S9^ by using Gaussian 16 package.^S10^ The unrestricted B3LYP functional was employed for all geometry optimizations with a basis set def2-SVP for all atoms.^S11, S12^ All calculations were carried out in solvent (acetonitrile) using the PCM scheme.^S13^ Single-point calculations with the optimized structure were performed to obtain more accurate electronic energies, and Mulliken spin density and charge distribution values at the UB3LYP/def2-TZVP level.^S11^ Optimized geometries were visualized with *Gaussview 6*. The coordinates obtained from the crystal structure of **1** were used to as the starting input structure. In order to gauge the credibility of the DFT calculations, we compared the optimized geometry of **1** to the crystal structure of **1**. The root mean square deviation (RMSD) value of 0.118 Å, not counting the hydrogen, was obtained giving a good fit to the crystal structure. The one-electron reduction potentials (*E*) and p*K*_a_ value were determined from Born-Haber cycles (Scheme S1).^S14, S15^ The structures of Mn^III^−OH, Mn^III^−OH_2_, Mn^II^−OH, and Mn^II^−OH_2_ are optimized to determine p*K*_a_ and *E*_1/2_. The p*K*_a_ and redox potential were evaluated by equations S4 and S5. A value of −4.28 V was adopted as the reference potential for the standard hydrogen electrode (SHE). Values of −265.9 kcal/mol and −6.28 kcal/mol were employed for the solvation free energy and gas-phase free energy of a proton, respectively.^S15^

p*K*_a_ = [∆*G*_g_ + ∆*G*_s_(A^−^) + ∆*G*_s_(H^+^) − ∆*G*_s_(AH)]/2.303RT	(S4)

*E*°_cal_ = −*G*^o,redox^_solv_/*F* − 4.28; *F* = 23.061 kcal/V	(S5)

**Scheme S1.** Born-Haber Thermodynamic Cycles for Calculating the Free Energy of a) Reduction and b) Deprotonation

**References**

S1. C.-M. Che, Z.-Y. Li, K.-Y. Wong, C.-K. Poon, T. C. W. Mak, S.-M. Peng, *Polyhedron* **1994**, *13*, 771.

S2. Jeong, D.; Ohta, T.; Cho, J. Structure and Reactivity of a Mononuclear Nonheme Manganese(III)–Iodosylarene Complex. *J. Am. Chem. Soc.* **2018**, *140*, 16037–16041.

S3. Lee, Y.; Moon, D.; Cho, J.; Controlling Redox Potential of a Manganese(III)−Bis(Hydroxo) Complex through Protonation and the Hydrogen-Atom Transfer Reactivity. *J. Am. Chem. Soc.* **2024**, *146*, 15795−15805.

S4. Evans, D. F. The Determination of the Paramagnetic Susceptibility of Substance in Solution by Nuclear Magnetic Resonance. *J. Chem. Soc.* **1959**, 2003−2005

S5. Sheldrick, G. M. SHELXTL/PC, ver. 6.12 for Windows XP; Bruker AXS Inc.: Madison, WI, **2001**.

S6. Sheldrick, G. *Acta Crystallogr., Sect. A: Found. Adv.* **2015**, *71*, 3.

S7. Sheldrick, G. *Acta Crystallogr., Sect. C: Found. Adv.* **2015**, *71*, 3.

S8. Hong, S.; Lee, Y.-M.; Sankaralingam, M.; Vardhaman, A. K.; Park, Y. J.; Cho, K.- B.; Ogura, T.; Sarangi, S.; Fukuzumi, S.; Nam, W. A Manganese(V)-Oxo Complex: Synthesis by Dioxygen Activation and Enhancement of Its Oxidizing Power by Binding Scandium Ion. *J. Am. Chem. Soc.* **2016**, *138*, 8523–8532.

S9. Kohn, W.; Sham, L. J. Self-Consistent Equations Including Exchange and Correlation Effects. *Phys. Rev*. **1965**, *140*, A1133− A1138.

S10. M. J. Frisch, G. W. Trucks, H. B. Schlegel, G. E. Scuseria, M. A. Robb, J. R. Cheeseman, G. Scalmani, V. Barone, G. A. Petersson, H. Nakatsuji, X. Li, M. Caricato, A. V. Marenich, J. Bloino, B. G. Janesko, R. Gomperts, B. Mennucci, H. P. Hratchian, J. V. Ortiz, A. F. Izmaylov, J. L. Sonnenberg, D. Williams-Young, F. Ding, F. Lipparini, F. Egidi, J. Goings, B. Peng, A. Petrone, T. Henderson, D. Ranasinghe, V. G. Zakrzewski, J. Gao, N. Rega, G. Zheng, W. Liang, M. Hada, M. Ehara, K. Toyota, R. Fukuda, J. Hasegawa, M. Ishida, T. Nakajima, Y. Honda, O. Kitao, H. Nakai, T. Vreven, K. Throssell, J. A. Montgomery, Jr., J. E. Peralta, F. Ogliaro, M. J. Bearpark, J. J. Heyd, E. N. Brothers, K. N. Kudin, V. N. Staroverov, T. A. Keith, R. Kobayashi, J. Normand, K. Raghavachari, A. P. Rendell, J. C. Burant, S. S. Iyengar, J. Tomasi, M. Cossi, J. M. Millam, M. Klene, C. Adamo, R. Cammi, J. W. Ochterski, R. L. Martin, K. Morokuma, O. Farkas, J. B. Foresman, and D. J. Fox, Gaussian, Inc., Wallingford CT, **2016**.

S11. Weigend, F.; Ahlrichs, R. Balanced Basis Sets of Split Valence, Triple Zeta Valence and Quadruple Zeta Valence Quality for H to Rn: Design and Assessment of Accuracy. *Phys. Chem. Chem. Phys.* **2005**, *7*, 3297−3305.

S12. Becke, A. D. Density-Functional Thermochemistry. III. The Role of Exact Exchange. *J. Chem. Phys*. **1993**, 98, 5648-5652.

S13. Tomasi, J.; Mennucci, B.; Cammi, R. Quantum Mechanical Continuum Solvation Models. *Chem. Rev.* **2005**, *105*, 2999−3094.

S14. Solis, B. H.; Hammes-Schiffer, S. Proton-Coupled Electron Transfer in Molecular Electrocatalysis Theoretical methods and Design Principles. *Inorg. Chem.* **2014**, *53*, 6427–6443

S15. Bai, B.; Pan, Y.; Zhao. J.; Zhang, X. P.; Cao, R. Origins of HCOOH Selectivity Over CO Medicated by an Unusual Fe(I)-Porphyrin Bearing a *β*-Substituted Cation. *Inorg. Chem.* **2025**, *64*, 4826–4834

**Table S1**. Crystal Data and Structure Refinements for **1**-OTf, **3**-OTf·CH_3_CN, and **4**-OTf

|  | **1**-OTf | **3**-OTf·CH_3_CN | **4**-OTf |
| --- | --- | --- | --- |
| Empirical formula | C_30_H_38_F_3_MnN_4_O_6_S | C_32_H_42_F_3_MnN_6_O_6_S | C_30_H_37_F_3_MnN_5_O_8_S |
| Formula weight | 694.64 | 750.71 | 739.64 |
| Temperature (K) | 173(2) | 173(2) | 173(2) |
| Wavelength (Å) | 0.71073 | 0.71073 | 0.71073 |
| Crystal system/space group | Triclinic, *P -1* | Triclinic, *P -1* | Triclinic, *P -1* |
| Unit cell dimensions |  |  |  |
| *a* (Å) | 10.760(2) | 11.667(2) | 11.928(2) |
| *b* (Å) | 11.004(2) | 11.788(2) | 13.958(3) |
| *c* (Å) | 14.965(3) | 14.974(3) | 14.412(3) |
| *α* (º) | 73.62(3) | 110.47(3) | 62.43(3) |
| *β* (º) | 71.68(3) | 106.26(3) | 89.06(3) |
| *γ* (º) | 87.76(3) | 100.33(3) | 76.38(3) |
| Volume (Å^3^) | 1611.6(7) | 1761.8(8) | 2055.1(9) |
| Z | 2 | 2 | 2 |
| Calculated density (g/cm^−3^) | 1.431 | 1.415 | 1.195 |
| Absorption coefficient (mm^−1^) | 0.540 | 0.501 | 0.431 |
| Reflections collected | 12716 | 17318 | 16124 |
| Independent reflections [*R*(int)] | 5654 [0.0277] | 8054 [0.0342] | 7222 [0.0567] |
| Refinement method | Full-matrix  least-squares on *F*^2^ | Full-matrix least-squares on *F*^2^ | Full-matrix least-squares on *F*^2^ |
| Data/restraints/parameters | 5654 / 0 / 416 | 8054 / 0 / 452 | 7222 / 0 / 443 |
| Goodness-of-fit on *F*^2^ | 1.041 | 1.020 | 1.022 |
| Final *R* indices [*I* > 2sigma(*I*)] | *R*_1_ = 0.0361, *wR*_2_ = 0.0782 | *R*_1_ = 0.0569, *wR*_2_ = 0.1312 | *R*_1_ = 0.0578, *wR*_2_ = 0.1148 |
| *R* indices (all data) | *R*_1_ = 0.0479, *wR*_2_ = 0.0828 | *R*_1_ = 0.0819, *wR*_2_ = 0.1431 | *R*_1_ = 0.0879, *wR*_2_ = 0.1259 |
| Largest difference peak and hole (e/Å^3^) | 0.339 and −0.455 | 0.892 and −0.449 | 0.325 and -0.291 |

**Table S2**. Selected Crystal Structure Bond Distances (Å) for **1**-OTf, **3**-OTf·CH_3_CN, and **4**-OTf

| Bond Distances (Å) | | | | | |
| --- | --- | --- | --- | --- | --- |
| **1**-OTf | | **3**-OTf·CH_3_CN | | **4**-OTf | |
| Mn−O1 | 1.799(2) | Mn−O1 | 1.810(2) | Mn−O1 | 1.804(2) |
| Mn−O2 | 1.919(2) | Mn−O2 | 1.923(2) | Mn−O2 | 1.936(2) |
| Mn−N1 | 2.392(2) | Mn−N1 | 2.391(2) | Mn−N1 | 2.388(3) |
| Mn−N2 | 2.040(2) | Mn−N2 | 2.035(2) | Mn−N2 | 2.041(3) |
| Mn−N3 | 2.375(2) | Mn−N3 | 2.401(2) | Mn−N3 | 2.371(3) |
| Mn−N4 | 2.040(2) | Mn−N4 | 2.043(2) | Mn−N4 | 2.029(3) |
| O2−C1 | 1.305(3) | O2−C7 | 1.311(3) | O2−C1 | 1.298(4) |
| O3−C1 | 1.233(2) | O3−C7 | 1.236(3) | O3−C1 | 1.232(4) |
| O1−H1 | 0.70(3) | N5−C4 | 1.364(4) | N5−C5 | 1.470(4) |
|  |  | O1−H1 | 0.80(4) | O1−H1 | 0.80(4) |

**Table S3**. Selected Crystal Structure Angles (º) for **1**-OTf, **3**-OTf·CH_3_CN, and **4**-(OTf)

| Bond Angles (°) | | | | | |
| --- | --- | --- | --- | --- | --- |
| **1**-OTf | | **3**-OTf·CH_3_CN | | **4**-OTf | |
| N1−Mn−N2 | 77.92(6) | N1−Mn−N2 | 75.20(9) | N1−Mn−N2 | 74.78(10) |
| N1−Mn−N3 | 144.13(6) | N1−Mn−N3 | 143.35(8) | N1−Mn−N3 | 143.22(9) |
| N1−Mn−N4 | 75.24(7) | N1−Mn−N4 | 77.91(8) | N1−Mn−N4 | 78.02(10) |
| N1−Mn−O1 | 104.69(7) | N1−Mn−O1 | 98.93(10) | N1−Mn−O1 | 99.65(11) |
| N1−Mn−O2 | 98.76(6) | N1−Mn−O2 | 108.07(8) | N1−Mn−O2 | 106.44(9) |
| N2−Mn−N3 | 75.71(7) | N2−Mn−N3 | 78.27(10) | N2−Mn−N3 | 78.21(10) |
| N2−Mn−N4 | 83.95(7) | N2−Mn−N4 | 85.23(9) | N2−Mn−N4 | 85.50(10) |
| N2−Mn−O1 | 88.73(7) | N2−Mn−O1 | 88.91(10) | N2−Mn−O1 | 89.47(11) |
| N2−Mn−O2 | 173.40(7) | N2−Mn−O2 | 172.53(9) | N2−Mn−O2 | 173.12(10) |
| N3−Mn−N4 | 78.18(7) | N3−Mn−N4 | 75.09(9) | N3−Mn−N4 | 75.39(10) |
| N3−Mn−O1 | 98.61(7) | N3−Mn−O1 | 105.42(9) | N3−Mn−O1 | 104.63(11) |
| N3−Mn−O2 | 104.78(6) | N3−Mn−O2 | 95.77(9) | N3−Mn−O2 | 97.66(9) |
| N4−Mn−O1 | 172.53(7) | N4−Mn−O1 | 173.89(9) | N4−Mn−O1 | 174.86(10) |
| N4−Mn−O2 | 89.69(7) | N4−Mn−O2 | 88.89(9) | N4−Mn−O2 | 88.14(10) |
| O1−Mn−O2 | 97.67(7) | O1−Mn−O2 | 97.09(9) | O1−Mn−O2 | 96.93(10) |
| Mn−O1−H1 | 105(2) | Mn−O1−H1 | 106(3) | Mn−O1−H1 | 112(3) |
| Mn−O2−C1 | 131.0(1) | Mn−O2−C1 | 130.20(18) | Mn−O2−C1 | 130.8(2) |
| O2−C1−O3 | 124.3(2) | O2−C1−O3 | 123.9(3) | O2−C1−O3 | 125.7(3) |

**Table S4.** Energies of the Different Spin States for [Mn^III^(TBDAP)(OH)(C_6_H_5_COO)]^+^ (**1**), [Mn^III^(TBDAP)(OH)(C_6_H_5_CH_2_O)]^+^ (**2**), [Mn^III^(TBDAP)(OH)(NH_2_–C_6_H_4_COO)]^+^ (**3**), and [Mn^III^(TBDAP)(OH)(NO_2_–C_6_H_4_COO)]^+^ (**4**).

|  | ∆(Def2-SVP) | ∆∆(Def2-TZVP) | ∆*E^a^* | ∆Z_0_ | *∆E* (thermal)*^b^* | –T∆S*^b^* | ∆*G^c^* |
| --- | --- | --- | --- | --- | --- | --- | --- |
| **1** |  |  |  |  |  |  |  |
| Singlet | 48.39 | 0.31 | **48.70** | 1.71 | –0.68 | 3.39 | 53.11 |
| Triplet | 26.52 | –0.13 | **26.39** | 1.63 | –0.60 | 2.59 | 30.01 |
| Quintet | 0.00 | 0.00 | **0.00** | 0.00 | 0.00 | 0.00 | 0.00 |
| **2** |  |  |  |  |  |  |  |
| Singlet | 52.11 | 0.18 | **52.29** | 1.32 | –0.64 | 3.16 | 56.14 |
| Triplet | 24.26 | 0.25 | **24.51** | 0.72 | –0.36 | 1.66 | 26.53 |
| Quintet | 0.00 | 0.00 | **0.00** | 0.00 | 0.00 | 0.00 | 0.00 |
| **3** |  |  |  |  |  |  |  |
| Singlet | 49.26 | 0.21 | **49.47** | 1.72 | –0.69 | 3.29 | 53.79 |
| Triplet | 27.08 | –0.22 | **26.86** | 1.79 | –0.66 | 2.72 | 30.71 |
| Quintet | 0.00 | 0.00 | **0.00** | 0.00 | 0.00 | 0.00 | 0.00 |
| **4** |  |  |  |  |  |  |  |
| Singlet | 47.64 | 0.23 | **47.87** | 1.71 | –0.67 | 3.47 | 52.37 |
| Triplet | 18.82 | –0.16 | **18.67** | 1.34 | –0.55 | 2.44 | 21.90 |
| Quintet | 0.00 | 0.00 | **0.00** | 0.00 | 0.00 | 0.00 | 0.00 |

Note: *^a^* Sum of the two previous columns (relative electronic energy at single-point Def2-TZVP level). *^b^* T=298.15 K. *^c^* ∆*G* = ∆*E* + ∆*Z*_0_ + ∆*E*(thermal) –T∆*S*.

**Table S5.** Selected Bond Distance Comparisons (Å) for [Mn^III^(TBDAP)(OH)(C_6_H_5_COO)]^+^ (**1**), [Mn^III^(TBDAP)(OH)(C_6_H_5_CH_2_O)]^+^ (**2**), [Mn^III^(TBDAP)(OH)(NH_2_–C_6_H_4_COO)]^+^ (**3**), and [Mn^III^(TBDAP)(OH)(NO_2_–C_6_H_4_COO)]^+^ (**4**) on the Quintet State.

|  | **1** | **2** | **3** | **4** |
| --- | --- | --- | --- | --- |
| Mn–N1 | 2.44 | 2.48 | 2.44 | 2.43 |
| Mn–N2 | 2.07 | 2.08 | 2.07 | 2.07 |
| Mn–N3 | 2.45 | 2.47 | 2.45 | 2.44 |
| Mn–N4 | 2.07 | 2.07 | 2.07 | 2.06 |
| Mn–O1 | 1.80 | 1.84 | 1.80 | 1.80 |
| Mn–O2 | 1.93 | 1.85 | 1.92 | 1.94 |
| O1–H1 | 0.99 | 0.97 | 0.99 | 0.99 |
| O3···H1 | 1.79 | – | 1.77 | 1.81 |

**Table S6.** Relative Energies for the O–H Bond Activation Reaction of 4-methoxy-2,6-di-*tert*-butylphenol with [Mn^III^(TBDAP)(OH)(C_6_H_5_COO)]^+^ (**1**) in Kcal Mol^–1^.

|  | ∆(Def2-SVP) | ∆∆(Def2-TZVP) | **∆*E****^a^* | ∆*Z*_0_ | ∆*E*(thermal)*^b^* | –T∆*S^b^* | **∆*G****^c^* |
| --- | --- | --- | --- | --- | --- | --- | --- |
| **1** |  |  |  |  |  |  |  |
| ^H^RC | 0.00 | 0.00 | **0.00** | 0.00 | 0.00 | 0.00 | **0.00** |
| ^H^TS | 12.81 | 2.46 | **15.27** | –4.20 | –0.04 | 0.39 | **11.42** |
| ^H^PC | –3.76 | 2.11 | **–1.64** | –1.94 | 0.81 | –3.55 | **–6.32** |

Note: *^a^* Sum of the two previous columns (relative electronic energy at single-point Def2-TZVP level). *^b^* T=298.15 K. *^c^* ∆*G* = ∆*E* + ∆*Z*_0_ + ∆*E*(thermal) –T∆*S*.

**Table S7.** Mulliken Spin Density Distribution for the O–H Bond Activation Reaction of 4-methoxy-2,6-di-*tert*-butylphenol with [Mn^III^(TBDAP)(OH)(C_6_H_5_COO)]^+^ (**1**).

|  | Mn | R–C_6_H_4_COO | OH | 4-MeO-2,6-DTBP | Rest | Sum |
| --- | --- | --- | --- | --- | --- | --- |
| **1** |  |  |  |  |  |  |
| ^H^RC | 3.91 | 0.01 | 0.03 | 0.00 | 0.05 | 4.00 |
| ^H^TS | 4.27 | –0.01 | –0.03 | –0.27 | 0.03 | 3.99 |
| ^H^PC | 4.87 | 0.03 | 0.01 | –0.99 | 0.09 | 4.01 |

**Table S8.** Mulliken Charge Distribution for the O–H bond Activation Reaction of 4-methoxy-2,6-di-*tert*-butylphenol with [Mn^III^(TBDAP)(OH)(C_6_H_5_COO)]^+^ (**1**).

|  | Mn | R–C6H4COO | OH | 4-MeO-2,6-DTBP | Rest | Sum |
| --- | --- | --- | --- | --- | --- | --- |
| **1** |  |  |  |  |  |  |
| ^H^RC | 0.65 | –0.54 | –0.40 | –0.05 | 1.34 | 1.00 |
| ^H^TS | 0.74 | –0.55 | –0.41 | –0.02 | 1.24 | 1.00 |
| ^H^PC | 0.91 | –0.67 | –0.39 | 0.40 | 0.74 | 0.99 |

**Table S9.** DFT-Calculated Relative Energy (in Kcal Mol^–1^) for Addition of a Proton or Electron to [Mn^III^(TBDAP)(OH)(C_6_H_5_COO)]^+^ (**1**) [Mn^III^(TBDAP)(OH)(C_6_H_5_CH_2_O)]^+^ (**2**), [Mn^III^(TBDAP)(OH)(NH_2_–C_6_H_4_COO)]^+^ (**3**), and [Mn^III^(TBDAP)(OH)(NO_2_–C_6_H_4_COO)]^+^ (**4**) on Quintet Spin State.

| Complex | Mn^III^–OH | Mn^III^–OH_2_ | Mn^II^–OH | Mn^II^–OH_2_ |
| --- | --- | --- | --- | --- |
| **1** | 0.00 | –269.38 | –85.56 | –388.74 |
| **2** | 0.00 | –272.65 | –79.38 | –382.03 |
| **3** | 0.00 | –274.78 | –84.11 | –388.43 |
| **4** | 0.00 | –267.97 | –87.52 | –389.72 |

Note: Energies are relative electronic energies at single-point Def2-TZVP level.

**Table S10.** DFT-calculated thermodynamic parameters and BDFEs for [Mn^III^(TBDAP)(OH)(C_6_H_5_COO)]^+^ (**1**) [Mn^III^(TBDAP)(OH)(C_6_H_5_CH_2_O)]^+^ (**2**), [Mn^III^(TBDAP)(OH)(NH_2_–C_6_H_4_COO)]^+^ (**3**), and [Mn^III^(TBDAP)(OH)(NO_2_–C_6_H_4_COO)]^+^ (**4**).

| Complex | p*K*_a_(Mn^III^–OH_2_) | *E*(Mn^III/II^–OH_2_) | p*K*_a_(Mn^II^–OH_2_) | *E*(Mn^III/II^–OH) | BDFE*^a^* |
| --- | --- | --- | --- | --- | --- |
| **1** | –6.19 | 1.07 | 18.16 | –0.37 | 65.52 |
| **2** | –4.86 | 0.61 | 16.52 | –0.65 | 56.76 |
| **3** | –3.19 | 0.86 | 19.05 | –0.46 | 64.75 |
| **4** | –8.07 | 1.17 | 16.90 | –0.31 | 65.19 |

Note: *^a^* BDFE = ∆*G* (H) + ∆*G* (Mn^III^–OH) – ∆*G* (Mn^II^–OH_2_) + 1.89 kcal/mol + RT; R = 0.01987 kcal/K mol; T = 298.15 K.

**Table S11.** Relative Energies for the O–H Bond Activation Reaction of 2,4-di-*tert*-butylphenol with [Mn^III^(TBDAP)(OH)(C_6_H_5_COO)]^+^ (**1**), [Mn^III^(TBDAP)(OH)(NH_2_–C_6_H_4_COO)]^+^ (**3**), and [Mn^III^(TBDAP)(OH)(NO_2_–C_6_H_4_COO)]^+^ (**4**) in Kcal Mol^–1^.

|  | ∆(Def2-SVP) | ∆∆(Def2-TZVP) | **∆*E****^a^* | ∆*Z*_0_ | ∆*E*(thermal)*^b^* | –T∆*S^b^* | **∆*G****^c^* |
| --- | --- | --- | --- | --- | --- | --- | --- |
| **1** |  |  |  |  |  |  |  |
| ^H^RC | 0.00 | 0.00 | **0.00** | 0.00 | 0.00 | 0.00 | **0.00** |
| ^H^TS | 14.99 | 1.83 | **16.82** | –3.64 | –0.15 | 0.97 | **14.00** |
| ^H^IM | 8.30 | 1.77 | **10.07** | –1.50 | 0.64 | –1.98 | **7.23** |
| **3** |  |  |  |  |  |  |  |
| ^NH2^RC | 0.00 | 0.00 | **0.00** | 0.00 | 0.00 | 0.00 | **0.00** |
| ^NH2^TS | 15.35 | 1.74 | **17.09** | –3.42 | –0.15 | 0.97 | **14.49** |
| ^NH2^IM | 9.28 | 1.70 | **10.99** | –2.01 | 0.79 | –2.58 | **7.19** |
| **4** |  |  |  |  |  |  |  |
| ^NO2^RC | 0.00 | 0.00 | **0.00** | 0.00 | 0.00 | 0.00 | **0.00** |
| ^NO2^TS | 14.66 | 1.87 | **16.53** | –3.84 | –0.09 | 0.53 | **13.12** |
| ^NO2^IM | 7.17 | 1.86 | **9.03** | –1.37 | 0.60 | –2.39 | **5.86** |

Note: *^a^* Sum of the two previous columns (relative electronic energy at single-point Def2-TZVP level). *^b^* T=298.15 K. *^c^* ∆*G* = ∆*E* + ∆*Z*_0_ + ∆*E*(thermal) –T∆*S*.

**Table S12.** Mulliken Spin Density Distribution for the O–H Bond Activation Reaction of 2,4-di-*tert*-butylphenol with [Mn^III^(TBDAP)(OH)(C_6_H_5_COO)]^+^ (**1**), [Mn^III^(TBDAP)(OH)(NH_2_–C_6_H_4_COO)]^+^ (**3**), and [Mn^III^(TBDAP)(OH)(NO_2_–C_6_H_4_COO)]^+^ (**4**).

|  | Mn | R–C_6_H_4_COO | OH | 2,4-DTBP | Rest | Sum |
| --- | --- | --- | --- | --- | --- | --- |
| **1** |  |  |  |  |  |  |
| ^H^RC | 3.92 | 0.01 | 0.03 | 0.00 | 0.05 | 4.01 |
| ^H^TS | 4.36 | 0.00 | –0.02 | –0.36 | 0.03 | 4.01 |
| ^H^IM | 4.87 | 0.02 | 0.02 | –0.99 | 0.09 | 4.01 |
| **3** |  |  |  |  |  |  |
| ^NH2^RC | 3.91 | 0.01 | 0.03 | 0.00 | 0.05 | 4.00 |
| ^NH2^TS | 4.37 | 0.00 | –0.02 | –0.38 | 0.03 | 4.00 |
| ^NH2^IM | 4.86 | 0.03 | 0.02 | –1.00 | 0.09 | 4.00 |
| **4** |  |  |  |  |  |  |
| ^NO2^RC | 3.92 | 0.01 | 0.03 | 0.00 | 0.05 | 4.01 |
| ^NO2^TS | 4.36 | 0.00 | –0.02 | –0.36 | 0.03 | 4.01 |
| ^NO2^IM | 4.87 | 0.02 | 0.01 | –0.99 | 0.09 | 4.00 |

**Table S13.** Mulliken Charge Distribution for the O–H Bond Activation Reaction of 2,4-di-*tert*-butylphenol with [Mn^III^(TBDAP)(OH)(C_6_H_5_COO)]^+^ (**1**), [Mn^III^(TBDAP)(OH)(NH_2_–C_6_H_4_COO)]^+^ (**3**), and [Mn^III^(TBDAP)(OH)(NO_2_–C_6_H_4_COO)]^+^ (**4**).

|  | Mn | R–C_6_H_4_COO | OH | 2,4-DTBP | Rest | Sum |
| --- | --- | --- | --- | --- | --- | --- |
| **1** |  |  |  |  |  |  |
| ^H^RC | 0.64 | –0.50 | –0.45 | –0.03 | 1.34 | 1.00 |
| ^H^TS | 0.70 | –0.54 | –0.36 | –0.01 | 1.21 | 1.00 |
| ^H^IM | 0.84 | –0.65 | –0.38 | 0.44 | 0.75 | 1.00 |
| **3** |  |  |  |  |  |  |
| ^NH2^RC | 0.65 | –0.47 | –0.45 | –0.03 | 1.31 | 1.01 |
| ^NH2^TS | 0.70 | –0.51 | –0.36 | –0.01 | 1.19 | 1.01 |
| ^NH2^IM | 0.83 | –0.63 | –0.39 | 0.44 | 0.75 | 1.00 |
| **4** |  |  |  |  |  |  |
| ^NO2^RC | 0.64 | –0.53 | –0.45 | –0.03 | 1.37 | 1.00 |
| ^NO2^TS | 0.70 | –0.57 | –0.37 | 0.01 | 1.23 | 1.00 |
| ^NO2^IM | 0.85 | –0.67 | –0.38 | 0.44 | 0.76 | 1.00 |

**Table S14.** Thermodynamic Parameters of **1**–**4** Obtained from Experiments and DFT Calculations.

| **Parameter** | **1** | **2** | **3** | **4** | **Method** |
| --- | --- | --- | --- | --- | --- |
| *E*_red_(Mn^III/II^−OH) / V vs. SCE | −0.12 | < −0.60 | – | – | Experimental |
| *E*(Mn^III/II^−OH) / V vs. SHE | −0.37 | −0.65 | −0.46 | −0.31 | DFT |
| *E*(Mn^III/II^−OH_2_) / V vs. SHE | 1.07 | 0.61 | 0.86 | 1.17 | DFT |
| p*K*_a_(Mn^III^−OH_2_) | −6.19 | −4.86 | −3.19 | −8.07 | DFT |
| p*K*_a_(Mn^II^−OH_2_) | 18.16 | 16.52 | 19.05 | 16.90 | DFT |
| O−H BDFE / kcal mol^−1^ | 65.5 | 56.8 | 64.8 | 65.2 | DFT |

Experimental potentials are referenced to SCE and calculated potentials to SHE. Calculated values were obtained from the relative electronic energies in Table S9.

**Figure S1.** UV-vis spectral changes observed in the formation of **1** (1 mM, black) with 2 equiv. benzoic acid in CH_3_CN at 25 °C from [Mn^III^(TBDAP)(OH)_2_]^+^ (red). Inset shows spectral titration for the formation of **1** (1 mM) upon addition of benzoic acid (0.0 – 4.0 mM) to a solution containing [Mn^III^(TBDAP)(OH)_2_]^+^ (1 mM).

**Figure S2.** Electron density contour map of **1** calculated without hydrogen atoms. Based on the difference map, the hydrogen atoms located on the hydroxo groups are refined.

**Figure S3.** Top: Full FT-IR spectrum (ATR) for **1** (red) and **2** (blue). Bottom: FT-IR spectrum (ATR) for **1** (red) and **2** (blue) showing the region between 4000 to 2500 cm^–1^.

**Figure S4.** UV-vis spectral changes observed in the reaction of **1** (1.0 mM) with Me_10_Fc (1.0 mM) in CH_3_CN at 25 °C. Inset shows the time course monitored at 778 nm, corresponding to the formation of Me_10_Fc^+^.

**Figure S5.** Plot of (𝛼^-1^-1)^-1^ against [Me_10_Fc]_0_ / 𝛼 [**1**]_0_-1 to determine the equilibrium constant (*K*_eq_) between **1** and Me_10_Fc in CH_3_CN at 25 °C. *K*_et_ = [Me_10_Fc^+^][LMn^II^(OH)(H−C_6_H_4_COO)] / [Me_10_Fc][LMn^III^(OH)(H−C_6_H_4_COO)]^+^ and 𝛼 = [Me_10_Fc^+^]/[**1**]_0_.

**Figure S6.** UV-vis spectral changes observed in the reaction of **2** (1.0 mM) with Me_10_Fc (1.0 mM) in CH_3_CN at 25 °C. Inset shows the time course monitored at 778 nm due to the formation of Me_10_Fc^+^.

**Figure S7.** Plot of *k*_obs_ against 4-MeO-2,6-DTBP concentration to determine a second-order rate constant of **1** (1 mM) at 25 ℃

**Figure S8.** ESI-MS spectrum of **1** (1 mM) upon addition of 4-MeO-2,6-DTBP (50 mM) in CH_3_CN at 25 °C. The reaction solution was isolated with diethyl ether, and the ESI-MS data of the isolated powder shows a prominent peak at *m/z* = 528.2 corresponds to [Mn^II^(TBDAP)(H−C_6_H_4_COO)]^+^, (calculated *m/z* = 528.2). Inset shows the simulated (blue) and experimental (red) isotope distribution patterns.

**Figure S9.** (a) UV−vis spectral changes of **2** (1 mM) upon addition of 4-MeO-2,6-DTBP (200 mM) in CH_3_CN at 60 °C. (b) Plot of *k*_obs_ against 4-MeO-2,6-DTBP concentration to determine a second-order rate constant of **2** (1 mM) at 60 ℃.

**Figure S10.** Reaction of **1** with 4-MeO-2,6-DTBP in CH_3_CN. (a) Plot of *k*_obs_ against 4-MeO-2,6-DTBP concentration to determine a second-order rate constant of **1** (1 mM) at –10 ℃. (b) Plot of the second-order rate constants for the reaction between **1** (1 mM) and 4-MeO-2,6-DTBP against 1/T at various temperatures to determine the activation parameters.

**Figure S11.** Energy profile (in kcal mol^−1^) for reaction with 4-MeO-2,6-DTBP by **1**.

**Figure S12.** Thermodynamic analysis of **1**.

**Figure S13.** Thermodynamic analysis of **2**.

**Figure S14.** (a) UV−vis absorption spectra of **3** (1 mM) (left), **4** (1 mM) (right) in CH_3_CN at 25 ℃. (b) The peaks at *m/z* = 560.2 and 590.2 correspond to [Mn^III^(TBDAP)(OH)(NH_2_−C_6_H_4_COO)]^+^ (calculated *m/z* = 560.2) (left), [Mn^III^(TBDAP)(OH)(NO_2_−C_6_H_4_COO)]^+^ (calculated *m/z* = 590.2) (right), respectively. Inset shows the simulated (blue) and experimental (red) isotope distribution patterns.

**Figure S15.** X-band EPR spectrum of **3** (blue) and **4** (red). The EPR spectra of **3** and **4** were silent (1.0 mM). The spectra were recorded in frozen CH_3_CN at 100 K.

**Figure S16.** (a) UV−vis spectra of **1** (1 mM), recorded following the addition of 2,4-DTBP (150 mM) in CH_3_CN at 25 °C. Inset shows the time course of the absorption band at 430 nm. (b) ESI-MS spectrum of **1** (left) in CH_3_CN at 25 ℃. After reaction, the ESI-MS data shows peak of [Mn^II^(TBDAP)]^2+^ species. The peak at *m/z =* 528.4 corresponds to [Mn^II^(TBDAP)(H−C_6_H_4_COO)]^+^, (calculated *m/z* = 528.2).

**Figure S17.** Overlayed ^1^H NMR spectra of (top) the reaction mixture of **1** (1 mM) with 2-4DTBP (10 mM) in CDCl_3_, (middle) the blank solution containing 2-4DTBP (10 mM) in CDCl_3_, and (bottom) authentic 3,3’,5,5’-tetra-tert-butylbiphenyl-2,2’-diol in CDCl_3_.

**Figure S18.** (a) UV−vis spectral changes of **3** (left) and **4** (right) (1 mM) upon addition of 2,4-DTBP (150 mM) in CH_3_CN at 25 °C. Inset shows the time course of the absorption band at 430 nm. (b) ESI-MS spectrum of **3** (left), **4** (right) in CH_3_CN at 25 ℃. After reaction, the ESI-MS data also shows peak of [Mn^II^(TBDAP)]^2+^ species. The mass peaks at *m/z* = 543.4 and 573.3 correspond to [Mn^II^(TBDAP)(NH_2_−C_6_H_4_COO)]^+^ (calculated *m/z* = 543.2) (left), [Mn^II^(TBDAP)(NO_2_−C_6_H_4_COO)]^+^ (calculated *m/z* = 573.2) (right), respectively.

**Figure S19.** Thermodynamic analysis of **3**.

**Figure S20.** Thermodynamic analysis of **4**.

**Figure S21.** Energy profile (in kcal mol^−1^) for reaction with 2,4-DTBP by **1**.

**Figure S22.** Energy profile (in kcal mol^−1^) for reaction with 2,4-DTBP by **3**.

**Figure S23.** Energy profile (in kcal mol^−1^) for reaction with 2,4-DTBP by **4**.

**Optimized xyz coordination**

(Charge/Multiplicity) is in parenthesis.

**^H^RC** (1/5) for 2,4-DTBP

Mn -0.278398 0.927649 -1.063996

O 0.662201 -2.445362 1.231286

O -2.621726 -1.307608 -1.677124

O -1.599195 0.547594 -2.411494

O -0.704250 -0.479198 0.026741

N 0.338675 2.520483 -2.211929

N 1.848721 0.132688 -1.956341

N 1.135330 1.584810 0.273940

N -1.376282 2.871327 -0.086279

C 2.983830 -4.372370 1.574576

C 2.392862 -5.854411 3.483815

C 0.691214 -5.446774 1.712920

C 1.833397 -4.786948 2.523102

C 0.366563 -1.128138 7.603689

C 2.427368 -2.525560 7.397654

C 0.157540 -3.616418 7.519398

C 0.946337 -2.405354 6.968977

C 0.851448 -2.380095 5.429873

C 1.334913 -3.468099 4.676098

C 1.294880 -3.548905 3.275987

C 0.296590 -1.314965 4.711317

C 0.727213 -2.440944 2.587804

C 0.238660 -1.347630 3.315746

C -1.660606 3.609915 -1.341239

C -0.482647 3.584584 -2.291527

C -0.219746 4.609055 -3.201238

C 0.907235 4.506849 -4.020398

C 1.774435 3.422751 -3.875120

C 1.472757 2.439349 -2.930160

C 3.224480 -1.563619 -3.250636

C 1.113384 -0.594096 -4.227959

C 0.988318 -2.206791 -2.331799

C 1.805375 -1.049855 -2.930752

C 2.454059 1.344967 -2.566207

C 2.506993 -0.195182 -0.667552

C 2.277032 0.879691 0.373674

C 3.191523 1.167805 1.387552

C 2.896995 2.197909 2.284087

C 1.735817 2.954602 2.115888

C 0.869526 2.635701 1.067797

C -0.290656 3.531748 0.682432

C -3.321846 3.957795 1.127694

C -2.213519 1.885143 2.049691

C -2.627137 2.628383 0.767483

C -3.624826 1.730860 0.017477

C -3.221396 0.676925 -4.680946

C -4.055151 0.710648 -5.801126

C -5.036060 -0.272670 -5.974667

C -5.183237 -1.291741 -5.025442

C -4.351420 -1.327338 -3.905446

C -3.364492 -0.343327 -3.725698

C -2.487465 -0.405095 -2.510802

H 3.818716 -3.932892 2.144815

H 3.371328 -5.254961 1.038702

H 2.651400 -3.637751 0.830229

H 0.147329 -1.669375 0.881743

H 2.759952 -6.712701 2.899246

H 3.238215 -5.476138 4.079766

H 1.626087 -6.232669 4.177823

H 0.254829 -4.748780 0.987189

H 1.071866 -6.324734 1.164561

H -0.111000 -5.792974 2.385078

H -0.699986 -0.997055 7.362079

H 0.453409 -1.183905 8.700397

H 0.904341 -0.225132 7.274187

H 3.014803 -1.668433 7.030505

H 2.511198 -2.549042 8.496963

H 2.893527 -3.444000 7.008976

H 0.550400 -4.570482 7.135541

H 0.218390 -3.649795 8.619972

H -0.906523 -3.553554 7.239673

H -0.194491 -0.508880 2.767089

H 1.766243 -4.306523 5.220871

H -0.101128 -0.437621 5.222568

H -2.514849 3.132948 -1.841083

H -1.939484 4.661089 -1.157777

H -0.882590 5.473620 -3.257949

H 1.124599 5.288053 -4.751845

H 2.685572 3.346592 -4.470456

H 3.879571 -0.779402 -3.659566

H 3.715318 -1.997207 -2.366676

H 3.158548 -2.358096 -4.008715

H 0.120536 -0.171396 -4.021382

H 1.700532 0.145728 -4.790538

H 0.981728 -1.464405 -4.887135

H 1.441405 -2.627137 -1.423650

H -0.039082 -1.907157 -2.091777

H 0.939243 -3.018726 -3.072143

H 3.153409 1.799727 -1.845232

H 3.072561 1.102246 -3.440089

H 2.084061 -1.132613 -0.281270

H 3.593549 -0.348335 -0.776924

H 4.119843 0.599876 1.464581

H 3.588903 2.431078 3.096202

H 1.511736 3.797050 2.772212

H 0.148956 4.334587 0.067723

H -0.670696 4.040573 1.577674

H -2.648163 4.666244 1.633563

H -3.745178 4.457159 0.243348

H -4.154980 3.754738 1.816678

H -1.676083 0.959868 1.799621

H -1.592228 2.494127 2.721531

H -3.119176 1.613473 2.611279

H -3.966959 2.159607 -0.934909

H -3.208835 0.733274 -0.168793

H -4.518303 1.603462 0.646161

H -2.453484 1.438227 -4.536985

H -3.940317 1.505866 -6.541729

H -5.687798 -0.245109 -6.851686

H -5.949078 -2.059394 -5.160563

H -4.453491 -2.113866 -3.155798

H -1.451766 -0.959487 -0.412965

**^H^TS** (1/5) for 2,4-DTBP

Mn 5.640963 3.741820 4.652752

O 6.776126 0.634725 7.017926

O 3.513122 1.239204 4.161967

O 4.197456 3.218959 3.362863

O 5.324020 2.163548 5.810133

N 6.123835 5.400734 3.456039

N 7.862159 3.143124 3.723820

N 7.084527 4.576717 5.964387

N 4.456068 5.634628 5.645907

C 8.275428 -2.004910 7.273535

C 7.128177 -3.298798 9.068308

C 5.751157 -2.232942 7.284082

C 7.011295 -2.055174 8.166215

C 6.661535 1.637517 13.412964

C 8.139320 -0.364348 13.163802

C 5.629046 -0.626782 13.167061

C 6.803197 0.273614 12.712982

C 6.787742 0.403282 11.180378

C 6.904087 -0.754112 10.374495

C 6.893951 -0.751422 8.979937

C 6.659592 1.637119 10.514400

C 6.770315 0.530499 8.314473

C 6.654731 1.696838 9.128803

C 4.070228 6.322990 4.385833

C 5.219055 6.392659 3.397190

C 5.376631 7.431746 2.478650

C 6.490633 7.413040 1.634110

C 7.446729 6.403629 1.762900

C 7.244298 5.404166 2.719754

C 9.375411 1.636345 2.337013

C 7.069087 2.266708 1.535075

C 7.355995 0.682516 3.444287

C 7.926230 1.944466 2.771538

C 8.325066 4.398370 3.073881

C 8.573039 2.907078 5.006707

C 8.294407 3.994590 6.024373

C 9.227826 4.409545 6.976121

C 8.876307 5.439982 7.852571

C 7.638782 6.073186 7.721284

C 6.758662 5.628865 6.729897

C 5.506230 6.403723 6.363662

C 2.471513 6.584652 6.915100

C 3.779235 4.628869 7.829153

C 3.260582 5.310373 6.549737

C 2.310327 4.320329 5.855665

C 2.458738 3.050919 1.174607

C 1.569757 2.933844 0.103427

C 0.738152 1.812452 0.002158

C 0.797267 0.806531 0.974531

C 1.685481 0.922061 2.045246

C 2.522566 2.045175 2.153128

C 3.469065 2.150172 3.315619

H 9.184565 -1.909896 7.889873

H 8.362460 -2.935243 6.687821

H 8.236828 -1.156731 6.578426

H 5.986564 1.524786 6.382501

H 7.204832 -4.198853 8.438233

H 8.024817 -3.267075 9.707142

H 6.248091 -3.425944 9.718157

H 5.623601 -1.386063 6.598258

H 5.833367 -3.157154 6.688077

H 4.846761 -2.315630 7.908971

H 5.712797 2.134590 13.156757

H 6.677495 1.497520 14.505172

H 7.486427 2.319918 13.154632

H 8.995907 0.259333 12.861660

H 8.162697 -0.465888 14.261314

H 8.282671 -1.367082 12.733411

H 5.694778 -1.637608 12.736704

H 5.630097 -0.730862 14.264580

H 4.661279 -0.193729 12.867321

H 6.561383 2.659355 8.621240

H 7.002665 -1.711048 10.884716

H 6.565300 2.566346 11.076937

H 3.250162 5.759412 3.919828

H 3.700552 7.346871 4.562978

H 4.647933 8.242651 2.433419

H 6.628905 8.205048 0.894877

H 8.347881 6.400675 1.147305

H 9.853950 2.473997 1.808301

H 10.011625 1.358906 3.190839

H 9.369003 0.781530 1.644529

H 6.034653 2.505584 1.820087

H 7.469577 3.104929 0.946601

H 7.047340 1.390710 0.870430

H 7.986139 0.319098 4.267869

H 6.340574 0.829578 3.832314

H 7.314568 -0.121547 2.695081

H 9.006279 4.928137 3.760400

H 8.931805 4.196693 2.181759

H 8.223622 1.955221 5.432635

H 9.665074 2.822970 4.874928

H 10.212072 3.940782 7.021498

H 9.581872 5.771969 8.617327

H 7.367305 6.916881 8.358167

H 5.853233 7.225965 5.716381

H 5.110678 6.902407 7.258082

H 3.101475 7.354214 7.386624

H 1.980191 7.032349 6.038248

H 1.680787 6.327487 7.635331

H 4.370637 3.734898 7.585454

H 4.382767 5.295941 8.460784

H 2.918825 4.308520 8.434744

H 1.900622 4.700692 4.909706

H 2.795571 3.354261 5.669040

H 1.454569 4.135441 6.521585

H 3.111877 3.920279 1.262945

H 1.524525 3.718952 -0.655407

H 0.042610 1.721823 -0.836179

H 0.148484 -0.069393 0.896174

H 1.743796 0.147492 2.811731

H 4.625072 1.625524 5.333946

**^H^IM** (1/5) for 2,4-DTBP

Mn -0.261915 1.037998 -1.134763

O 0.816398 -2.351201 1.497408

O -2.778341 -1.269366 -1.292684

O -1.957916 0.555511 -2.305611

O -0.768415 -0.575444 0.197514

N 0.446755 2.576197 -2.544995

N 1.888609 0.100873 -2.184830

N 1.480600 1.813977 -0.008402

N -0.991950 3.254503 -0.260707

C 3.626019 -3.406464 2.163823

C 3.275923 -5.353090 3.687010

C 1.922445 -5.199538 1.594833

C 2.559962 -4.372106 2.739362

C -1.112843 -2.410807 7.629821

C 1.333053 -2.933952 7.764299

C -0.336547 -4.760214 7.240624

C 0.013830 -3.266921 7.023608

C 0.202139 -3.007394 5.524445

C 1.206276 -3.721873 4.811608

C 1.469732 -3.566380 3.461519

C -0.589574 -2.077125 4.803529

C 0.651457 -2.586750 2.726453

C -0.378457 -1.875498 3.460573

C -1.356519 3.879309 -1.559618

C -0.278630 3.701707 -2.617101

C -0.013283 4.657024 -3.601541

C 1.026831 4.409455 -4.503310

C 1.812503 3.264072 -4.360714

C 1.506989 2.364725 -3.331179

C 3.094001 -1.693862 -3.531813

C 0.825784 -0.839196 -4.225699

C 1.063822 -2.282574 -2.208219

C 1.733924 -1.163380 -3.025891

C 2.453829 1.228813 -2.967273

C 2.642669 -0.118694 -0.924813

C 2.607171 1.085658 0.000846

C 3.687568 1.456264 0.805920

C 3.566607 2.605067 1.594163

C 2.414412 3.388658 1.506223

C 1.383768 2.972071 0.654385

C 0.220021 3.892970 0.312808

C -2.710509 4.616182 1.022784

C -1.654361 2.569215 2.047228

C -2.150415 3.207133 0.735507

C -3.286096 2.318888 0.197878

C -3.961520 0.471855 -4.273297

C -4.984092 0.403346 -5.223130

C -5.950916 -0.605636 -5.140534

C -5.891384 -1.546673 -4.105416

C -4.868275 -1.478251 -3.157160

C -3.895002 -0.468554 -3.232609

C -2.799832 -0.400959 -2.195197

H 4.101877 -2.823786 2.969079

H 4.414637 -3.984672 1.655525

H 3.188675 -2.707178 1.440330

H -0.211084 -1.254842 0.653912

H 4.041142 -5.907538 3.122394

H 3.787687 -4.835196 4.513120

H 2.584098 -6.093277 4.118470

H 1.433794 -4.553485 0.854775

H 2.701626 -5.788317 1.084216

H 1.174102 -5.904579 1.991583

H -2.087872 -2.622739 7.164301

H -1.204530 -2.633059 8.703932

H -0.909857 -1.333105 7.531765

H 1.607216 -1.875916 7.628048

H 1.210171 -3.118018 8.843555

H 2.173419 -3.551751 7.414266

H 0.456763 -5.430679 6.877870

H -0.473973 -4.957170 8.315839

H -1.271629 -5.026395 6.723120

H -0.981643 -1.164041 2.892729

H 1.801318 -4.437644 5.375255

H -1.373834 -1.512346 5.306285

H -2.277802 3.402882 -1.923707

H -1.569774 4.957723 -1.460565

H -0.598884 5.576564 -3.655045

H 1.245890 5.128158 -5.296356

H 2.663503 3.081447 -5.019861

H 3.621364 -0.975974 -4.177594

H 3.763779 -1.965929 -2.701970

H 2.929693 -2.603218 -4.129176

H -0.157840 -0.479210 -3.892091

H 1.261786 -0.086240 -4.898156

H 0.671509 -1.750870 -4.821844

H 1.710436 -2.670325 -1.408362

H 0.117115 -1.959867 -1.757803

H 0.846107 -3.126014 -2.879871

H 3.258524 1.702482 -2.380130

H 2.951604 0.879602 -3.881987

H 2.191091 -0.969656 -0.394353

H 3.699636 -0.383322 -1.106196

H 4.605724 0.866112 0.806956

H 4.387002 2.908757 2.248487

H 2.325775 4.321247 2.066977

H 0.627851 4.604304 -0.424461

H -0.020191 4.512022 1.187994

H -1.935422 5.313380 1.376479

H -3.190765 5.057753 0.136701

H -3.475716 4.553628 1.810901

H -1.227096 1.572080 1.867635

H -0.908935 3.183499 2.571641

H -2.508176 2.449837 2.730433

H -3.706620 2.680251 -0.750737

H -2.958839 1.279264 0.062508

H -4.106226 2.316078 0.931268

H -3.202241 1.253735 -4.326084

H -5.028853 1.138812 -6.030537

H -6.751641 -0.658519 -5.882894

H -6.645624 -2.335024 -4.038702

H -4.807902 -2.202974 -2.343451

H -1.552471 -1.009748 -0.260629

**^NH2^RC** (1/5) for 2,4-DTBP

Mn -0.168849 0.919390 -0.930040

O 0.785730 -2.437319 1.372182

O -2.491025 -1.324127 -1.514027

O -1.481446 0.532985 -2.266411

O -0.585933 -0.485962 0.166349

N 0.444484 2.516331 -2.082133

N 1.964476 0.133511 -1.826868

N 1.248353 1.587409 0.402863

N -1.268668 2.867003 0.047062

C 3.109992 -4.358578 1.725243

C 2.524727 -5.827576 3.646332

C 0.819272 -5.435078 1.875639

C 1.961989 -4.767869 2.678984

C 0.497555 -1.074783 7.735396

C 2.560543 -2.469965 7.536313

C 0.292896 -3.563933 7.669526

C 1.078652 -2.355520 7.109021

C 0.981373 -2.341552 5.569908

C 1.465138 -3.434277 4.823163

C 1.423043 -3.525099 3.423731

C 0.424084 -1.282384 4.844442

C 0.852817 -2.422918 2.728011

C 0.364045 -1.325038 3.449235

C -1.556827 3.600280 -1.209629

C -0.380008 3.577566 -2.161580

C -0.122400 4.601962 -3.073118

C 1.003048 4.502218 -3.894684

C 1.873675 3.420824 -3.749886

C 1.576838 2.437856 -2.802687

C 3.340786 -1.558171 -3.126103

C 1.218877 -0.599728 -4.092154

C 1.112593 -2.210042 -2.193471

C 1.921216 -1.050295 -2.798657

C 2.562939 1.346820 -2.440103

C 2.624746 -0.189342 -0.538295

C 2.393647 0.887816 0.500513

C 3.311066 1.182780 1.509882

C 3.015733 2.214086 2.404752

C 1.850694 2.965172 2.238734

C 0.981797 2.639595 1.194647

C -0.183363 3.530948 0.812286

C -3.213444 3.948881 1.266100

C -2.099314 1.876811 2.182663

C -2.517025 2.621186 0.902480

C -3.515025 1.722910 0.153520

C -3.122721 0.637072 -4.533570

C -3.953312 0.669500 -5.645031

C -4.954173 -0.317595 -5.831214

C -5.075134 -1.336307 -4.851238

C -4.238612 -1.357023 -3.745451

C -3.245629 -0.373958 -3.559610

C -2.373428 -0.426973 -2.362792

H 3.945443 -3.913957 2.290671

H 3.497616 -5.244420 1.194824

H 2.774825 -3.629649 0.976554

H 0.267905 -1.664496 1.016183

H 2.891994 -6.689570 3.067329

H 3.370608 -5.443802 4.238016

H 1.759767 -6.201897 4.344474

H 0.380616 -4.742634 1.145977

H 1.200124 -6.316325 1.332690

H 0.018815 -5.777693 2.551730

H -0.569568 -0.947310 7.494387

H 0.586089 -1.122485 8.832358

H 1.033233 -0.173215 7.398609

H 3.145834 -1.614379 7.162207

H 2.646156 -2.485549 8.635636

H 3.027782 -3.390246 7.153331

H 0.686953 -4.520077 7.292122

H 0.355371 -3.589131 8.770236

H -0.771684 -3.505094 7.390882

H -0.071107 -0.490975 2.895208

H 1.898430 -4.268230 5.373260

H 0.025951 -0.401913 5.350001

H -2.408718 3.117028 -1.707330

H -1.840645 4.650859 -1.029606

H -0.788159 5.464348 -3.129560

H 1.216442 5.283093 -4.627684

H 2.783668 3.346489 -4.347260

H 3.988776 -0.772570 -3.543667

H 3.840275 -1.984274 -2.243289

H 3.274065 -2.357037 -3.879536

H 0.223595 -0.186023 -3.878947

H 1.796877 0.145684 -4.656936

H 1.091693 -1.470675 -4.751448

H 1.575803 -2.630239 -1.290304

H 0.086951 -1.912621 -1.943265

H 1.058827 -3.021313 -2.934295

H 3.263177 1.805085 -1.722165

H 3.179388 1.105430 -3.315918

H 2.203592 -1.126324 -0.148986

H 3.711644 -0.341005 -0.647828

H 4.242196 0.619145 1.584768

H 3.709771 2.452539 3.213530

H 1.625475 3.808513 2.893550

H 0.251443 4.335608 0.196454

H -0.562244 4.038448 1.708894

H -2.540735 4.656741 1.774153

H -3.636733 4.450167 0.382771

H -4.046787 3.743315 1.954140

H -1.563406 0.951635 1.928980

H -1.474711 2.484863 2.852361

H -3.002900 1.605351 2.747744

H -3.863723 2.155101 -0.794917

H -3.096092 0.728143 -0.040983

H -4.404690 1.589368 0.786426

H -2.357197 1.404650 -4.407897

H -3.840762 1.462238 -6.389345

N -5.795808 -0.271302 -6.904129

H -5.838357 -2.109160 -4.975322

H -4.341378 -2.145833 -2.997613

H -1.333813 -0.965323 -0.280195

H -5.587422 0.346826 -7.678192

H -6.379828 -1.069892 -7.118118

**^NH2^TS** (1/5) for 2,4-DTBP

Mn 5.631930 3.750746 4.652104

O 6.777842 0.631368 7.022491

O 3.523162 1.247144 4.176573

O 4.197257 3.227944 3.368084

O 5.306949 2.169710 5.812237

N 6.123931 5.413644 3.457448

N 7.856636 3.150395 3.723671

N 7.079611 4.583700 5.965595

N 4.454311 5.649377 5.646616

C 8.258640 -2.020804 7.270728

C 7.106899 -3.308440 9.067342

C 5.732951 -2.229589 7.288368

C 6.996892 -2.062814 8.167207

C 6.690337 1.628075 13.418020

C 8.151500 -0.385198 13.163306

C 5.639309 -0.627674 13.172878

C 6.819359 0.263760 12.716378

C 6.800968 0.394937 11.183925

C 6.905557 -0.762894 10.376674

C 6.891981 -0.759089 8.982377

C 6.681378 1.630503 10.519206

C 6.776770 0.524793 8.316401

C 6.673028 1.691540 9.133866

C 4.071441 6.338557 4.386357

C 5.221747 6.407518 3.399029

C 5.382413 7.447520 2.481881

C 6.496936 7.426877 1.638008

C 7.450222 6.414654 1.766013

C 7.244420 5.414728 2.721861

C 9.365718 1.641441 2.334881

C 7.059640 2.277552 1.535489

C 7.344640 0.691751 3.443132

C 7.917583 1.952694 2.770775

C 8.322397 4.405104 3.075292

C 8.564843 2.911586 5.007376

C 8.288529 3.999631 6.025151

C 9.223419 4.412774 6.976361

C 8.874426 5.443830 7.853092

C 7.638061 6.079324 7.722279

C 6.756652 5.636609 6.731165

C 5.505773 6.414697 6.365825

C 2.471821 6.600734 6.917719

C 3.774670 4.639193 7.826332

C 3.257941 5.325616 6.548660

C 2.305204 4.340068 5.851357

C 2.452000 3.035181 1.174444

C 1.571043 2.917777 0.107494

C 0.720915 1.789641 -0.008921

C 0.802773 0.788846 0.992130

C 1.688389 0.918576 2.052424

C 2.531267 2.041382 2.169399

C 3.467108 2.153253 3.317974

H 9.170425 -1.934775 7.884505

H 8.336336 -2.950283 6.682299

H 8.224409 -1.170286 6.578240

H 5.962194 1.538975 6.369373

H 7.175097 -4.208324 8.436007

H 8.005452 -3.284204 9.703845

H 6.227661 -3.429785 9.719473

H 5.610207 -1.380191 6.604786

H 5.806342 -3.153289 6.690370

H 4.829720 -2.306513 7.915706

H 5.745021 2.133005 13.164527

H 6.707795 1.486942 14.510072

H 7.520028 2.304109 13.158311

H 9.012240 0.232026 12.859693

H 8.176844 -0.488171 14.260659

H 8.285883 -1.388526 12.731411

H 5.695803 -1.638574 12.741372

H 5.642423 -0.732836 14.270307

H 4.674218 -0.186652 12.876123

H 6.585347 2.654976 8.626728

H 6.997522 -1.720991 10.886086

H 6.596210 2.559891 11.083022

H 3.252128 5.775157 3.918845

H 3.702139 7.362751 4.563061

H 4.655801 8.260348 2.437014

H 6.637733 8.219397 0.899771

H 8.351564 6.409967 1.150659

H 9.845390 2.477992 1.805395

H 10.002234 1.363248 3.188283

H 9.357141 0.786319 1.642761

H 6.025918 2.517111 1.822730

H 7.460685 3.116271 0.948016

H 7.036483 1.402547 0.869513

H 7.974304 0.326184 4.266143

H 6.329828 0.841773 3.831541

H 7.300437 -0.111760 2.693442

H 9.005007 4.932289 3.762443

H 8.928673 4.203001 2.182879

H 8.211241 1.961034 5.432920

H 9.656885 2.823550 4.877357

H 10.206832 3.942175 7.021132

H 9.581009 5.774448 8.617524

H 7.368474 6.923567 8.359272

H 5.854736 7.237310 5.720022

H 5.111291 6.912870 7.261096

H 3.103192 7.366689 7.393276

H 1.983445 7.053195 6.041633

H 1.679004 6.343223 7.635563

H 4.363558 3.744210 7.580185

H 4.380261 5.303031 8.459450

H 2.913405 4.319929 8.431333

H 1.894054 4.726349 4.908419

H 2.788929 3.374775 5.657180

H 1.450633 4.152848 6.518229

H 3.100526 3.909985 1.247659

H 1.526934 3.700514 -0.654618

N -0.175058 1.685539 -1.035914

H 0.157871 -0.091224 0.921142

H 1.743163 0.141121 2.816884

H 4.608612 1.635507 5.318548

H -0.081772 2.296066 -1.838206

H -0.623252 0.795107 -1.213366

**^NH2^IM** (1/5) for 2,4-DTBP

Mn -0.021457 1.166859 -1.057173

O 1.138913 -1.857456 1.915087

O -2.571486 -1.066831 -0.777754

O -1.788911 0.600807 -2.056627

O -0.508599 -0.216903 0.512380

N 0.621477 2.564536 -2.628495

N 2.009505 0.080225 -2.148533

N 1.814786 1.990049 -0.118261

N -0.643346 3.457921 -0.316649

C 3.922203 -2.288954 3.132095

C 3.855213 -4.536047 4.216963

C 2.990384 -4.296386 1.887586

C 3.096070 -3.593233 3.264175

C -1.946204 -3.713326 7.240829

C 0.458268 -3.663515 7.940337

C -0.470321 -5.663534 6.700962

C -0.519809 -4.120560 6.829393

C -0.094880 -3.492373 5.497038

C 1.199396 -3.770592 4.973241

C 1.689055 -3.261250 3.782839

C -0.942746 -2.633700 4.751558

C 0.798678 -2.373797 3.015064

C -0.516465 -2.095116 3.561237

C -1.078950 3.989691 -1.635580

C -0.073634 3.705650 -2.740205

C 0.156499 4.575377 -3.808880

C 1.128071 4.224062 -4.752319

C 1.883563 3.063496 -4.574781

C 1.617822 2.254081 -3.462459

C 3.043502 -1.866382 -3.430687

C 0.728580 -1.027783 -3.968289

C 1.160659 -2.273624 -1.847965

C 1.751914 -1.255931 -2.841627

C 2.543298 1.105042 -3.082721

C 2.861352 -0.044780 -0.938281

C 2.924712 1.237732 -0.127510

C 4.069208 1.647623 0.561555

C 4.027447 2.860360 1.257036

C 2.885081 3.660035 1.189601

C 1.787069 3.199208 0.452091

C 0.614002 4.113396 0.127280

C -2.223134 4.961679 0.989428

C -1.174651 2.931186 2.061710

C -1.735104 3.516948 0.751881

C -2.938829 2.649891 0.342930

C -3.898583 0.280697 -3.903868

C -4.954960 0.102641 -4.789867

C -5.936677 -0.890293 -4.555306

C -5.803274 -1.694448 -3.397155

C -4.741615 -1.505062 -2.520849

C -3.767296 -0.516007 -2.751594

C -2.637503 -0.325021 -1.790612

H 4.026907 -1.792153 4.110216

H 4.934121 -2.526561 2.765723

H 3.456471 -1.583418 2.432525

H 0.066567 -0.845248 1.016067

H 4.853451 -4.742643 3.801504

H 3.999125 -4.091704 5.214196

H 3.343139 -5.503136 4.339874

H 2.492553 -3.658303 1.146636

H 3.999052 -4.541010 1.517032

H 2.425272 -5.238638 1.972149

H -2.696177 -4.035861 6.502103

H -2.201116 -4.189628 8.199820

H -2.041203 -2.624901 7.377131

H 0.443238 -2.568116 8.054526

H 0.162671 -4.110607 8.902907

H 1.494434 -3.970648 7.734326

H 0.539371 -6.028806 6.461148

H -0.774931 -6.125817 7.653465

H -1.156776 -6.017217 5.915604

H -1.159404 -1.431054 2.979708

H 1.840096 -4.429796 5.555641

H -1.941070 -2.391358 5.114167

H -2.028211 3.504843 -1.904148

H -1.268883 5.076682 -1.607496

H -0.402783 5.508572 -3.896817

H 1.317918 4.874254 -5.609473

H 2.681594 2.801138 -5.272252

H 3.491318 -1.245047 -4.220503

H 3.806368 -2.042138 -2.657092

H 2.808755 -2.841094 -3.884258

H -0.219441 -0.636025 -3.572710

H 1.096298 -0.333971 -4.738619

H 0.521639 -1.984492 -4.470642

H 1.905157 -2.639238 -1.126242

H 0.310101 -1.866586 -1.286936

H 0.804511 -3.149657 -2.409582

H 3.425069 1.582693 -2.623176

H 2.928604 0.648653 -4.004091

H 2.437988 -0.829693 -0.295078

H 3.893503 -0.355093 -1.179885

H 4.973874 1.037284 0.545891

H 4.899500 3.197534 1.822077

H 2.853711 4.636375 1.677333

H 0.977847 4.768010 -0.682191

H 0.442407 4.792794 0.973157

H -1.406140 5.644556 1.268264

H -2.729725 5.376215 0.104845

H -2.950064 4.971204 1.815296

H -0.822017 1.900757 1.910490

H -0.356320 3.529366 2.486935

H -1.977146 2.906307 2.813878

H -3.422004 2.989587 -0.583845

H -2.659662 1.594653 0.227173

H -3.697158 2.708421 1.137928

H -3.149374 1.050109 -4.099321

H -5.036100 0.733456 -5.679505

N -7.006620 -1.040312 -5.401844

H -6.547838 -2.469857 -3.196215

H -4.648581 -2.130489 -1.630714

H -1.317769 -0.692312 0.132879

H -6.948271 -0.632922 -6.327655

H -7.545805 -1.896501 -5.349958

**^NO2^RC** (1/5) for 2,4-DTBP

Mn 0.065999 0.966784 -0.660978

O 1.019610 -2.393494 1.658097

O -2.285593 -1.281400 -1.271535

O -1.263969 0.574602 -2.010342

O -0.355724 -0.435033 0.437385

N 0.673799 2.550365 -1.820016

N 2.189691 0.168694 -1.550508

N 1.477957 1.633201 0.670984

N -1.037477 2.909050 0.305450

C 3.345674 -4.315286 2.001536

C 2.767005 -5.785277 3.923810

C 1.055199 -5.391622 2.159534

C 2.200947 -4.724977 2.959045

C 0.755446 -1.035031 8.023235

C 2.817977 -2.429630 7.815007

C 0.551057 -3.524248 7.956580

C 1.334332 -2.315270 7.393834

C 1.230962 -2.300411 5.855137

C 1.712142 -3.392615 5.105991

C 1.664818 -3.482717 3.706668

C 0.670470 -1.240906 5.132682

C 1.091825 -2.380112 3.014608

C 0.605233 -1.282886 3.737677

C -1.326739 3.640537 -0.952886

C -0.151057 3.611600 -1.905753

C 0.107104 4.629724 -2.823731

C 1.232901 4.524714 -3.644180

C 2.103885 3.444521 -3.492355

C 1.807204 2.467246 -2.539709

C 3.569600 -1.533139 -2.833523

C 1.457737 -0.573920 -3.818528

C 1.334350 -2.175175 -1.912131

C 2.149260 -1.020338 -2.517883

C 2.791964 1.378725 -2.168884

C 2.851865 -0.149825 -0.261002

C 2.620918 0.930597 0.774081

C 3.535165 1.226582 1.785809

C 3.238829 2.261373 2.676361

C 2.075968 3.014731 2.504530

C 1.209883 2.688054 1.458842

C 0.047399 3.578440 1.068185

C -2.985387 3.997971 1.513716

C -1.867789 1.937552 2.451634

C -2.286250 2.668223 1.163806

C -3.281412 1.760534 0.422891

C -2.885874 0.698950 -4.273935

C -3.714763 0.735203 -5.393974

C -4.681303 -0.261379 -5.541610

C -4.843939 -1.288090 -4.608574

C -4.008901 -1.311674 -3.494175

C -3.027661 -0.322013 -3.320424

C -2.145397 -0.378630 -2.100285

H 4.183004 -3.870784 2.564226

H 3.731477 -5.201018 1.469665

H 3.008338 -3.586283 1.253883

H 0.502741 -1.620724 1.307381

H 3.131994 -6.646990 3.342989

H 3.615113 -5.401962 4.512570

H 2.004498 -6.159833 4.624484

H 0.613881 -4.699120 1.431514

H 1.434040 -6.272708 1.614970

H 0.257137 -5.734378 2.838355

H -0.312687 -0.907694 7.786664

H 0.848437 -1.083375 9.119784

H 1.289574 -0.133148 7.684838

H 3.401575 -1.573704 7.439055

H 2.907935 -2.445773 8.913957

H 3.283893 -3.349615 7.429721

H 0.943948 -4.480084 7.577194

H 0.617792 -3.549978 9.057008

H -0.514603 -3.465545 7.682080

H 0.167698 -0.448336 3.185993

H 2.147708 -4.226755 5.653939

H 0.273940 -0.360927 5.640270

H -2.182180 3.160403 -1.447668

H -1.606437 4.692192 -0.774080

H -0.558502 5.491779 -2.885771

H 1.446439 5.301053 -4.381880

H 3.014255 3.366827 -4.088600

H 4.224125 -0.749611 -3.244613

H 4.059647 -1.962621 -1.947177

H 3.505797 -2.330656 -3.588549

H 0.464946 -0.149328 -3.615889

H 2.044850 0.161734 -4.386471

H 1.325859 -1.448942 -4.471311

H 1.786080 -2.587105 -0.999509

H 0.305224 -1.877459 -1.677147

H 1.289649 -2.992945 -2.646246

H 3.490007 1.840333 -1.451067

H 3.411035 1.131638 -3.041058

H 2.432861 -1.086390 0.131605

H 3.938526 -0.300171 -0.372141

H 4.464649 0.660999 1.865845

H 3.930645 2.500732 3.486730

H 1.850432 3.860430 3.156134

H 0.484236 4.377587 0.446812

H -0.333314 4.092950 1.959767

H -2.313180 4.713889 2.010904

H -3.413695 4.487314 0.626212

H -3.815403 3.797639 2.207207

H -1.325961 1.012772 2.209352

H -1.249340 2.555159 3.118147

H -2.771752 1.665928 3.015914

H -3.624364 2.178190 -0.534134

H -2.862830 0.761872 0.248364

H -4.174812 1.637230 1.052410

H -2.121468 1.463063 -4.131763

H -3.623109 1.517099 -6.146521

N -5.562511 -0.228734 -6.725658

H -5.611388 -2.045191 -4.763484

H -4.107274 -2.097222 -2.744067

H -1.101892 -0.924152 0.010090

O -5.405474 0.677262 -7.530010

O -6.401306 -1.109679 -6.837743

**^NO2^TS** (1/5) for 2,4-DTBP

Mn 5.643301 3.738053 4.655773

O 6.765594 0.633585 7.013472

O 3.490980 1.239351 4.157282

O 4.189565 3.217088 3.360038

O 5.330764 2.162249 5.811655

N 6.121370 5.394188 3.457330

N 7.860639 3.137730 3.724271

N 7.087432 4.572534 5.965131

N 4.457718 5.629028 5.649785

C 8.282081 -1.994603 7.271371

C 7.144542 -3.293353 9.068687

C 5.759156 -2.239709 7.283809

C 7.018713 -2.051921 8.164734

C 6.647142 1.644212 13.407381

C 8.139270 -0.347226 13.158833

C 5.630836 -0.627911 13.164826

C 6.798031 0.280566 12.708866

C 6.780207 0.408309 11.176221

C 6.904399 -0.749114 10.371721

C 6.892961 -0.748036 8.977113

C 6.642346 1.640494 10.509115

C 6.759694 0.532095 8.312041

C 6.636145 1.698615 9.123468

C 4.069918 6.318294 4.390605

C 5.216795 6.386715 3.399830

C 5.373383 7.425551 2.480959

C 6.486126 7.406561 1.634798

C 7.442320 6.397155 1.762531

C 7.241220 5.397761 2.719626

C 9.372772 1.630568 2.336331

C 7.065794 2.259823 1.536509

C 7.355157 0.676444 3.446367

C 7.923999 1.938358 2.772367

C 8.322646 4.392722 3.072768

C 8.574577 2.902595 5.005720

C 8.297113 3.989967 6.023940

C 9.231151 4.404446 6.975227

C 8.880192 5.434590 7.852275

C 7.642594 6.067977 7.722263

C 6.761762 5.624162 6.731355

C 5.508879 6.398661 6.366087

C 2.474634 6.580728 6.920442

C 3.782970 4.625588 7.835092

C 3.263058 5.305942 6.555673

C 2.312054 4.315213 5.863758

C 2.460847 3.058957 1.170358

C 1.580047 2.952993 0.095319

C 0.756760 1.827989 0.015270

C 0.788081 0.811176 0.972570

C 1.673649 0.931104 2.041029

C 2.513332 2.051672 2.147050

C 3.461281 2.153095 3.317747

H 9.190757 -1.891327 7.887009

H 8.375507 -2.925922 6.688288

H 8.237454 -1.148933 6.573588

H 5.999754 1.512662 6.395386

H 7.226705 -4.193725 8.439821

H 8.041472 -3.254751 9.706697

H 6.265792 -3.425408 9.719332

H 5.625304 -1.395655 6.595705

H 5.847183 -3.164793 6.690059

H 4.855727 -2.326881 7.909443

H 5.694562 2.134079 13.151578

H 6.665150 1.505493 14.499698

H 7.466866 2.332245 13.147563

H 8.991007 0.282191 12.854923

H 8.164476 -0.447021 14.256436

H 8.289308 -1.349533 12.729739

H 5.703620 -1.638753 12.735670

H 5.633711 -0.730610 14.262443

H 4.659676 -0.202273 12.865477

H 6.536072 2.660010 8.615331

H 7.010405 -1.704689 10.882839

H 6.541773 2.569624 11.070605

H 3.247542 5.756672 3.926287

H 3.702203 7.342598 4.568989

H 4.644895 8.236686 2.436873

H 6.623501 8.198480 0.895324

H 8.342789 6.394157 1.145969

H 9.850449 2.468092 1.806683

H 10.010043 1.353560 3.189469

H 9.365669 0.775491 1.644201

H 6.031420 2.498370 1.821843

H 7.465248 3.098006 0.947342

H 7.043773 1.383590 0.872233

H 7.987606 0.312730 4.268049

H 6.340969 0.823407 3.837733

H 7.311610 -0.127496 2.697183

H 9.004582 4.923126 3.758055

H 8.928337 4.190455 2.180117

H 8.227406 1.950129 5.431968

H 9.666328 2.819733 4.871360

H 10.215363 3.935553 7.019872

H 9.586307 5.766227 8.616664

H 7.371650 6.911366 8.359758

H 5.855048 7.220596 5.718010

H 5.114083 6.897650 7.260603

H 3.105378 7.350959 7.389765

H 1.981648 7.027169 6.043904

H 1.685277 6.324478 7.642471

H 4.373634 3.731140 7.591447

H 4.387502 5.292940 8.465425

H 2.923168 4.306076 8.441948

H 1.902453 4.693618 4.916962

H 2.796697 3.348202 5.680185

H 1.456177 4.132120 6.529958

H 3.117121 3.924832 1.260425

H 1.522994 3.721883 -0.673961

N -0.178302 1.709035 -1.120101

H 0.128272 -0.049428 0.871170

H 1.725767 0.157451 2.807855

H 4.627870 1.623446 5.351553

O -0.195442 2.611337 -1.943868

O -0.886286 0.714864 -1.176180

**^NO2^IM** (1/5) for 2,4-DTBP

Mn 0.138768 1.099903 -0.736376

O 1.203846 -2.265183 1.905226

O -2.391301 -1.216172 -0.936677

O -1.562967 0.622705 -1.923247

O -0.380990 -0.522297 0.580706

N 0.845992 2.639064 -2.143744

N 2.294069 0.166859 -1.780718

N 1.873065 1.874813 0.396847

N -0.602494 3.310186 0.135751

C 4.027972 -3.206765 2.662167

C 3.711103 -5.198848 4.132698

C 2.434948 -5.064795 1.990877

C 2.987070 -4.232896 3.175821

C -0.962973 -2.546806 7.951280

C 1.494405 -2.977414 8.174358

C -0.078050 -4.854083 7.540290

C 0.220430 -3.343252 7.372512

C 0.457983 -3.040205 5.888652

C 1.520996 -3.693279 5.202823

C 1.832236 -3.494281 3.868735

C -0.344265 -2.128264 5.155935

C 1.000850 -2.535160 3.121623

C -0.087993 -1.886134 3.827670

C -0.963029 3.938011 -1.163084

C 0.118559 3.763281 -2.217195

C 0.385546 4.720268 -3.199494

C 1.429679 4.476093 -4.097491

C 2.217589 3.332489 -3.952955

C 1.910199 2.431165 -2.925807

C 3.510668 -1.622605 -3.124576

C 1.244545 -0.772249 -3.829253

C 1.474682 -2.218507 -1.812980

C 2.146677 -1.096302 -2.624908

C 2.859068 1.297976 -2.558915

C 3.044457 -0.052877 -0.518335

C 3.001763 1.149608 0.409395

C 4.077661 1.522012 1.219533

C 3.950154 2.669195 2.009118

C 2.796248 3.449829 1.917582

C 1.770276 3.031818 1.060951

C 0.606008 3.950434 0.715485

C -2.329059 4.667656 1.413373

C -1.273501 2.622026 2.440931

C -1.765153 3.259878 1.127563

C -2.897012 2.370057 0.584562

C -3.542313 0.558881 -3.905546

C -4.553205 0.509390 -4.863785

C -5.511498 -0.502779 -4.771898

C -5.485535 -1.461869 -3.756218

C -4.466947 -1.398850 -2.807838

C -3.491087 -0.391374 -2.873365

C -2.398824 -0.335560 -1.823289

H 4.442609 -2.618462 3.496635

H 4.863206 -3.736974 2.176532

H 3.587142 -2.514575 1.933999

H 0.176886 -1.193425 1.051097

H 4.524020 -5.704240 3.589119

H 4.163964 -4.674874 4.988853

H 3.039232 -5.980011 4.521230

H 1.943909 -4.426858 1.245345

H 3.260926 -5.604012 1.499420

H 1.707341 -5.813520 2.343654

H -1.909232 -2.785529 7.441582

H -1.088609 -2.798309 9.015455

H -0.799512 -1.459892 7.885841

H 1.731721 -1.906601 8.073312

H 1.335259 -3.191923 9.243188

H 2.372044 -3.553799 7.845841

H 0.755622 -5.483382 7.194672

H -0.250140 -5.082905 8.604145

H -0.980304 -5.143876 6.979040

H -0.698487 -1.187820 3.251375

H 2.123931 -4.395428 5.775130

H -1.172288 -1.609799 5.637993

H -1.883349 3.462897 -1.531315

H -1.176564 5.016096 -1.061938

H -0.201827 5.638597 -3.254075

H 1.650209 5.196222 -4.888818

H 3.071851 3.152847 -4.608665

H 4.040226 -0.901694 -3.765149

H 4.176217 -1.895876 -2.291774

H 3.351569 -2.530452 -3.725596

H 0.258074 -0.415052 -3.501162

H 1.682443 -0.017275 -4.498137

H 1.095634 -1.683332 -4.427607

H 2.117136 -2.605267 -1.009285

H 0.523922 -1.899223 -1.368626

H 1.264072 -3.061922 -2.486878

H 3.659082 1.773686 -1.967085

H 3.362665 0.951819 -3.471574

H 2.594010 -0.906459 0.008891

H 4.102869 -0.313563 -0.696592

H 4.997494 0.934518 1.223369

H 4.766968 2.974126 2.667312

H 2.702798 4.381409 2.479191

H 1.015435 4.663479 -0.019200

H 0.360510 4.567877 1.590270

H -1.556615 5.366341 1.769821

H -2.807488 5.108495 0.525959

H -3.096592 4.603130 2.199039

H -0.843319 1.625845 2.263077

H -0.531848 3.237517 2.969211

H -2.130132 2.500329 3.120171

H -3.312832 2.729895 -0.366689

H -2.567887 1.330529 0.452835

H -3.721117 2.366814 1.313425

H -2.779520 1.336881 -3.947171

H -4.610515 1.236825 -5.672474

N -6.586283 -0.559826 -5.779140

H -6.252686 -2.234309 -3.721046

H -4.414319 -2.129363 -1.999832

H -1.156949 -0.965208 0.126285

O -6.588472 0.283950 -6.663597

O -7.420259 -1.447740 -5.677998

**^H^RC** (1/5) for 4-MeO-2,6-DTBP

Mn -0.084811 1.012148 -1.189082

H -0.352984 -5.373415 4.029744

O -1.693119 -1.794817 -1.855400

O -1.266323 0.296548 -2.538580

O -0.119171 -0.446524 -0.103184

N 0.085308 2.705654 -2.358514

N 2.177399 0.804994 -2.107097

N 1.122528 2.048949 0.122827

N -1.634491 2.620593 -0.215702

C -1.760222 -2.804566 1.639577

C 0.417099 -3.732302 0.641236

C -1.183223 -5.202803 1.815648

C -0.575320 -3.784398 1.830115

C 2.856231 -0.626513 6.319708

C 1.259862 0.542285 4.820547

C 3.400836 -0.477780 3.901789

C 2.237248 -0.654889 4.906744

C 1.490252 -1.983852 4.628503

C 0.827763 -2.237689 3.388546

C 0.157468 -3.466925 3.157539

C 1.454774 -2.978943 5.607866

C 0.156937 -4.428491 4.188663

C 0.796242 -4.195851 5.404177

C -2.124820 3.228229 -1.477461

C -0.989982 3.512276 -2.438857

C -1.013657 4.565030 -3.353880

C 0.097262 4.762870 -4.177937

C 1.222337 3.949909 -4.032341

C 1.196590 2.925883 -3.082047

C 3.953464 -0.453001 -3.417418

C 1.644954 -0.106630 -4.366384

C 1.993826 -1.681845 -2.466140

C 2.451923 -0.347537 -3.077705

C 2.435633 2.132266 -2.722033

C 2.892421 0.665890 -0.813988

C 2.415165 1.682300 0.200727

C 3.252524 2.258867 1.155304

C 2.722066 3.218699 2.021559

C 1.394365 3.622989 1.878708

C 0.611236 3.028567 0.885404

C -0.758161 3.566062 0.518951

C -3.795740 3.146059 1.003624

C -2.134128 1.508659 1.962979

C -2.754718 2.059382 0.666035

C -3.458458 0.893170 -0.046518

C -2.926704 0.026407 -4.768949

C -3.769674 -0.145329 -5.869481

C -4.437314 -1.360951 -6.058303

C -4.260709 -2.407039 -5.144180

C -3.418647 -2.237545 -4.044197

C -2.745789 -1.019583 -3.848602

C -1.849365 -0.865418 -2.655033

H -1.468186 -1.746917 1.667011

H -2.252705 -2.979609 0.670250

H -2.504944 -2.957169 2.436776

H -0.112416 -3.926557 -0.304859

H 0.931296 -2.769289 0.533559

H 1.191248 -4.506582 0.763575

H -1.940441 -5.341535 2.602169

H -1.682274 -5.368311 0.848152

H -0.417323 -5.985176 1.929718

H 3.600157 -1.424892 6.466266

H 3.372547 0.334961 6.467675

H 2.095375 -0.716588 7.110557

H 0.455757 0.438267 5.567615

H 1.795168 1.482286 5.034831

H 0.801104 0.624912 3.828215

H 3.042420 -0.447438 2.866078

H 3.940845 0.461015 4.109420

H 4.121971 -1.306323 3.996290

H 1.941622 -2.839867 6.571033

H -2.817224 2.523940 -1.958895

H -2.681662 4.164618 -1.306503

H -1.883109 5.221652 -3.410689

H 0.096358 5.570219 -4.913398

H 2.118666 4.116225 -4.632062

H 4.359294 0.474026 -3.849922

H 4.559146 -0.717206 -2.537712

H 4.099205 -1.248376 -4.163325

H 0.576962 0.028105 -4.146759

H 1.998272 0.764818 -4.936012

H 1.750587 -0.981454 -5.024288

H 2.577130 -1.966401 -1.578976

H 0.932645 -1.667333 -2.190656

H 2.140474 -2.477152 -3.211571

H 2.995036 2.759338 -2.008190

H 3.089692 2.059227 -3.600468

H 2.692459 -0.337561 -0.414706

H 3.985171 0.765701 -0.921994

H 4.302057 1.966851 1.209575

H 3.353695 3.673510 2.787518

H 0.973026 4.407557 2.509264

H -0.567308 4.448069 -0.114534

H -1.245714 3.968050 1.416557

H -3.343458 4.034322 1.471108

H -4.357240 3.472322 0.115390

H -4.525371 2.738631 1.719053

H -1.324946 0.799749 1.739799

H -1.747009 2.297367 2.623483

H -2.910190 0.972885 2.528792

H -3.919990 1.183043 -1.001054

H -2.768580 0.058810 -0.221664

H -4.269179 0.525595 0.599547

H -2.401224 0.969773 -4.614202

H -3.907003 0.670965 -6.582844

H -5.096726 -1.493950 -6.919809

H -4.781586 -3.356372 -5.291119

H -3.270255 -3.042502 -3.322461

H -0.662699 -1.125374 -0.574458

O 0.909024 -1.229732 2.466487

O 0.835631 -5.082994 6.443770

C 0.190009 -6.330702 6.304172

H 0.346181 -6.873798 7.246052

H 0.615077 -6.925552 5.475779

H -0.895852 -6.217198 6.134568

H 0.384258 -1.323325 1.652233

**^H^TS** (1/5) for 4-MeO-2,6-DTBP

Mn 5.675068 3.884156 4.806556

H 5.345019 -2.647589 9.767740

O 4.014215 1.077653 4.174227

O 4.483544 3.128496 3.413977

O 5.545247 2.325876 5.995219

N 5.895495 5.575286 3.558632

N 7.974369 3.669956 3.923901

N 6.895942 5.023052 6.100406

N 4.130719 5.567335 5.682504

C 3.988433 -0.543103 7.105578

C 6.177536 -1.424144 6.123742

C 4.722783 -2.885877 7.488406

C 5.229887 -1.434856 7.346434

C 8.451359 2.420964 11.452470

C 7.014822 3.295959 9.626227

C 9.207682 2.114109 9.105079

C 7.958831 2.136429 10.018683

C 7.207279 0.791044 9.915237

C 6.632732 0.342538 8.661160

C 5.940260 -0.925814 8.621382

C 7.091056 -0.020728 11.037405

C 5.860463 -1.692287 9.790182

C 6.428565 -1.257299 10.992788

C 3.675159 6.133813 4.386966

C 4.826922 6.380100 3.433325

C 4.816820 7.393595 2.473303

C 5.935856 7.545709 1.650066

C 7.053135 6.729995 1.838025

C 7.011173 5.751010 2.835207

C 9.778478 2.343631 2.721756

C 7.490439 2.642248 1.704432

C 7.792859 1.169544 3.687989

C 8.270038 2.469320 3.020840

C 8.241497 4.962620 3.240476

C 8.672768 3.607504 5.235099

C 8.202976 4.706617 6.165644

C 9.056299 5.406960 7.017349

C 8.525259 6.439563 7.797017

C 7.181422 6.786550 7.664283

C 6.383233 6.065636 6.768452

C 4.988812 6.542452 6.403314

C 1.952483 6.169347 6.841363

C 3.554698 4.516712 7.873902

C 2.977129 5.053340 6.551946

C 2.260259 3.886304 5.852676

C 2.922032 2.756042 1.121538

C 2.126011 2.534295 -0.004766

C 1.453479 1.316479 -0.159072

C 1.578721 0.318863 0.815589

C 2.374258 0.538786 1.941346

C 3.051715 1.758988 2.102664

C 3.900397 1.973358 3.323873

H 4.243722 0.522607 7.072295

H 3.499335 -0.798877 6.153084

H 3.258547 -0.683480 7.918934

H 5.643736 -1.793728 5.233371

H 6.566303 -0.424671 5.910125

H 7.037384 -2.091495 6.300453

H 3.964955 -2.995949 8.278818

H 4.248824 -3.192881 6.543024

H 5.542361 -3.592587 7.693831

H 9.167203 1.663315 11.807193

H 8.967928 3.393481 11.469749

H 7.622144 2.475426 12.175132

H 6.140285 3.326605 10.296488

H 7.539461 4.260422 9.716402

H 6.655673 3.192257 8.596432

H 8.932886 1.934444 8.059034

H 9.745081 3.074469 9.171060

H 9.902627 1.319060 9.421694

H 7.514633 0.269526 11.997040

H 2.989051 5.417264 3.915005

H 3.119285 7.077651 4.515381

H 3.952495 8.052855 2.378697

H 5.946678 8.319382 0.879208

H 7.953744 6.861141 1.235671

H 10.200241 3.253740 2.268988

H 10.360489 2.106834 3.624953

H 9.938437 1.522538 2.007292

H 6.419529 2.796675 1.896696

H 7.861206 3.477002 1.092718

H 7.600481 1.730001 1.100079

H 8.326744 0.946665 4.622072

H 6.716343 1.184848 3.898710

H 7.986980 0.331478 3.002651

H 8.806817 5.621045 3.920351

H 8.895633 4.838335 2.367971

H 8.448770 2.637495 5.701696

H 9.768348 3.677279 5.133941

H 10.117279 5.156919 7.057965

H 9.169119 6.994283 8.482906

H 6.758282 7.624060 8.221751

H 5.141586 7.429524 5.766403

H 4.496035 6.932185 7.303327

H 2.411882 7.055794 7.304975

H 1.423412 6.491674 5.932066

H 1.193928 5.792299 7.543227

H 4.324919 3.756465 7.684790

H 3.979483 5.305062 8.511199

H 2.745708 4.043575 8.449490

H 1.812844 4.166072 4.888677

H 2.932824 3.033489 5.698091

H 1.437185 3.545145 6.497623

H 3.451244 3.701225 1.250472

H 2.029184 3.313060 -0.765266

H 0.830606 1.144013 -1.040578

H 1.054101 -0.632300 0.695942

H 2.481846 -0.228705 2.709558

H 4.979234 1.693492 5.473807

O 6.836234 1.067146 7.578261

O 6.390321 -1.952926 12.156592

C 5.745285 -3.213009 12.200007

H 5.844882 -3.578494 13.230259

H 6.218589 -3.936830 11.514507

H 4.673532 -3.131204 11.949821

H 6.126898 1.683841 6.829521

**^H^PC** (1/5) for 4-MeO-2,6-DTBP

Mn -0.124011 1.196142 -1.021728

H -0.187531 -5.929886 4.122694

O -1.950864 -1.679901 -1.647763

O -1.445918 0.343750 -2.468835

O -0.594594 -0.510390 0.203490

N 0.143668 2.921430 -2.380609

N 2.294033 1.068604 -1.882499

N 1.156730 2.502683 0.230156

N -1.644098 3.005794 -0.236650

C -2.065207 -3.476331 1.856974

C -0.000032 -4.317029 0.627188

C -1.363827 -5.858777 2.025943

C -0.834292 -4.414309 1.928981

C 2.941710 -0.830878 5.828086

C 1.040129 0.128315 4.533740

C 3.105000 -0.748625 3.342203

C 2.130584 -0.971531 4.525166

C 1.476515 -2.360436 4.407956

C 0.650611 -2.696107 3.228546

C 0.036401 -4.035939 3.141381

C 1.642215 -3.314526 5.384863

C 0.254745 -4.938723 4.168227

C 1.046622 -4.597486 5.285933

C -2.074226 3.507011 -1.569150

C -0.912204 3.735308 -2.520738

C -0.911494 4.741856 -3.490073

C 0.214155 4.873433 -4.309587

C 1.323943 4.052054 -4.102826

C 1.263821 3.086310 -3.089745

C 4.122867 -0.246805 -3.067588

C 1.767205 -0.179396 -3.966177

C 2.285162 -1.454983 -1.888064

C 2.626740 -0.184041 -2.688951

C 2.504849 2.316207 -2.660809

C 3.002082 1.135109 -0.580584

C 2.468455 2.233179 0.322546

C 3.288280 2.961841 1.187861

C 2.710879 3.979063 1.954183

C 1.362004 4.292517 1.782282

C 0.608696 3.543116 0.869477

C -0.780151 4.001390 0.447507

C -3.798854 3.716564 0.914625

C -2.258331 2.023954 1.974365

C -2.814768 2.558723 0.641242

C -3.585699 1.409853 -0.032205

C -2.821074 -0.188825 -4.863293

C -3.520316 -0.492619 -6.034347

C -4.156097 -1.732455 -6.169402

C -4.090032 -2.667925 -5.129652

C -3.390337 -2.363795 -3.959741

C -2.750001 -1.122044 -3.816483

C -1.996315 -0.806477 -2.546779

H -1.775219 -2.424919 1.752639

H -2.689576 -3.746529 0.990165

H -2.683575 -3.576794 2.763483

H -0.629158 -4.577428 -0.239091

H 0.398187 -3.307986 0.471532

H 0.846174 -5.022448 0.654576

H -2.004912 -6.010056 2.908412

H -1.973563 -6.078774 1.136283

H -0.549611 -6.599540 2.057593

H 3.763840 -1.561118 5.887311

H 3.390126 0.173774 5.868478

H 2.312121 -0.946914 6.724125

H 0.356599 -0.006884 5.387719

H 1.511982 1.119229 4.635274

H 0.448731 0.118185 3.610162

H 2.583247 -0.798361 2.378519

H 3.580864 0.241386 3.432183

H 3.904141 -1.507603 3.346507

H 2.237658 -3.129472 6.277405

H -2.734755 2.753873 -2.020469

H -2.652522 4.444226 -1.495626

H -1.767650 5.410414 -3.597118

H 0.236525 5.637639 -5.089971

H 2.230135 4.172045 -4.699971

H 4.446359 0.615092 -3.670223

H 4.769782 -0.307090 -2.179242

H 4.309563 -1.149150 -3.668940

H 0.696284 -0.117803 -3.724600

H 2.017778 0.650710 -4.642549

H 1.940469 -1.111204 -4.524625

H 2.932370 -1.593428 -1.009968

H 1.239349 -1.473797 -1.553970

H 2.441149 -2.329996 -2.536081

H 3.086273 3.024012 -2.046308

H 3.133526 2.140745 -3.544140

H 2.870369 0.173401 -0.064459

H 4.090064 1.282359 -0.699639

H 4.355860 2.744477 1.252122

H 3.321946 4.551517 2.655745

H 0.905347 5.123678 2.323188

H -0.603794 4.849089 -0.235737

H -1.284992 4.449550 1.313537

H -3.317323 4.579913 1.398457

H -4.288245 4.067924 -0.006182

H -4.592638 3.367556 1.591838

H -1.577033 1.178005 1.806014

H -1.735861 2.791642 2.562677

H -3.094925 1.663345 2.591148

H -4.071413 1.710118 -0.971343

H -2.937664 0.547012 -0.227744

H -4.385330 1.080154 0.647793

H -2.320494 0.773633 -4.746126

H -3.570294 0.238763 -6.845144

H -4.703161 -1.970163 -7.085518

H -4.585582 -3.636714 -5.233117

H -3.328585 -3.081550 -3.140017

H -1.124689 -1.118429 -0.405494

O 0.483697 -1.843331 2.317628

O 1.294599 -5.422309 6.309249

C 0.753466 -6.740361 6.332070

H 1.105937 -7.197271 7.264426

H 1.113313 -7.332709 5.476011

H -0.347785 -6.717921 6.329678

H -0.182941 -1.021151 0.934230

**^S^1** (1/1)

Mn 5.978015 3.792227 4.916828

O 6.099659 2.300541 5.857312

H 5.403737 1.671665 5.530856

O 4.697207 2.985592 3.575585

O 4.343935 0.922689 4.380638

N 4.401145 5.125607 5.774770

N 7.085482 4.767625 6.215021

N 7.981264 3.590784 4.040908

N 5.963832 5.373737 3.659312

C 4.157529 1.822880 3.535032

C 3.233079 1.558458 2.371697

C 2.596021 0.311165 2.266373

H 2.789155 -0.440230 3.033986

C 1.734789 0.044720 1.199785

H 1.243164 -0.928680 1.125415

C 1.501263 1.024085 0.226733

H 0.827254 0.816144 -0.608395

C 2.132275 2.269778 0.324720

H 1.951418 3.035553 -0.433961

C 2.993923 2.536163 1.392082

H 3.491547 3.503466 1.477114

C 2.672893 3.276278 6.071802

H 1.896543 2.909484 6.759198

H 3.373889 2.454359 5.896936

H 2.169595 3.521181 5.126592

C 3.351410 4.485929 6.726430

C 4.063127 3.998700 7.999798

H 3.336043 3.454121 8.619486

H 4.455947 4.819012 8.616416

H 4.882108 3.309552 7.751590

C 2.255797 5.509677 7.094709

H 1.599347 5.057257 7.852041

H 1.624158 5.769218 6.232801

H 2.650807 6.440132 7.527621

C 5.174579 6.189695 6.505279

H 4.599754 6.604998 7.341243

H 5.343036 7.033868 5.818662

C 6.539076 5.735618 6.961386

C 7.280779 6.340223 7.978838

H 6.834885 7.118379 8.600291

C 8.608121 5.942052 8.161619

H 9.210259 6.399034 8.949718

C 9.179382 4.989106 7.310230

H 10.229225 4.706671 7.401768

C 8.375369 4.416226 6.325681

C 8.821372 3.431422 5.275737

H 9.891034 3.568441 5.058878

H 8.676112 2.412354 5.653031

C 8.291139 4.914215 3.394717

H 9.012404 4.805909 2.576642

H 8.784477 5.559461 4.138178

C 8.236169 2.432107 3.032232

C 7.783656 1.078984 3.601994

H 8.045467 0.303269 2.867054

H 6.704605 1.031240 3.774463

H 8.296781 0.817112 4.537538

C 7.441398 2.712480 1.746021

H 7.517549 1.833744 1.089090

H 7.832996 3.568290 1.178072

H 6.379989 2.883133 1.966545

C 9.740589 2.319969 2.698599

H 9.860165 1.579519 1.894499

H 10.332640 1.965359 3.554602

H 10.183198 3.258970 2.335836

C 7.058992 5.653429 2.943988

C 7.027857 6.648751 1.965295

H 7.913709 6.866620 1.366776

C 5.842107 7.368768 1.791063

H 5.788581 8.155024 1.034978

C 4.735655 7.110370 2.608162

H 3.821006 7.699054 2.523552

C 4.833878 6.086845 3.550441

C 3.767823 5.709331 4.549480

H 3.146965 6.587067 4.785225

H 3.121671 4.943432 4.102763

**^T^1** (1/3)

Mn 5.956741 3.826285 4.902889

O 6.045958 2.378132 6.038354

H 5.533989 1.656492 5.621347

O 4.751391 2.983699 3.594025

O 4.439196 0.878555 4.318932

N 4.390464 5.108127 5.784551

N 7.082429 4.813573 6.178684

N 7.983724 3.565058 4.015200

N 5.960662 5.392247 3.664402

C 4.218999 1.804426 3.525917

C 3.269267 1.592949 2.372940

C 2.624787 0.352208 2.235823

H 2.831776 -0.428000 2.970384

C 1.738851 0.128655 1.180212

H 1.240934 -0.839278 1.081361

C 1.488955 1.144071 0.248826

H 0.795796 0.969387 -0.578170

C 2.128122 2.382563 0.377523

H 1.935187 3.175811 -0.349170

C 3.013913 2.606906 1.434834

H 3.518618 3.568097 1.541819

C 2.700681 3.230598 6.064199

H 1.916654 2.849252 6.734826

H 3.435970 2.432905 5.919182

H 2.216227 3.454568 5.103734

C 3.332729 4.462138 6.724426

C 4.033299 4.001379 8.013571

H 3.306457 3.444639 8.623143

H 4.393235 4.835774 8.631993

H 4.868884 3.330644 7.770330

C 2.213700 5.470053 7.058840

H 1.556867 5.016700 7.815314

H 1.587449 5.704972 6.185977

H 2.586952 6.413792 7.483224

C 5.150448 6.187107 6.516635

H 4.574461 6.572644 7.365467

H 5.282200 7.043888 5.838187

C 6.530328 5.762640 6.944891

C 7.274943 6.360278 7.963024

H 6.825851 7.123752 8.599971

C 8.607858 5.972211 8.129041

H 9.213162 6.426284 8.916285

C 9.176393 5.026128 7.269418

H 10.226490 4.743135 7.355307

C 8.368930 4.452229 6.287780

C 8.811183 3.440662 5.261996

H 9.883209 3.561848 5.048051

H 8.649897 2.436597 5.672080

C 8.280330 4.885342 3.344973

H 8.968851 4.757841 2.502036

H 8.813314 5.527765 4.062790

C 8.250917 2.386195 3.039861

C 7.804535 1.053478 3.659007

H 8.018144 0.255656 2.932215

H 6.733972 1.028332 3.884183

H 8.359336 0.804990 4.574352

C 7.456483 2.627797 1.745181

H 7.534795 1.731824 1.112470

H 7.847600 3.468796 1.155040

H 6.394763 2.805743 1.956782

C 9.754845 2.277251 2.707936

H 9.880911 1.505474 1.934782

H 10.353782 1.965515 3.575612

H 10.183441 3.206601 2.305156

C 7.045784 5.644630 2.924385

C 7.003799 6.632053 1.937561

H 7.878756 6.834985 1.318054

C 5.822038 7.363238 1.779651

H 5.763354 8.143594 1.017974

C 4.723528 7.118174 2.611990

H 3.809104 7.707429 2.528955

C 4.828437 6.100988 3.560570

C 3.762242 5.703683 4.554932

H 3.136749 6.573095 4.805338

H 3.117781 4.942153 4.098035

**^Q^1** (1/5)

Mn 5.863918 3.668329 4.885013

O 5.928235 2.252597 5.998305

H 5.385132 1.534028 5.593534

O 4.673741 2.875132 3.583525

O 4.332619 0.794466 4.350124

N 4.281225 5.225407 5.888211

N 7.065617 4.757326 6.167837

N 8.112743 3.538346 3.927165

N 5.945147 5.360125 3.701623

C 4.134802 1.688575 3.520267

C 3.228566 1.456058 2.345543

C 2.603095 0.206115 2.202470

H 2.794313 -0.565453 2.950282

C 1.753892 -0.036028 1.121611

H 1.270170 -1.010175 1.015452

C 1.522410 0.969165 0.174406

H 0.857396 0.779388 -0.672054

C 2.142268 2.216679 0.311183

H 1.961877 3.001287 -0.427760

C 2.992334 2.460723 1.392549

H 3.480896 3.429199 1.508322

C 2.511056 3.443109 6.097787

H 1.728583 3.053042 6.765330

H 3.216379 2.626565 5.900989

H 2.014903 3.723216 5.157796

C 3.203858 4.624431 6.796611

C 3.878386 4.075100 8.067033

H 3.124389 3.545777 8.668122

H 4.299578 4.863155 8.707280

H 4.670847 3.360349 7.802929

C 2.142456 5.678021 7.173952

H 1.438867 5.241018 7.897980

H 1.553784 6.002660 6.302733

H 2.580263 6.571162 7.645614

C 5.142168 6.200464 6.603084

H 4.654430 6.601619 7.501280

H 5.305131 7.077765 5.955594

C 6.527443 5.697298 6.960213

C 7.291733 6.276107 7.976625

H 6.850413 7.028444 8.632416

C 8.627287 5.893282 8.114579

H 9.244182 6.333068 8.901192

C 9.183591 4.975293 7.219952

H 10.238605 4.702371 7.273592

C 8.363400 4.415027 6.239618

C 8.864356 3.432718 5.201457

H 9.948153 3.588055 5.067030

H 8.726115 2.418239 5.598674

C 8.307721 4.868933 3.296393

H 8.946624 4.813468 2.405225

H 8.857387 5.521315 3.995115

C 8.398425 2.385986 2.960238

C 8.015766 1.041319 3.603016

H 8.132362 0.247616 2.850590

H 6.976292 1.029638 3.952546

H 8.667132 0.775182 4.447973

C 7.538760 2.581786 1.698565

H 7.663261 1.709850 1.040045

H 7.832885 3.466193 1.115383

H 6.473806 2.668914 1.953518

C 9.889812 2.333596 2.567463

H 10.042356 1.523183 1.839239

H 10.538206 2.121643 3.430758

H 10.238966 3.263485 2.093535

C 7.033646 5.616629 2.956502

C 7.004979 6.639013 2.004283

H 7.882278 6.835053 1.385693

C 5.850112 7.413397 1.882007

H 5.806414 8.218817 1.145693

C 4.764549 7.181454 2.730882

H 3.873722 7.810234 2.692653

C 4.843187 6.131417 3.646222

C 3.741898 5.812047 4.636424

H 3.156241 6.729036 4.816973

H 3.064266 5.081551 4.173394

**^S^2** (1/1)

Mn 7.374981 13.198247 9.294174

O 6.200913 12.849936 7.876561

O 8.671324 12.361949 8.267958

H 8.213389 12.142993 7.441018

N 8.812465 13.415702 10.687502

N 6.049553 14.264264 10.382841

C 4.833542 12.967197 7.691554

H 4.415619 13.893947 8.138973

H 4.287428 12.137856 8.196960

C 4.421338 12.922596 6.225429

C 5.240552 12.326067 5.256496

H 6.208966 11.925092 5.560614

C 4.828376 12.249610 3.921011

H 5.480703 11.782287 3.177991

C 3.588214 12.768144 3.534464

H 3.266803 12.710429 2.491225

C 2.763356 13.366526 4.494446

H 1.794307 13.780600 4.202654

C 3.179236 13.443521 5.827044

H 2.530109 13.918677 6.569565

N 6.687785 11.737944 10.803630

N 8.130219 15.272070 8.928271

C 8.763862 12.706151 11.820043

C 9.821318 12.745744 12.731182

H 9.786379 12.150577 13.645138

C 10.904760 13.584406 12.454295

H 11.745741 13.638120 13.148955

C 10.889721 14.387899 11.309582

H 11.694967 15.094962 11.104264

C 9.806425 14.277784 10.436871

C 5.096085 13.598812 11.054010

C 4.119439 14.287977 11.772671

H 3.345770 13.744074 12.316766

C 4.172977 15.686016 11.792540

H 3.413782 16.254277 12.334072

C 5.229105 16.352145 11.164559

H 5.327129 17.437206 11.226350

C 6.183643 15.594163 10.482179

C 7.441539 12.031850 12.067655

H 6.848074 12.735111 12.672734

H 7.557274 11.135708 12.688955

C 6.857599 10.238765 10.410231

C 6.168461 9.322593 11.443971

H 6.377605 8.278036 11.171104

H 5.075348 9.442127 11.447889

H 6.537289 9.464307 12.470307

C 9.595261 15.111061 9.201326

H 10.098927 16.082502 9.316244

H 10.038508 14.591647 8.342961

C 7.899304 15.877195 7.513294

C 6.409412 16.211244 7.341501

H 6.234415 16.502959 6.295975

H 6.078082 17.051589 7.967234

H 5.785171 15.337491 7.553694

C 5.245503 12.097975 10.997319

H 4.831866 11.646844 11.912279

H 4.671885 11.698419 10.152400

C 7.499757 16.135720 9.976883

H 7.403742 17.177375 9.646680

H 8.167229 16.167596 10.852533

C 8.357215 9.908788 10.336018

H 8.466661 8.869722 9.992007

H 8.861869 9.974924 11.310343

H 8.856580 10.569701 9.614049

C 6.237755 9.958738 9.033226

H 6.416109 8.901598 8.787953

H 6.687782 10.583204 8.254833

H 5.148461 10.106603 9.016803

C 8.732739 17.164678 7.333534

H 8.428984 17.645657 6.392329

H 9.809994 16.958989 7.259169

H 8.574090 17.899870 8.136821

C 8.294519 14.879028 6.414852

H 8.190869 15.385364 5.443394

H 7.628778 14.009115 6.425066

H 9.336236 14.541286 6.494573

**^T^2** (1/3)

Mn 7.404152 13.217336 9.277206

O 6.229217 13.015378 7.811674

O 8.698904 12.327583 8.314037

H 8.270516 11.985468 7.514268

N 8.783497 13.481446 10.721858

N 6.114344 14.276347 10.420165

C 4.850671 13.012786 7.694598

H 4.384188 13.926321 8.126047

H 4.380028 12.165687 8.242435

C 4.381963 12.894438 6.248792

C 5.245638 12.461003 5.233920

H 6.283444 12.240276 5.488806

C 4.787729 12.319373 3.918051

H 5.475679 11.981139 3.137987

C 3.457770 12.608994 3.598503

H 3.100302 12.500277 2.571147

C 2.587290 13.044008 4.606003

H 1.546238 13.278039 4.366814

C 3.047879 13.186213 5.917657

H 2.361526 13.531362 6.697759

N 6.668263 11.669795 10.799545

N 8.186537 15.374898 8.893932

C 8.747822 12.703633 11.811192

C 9.813070 12.710259 12.714805

H 9.794893 12.066046 13.595355

C 10.885107 13.573650 12.473938

H 11.728667 13.601998 13.167049

C 10.866830 14.420533 11.361334

H 11.675882 15.128954 11.176958

C 9.783279 14.342772 10.485529

C 5.148129 13.607329 11.071575

C 4.155256 14.299933 11.764260

H 3.374863 13.757065 12.299629

C 4.193376 15.698002 11.760722

H 3.419050 16.266206 12.280538

C 5.241557 16.367418 11.124303

H 5.314026 17.455810 11.152020

C 6.220109 15.613187 10.471899

C 7.455534 11.959465 12.043937

H 6.849310 12.601828 12.702555

H 7.639186 11.047082 12.624213

C 6.777095 10.180192 10.399499

C 6.080034 9.271856 11.432880

H 6.237309 8.223278 11.140072

H 4.993214 9.437079 11.471837

H 6.484781 9.385492 12.449804

C 9.623314 15.187227 9.244689

H 10.143115 16.147851 9.388481

H 10.110951 14.664946 8.411533

C 8.017629 15.985382 7.487792

C 6.526023 16.264382 7.240917

H 6.393835 16.582665 6.196654

H 6.125608 17.069675 7.872887

H 5.939118 15.352408 7.394967

C 5.255150 12.100134 11.009739

H 4.827671 11.667825 11.928624

H 4.646805 11.732821 10.173614

C 7.507102 16.202738 9.938668

H 7.340802 17.232818 9.599266

H 8.176171 16.293906 10.809765

C 8.265069 9.804909 10.292821

H 8.340714 8.768998 9.930942

H 8.786683 9.842436 11.260038

H 8.773352 10.466384 9.577795

C 6.124521 9.957119 9.026353

H 6.314873 8.920593 8.712163

H 6.540284 10.634159 8.270298

H 5.032478 10.083369 9.049652

C 8.816214 17.298813 7.353259

H 8.578760 17.758680 6.382588

H 9.903081 17.132429 7.375768

H 8.563018 18.036087 8.130583

C 8.498479 14.992765 6.417617

H 8.359553 15.455693 5.429027

H 7.910720 14.069160 6.459063

H 9.565931 14.747743 6.509325

**^Q^2** (1/5)

Mn 7.393807 13.130101 9.149130

O 6.162230 12.823382 7.802871

O 8.707269 12.240066 8.217636

H 8.312300 11.876639 7.409789

N 8.770421 13.445050 10.671448

N 6.125364 14.218580 10.366779

C 4.787450 12.972607 7.663827

H 4.418065 13.918145 8.113408

H 4.240544 12.160937 8.191008

C 4.340962 12.938909 6.208234

C 5.224339 12.590605 5.178770

H 6.260669 12.363296 5.433275

C 4.785670 12.541265 3.849436

H 5.488148 12.268919 3.056775

C 3.456930 12.838563 3.533598

H 3.114355 12.800904 2.496216

C 2.567157 13.188467 4.557610

H 1.526377 13.426119 4.321213

C 3.007264 13.238629 5.882489

H 2.305811 13.515426 6.676163

N 6.652506 11.553773 10.907412

N 8.208352 15.438199 8.825385

C 8.743112 12.692275 11.783335

C 9.800363 12.745276 12.695894

H 9.782765 12.117985 13.588735

C 10.854414 13.627796 12.456616

H 11.689998 13.687549 13.157354

C 10.817799 14.459662 11.335507

H 11.603111 15.194339 11.151060

C 9.747592 14.341700 10.447060

C 5.184807 13.554175 11.067557

C 4.207076 14.252642 11.776757

H 3.451770 13.708688 12.346018

C 4.226501 15.649068 11.750807

H 3.461094 16.218503 12.282393

C 5.253915 16.312696 11.079484

H 5.323700 17.401656 11.093626

C 6.222401 15.559907 10.409121

C 7.477015 11.914849 12.083960

H 6.885765 12.561783 12.753673

H 7.728512 11.037555 12.695154

C 6.745407 10.069128 10.551652

C 6.114978 9.184666 11.647084

H 6.232614 8.126165 11.370463

H 5.036751 9.373067 11.762530

H 6.594689 9.317447 12.628894

C 9.618411 15.204410 9.210810

H 10.171055 16.143901 9.383833

H 10.117214 14.681506 8.384101

C 8.068181 16.048151 7.429661

C 6.572651 16.189593 7.102766

H 6.462449 16.584488 6.082355

H 6.047599 16.885065 7.773145

H 6.080702 15.210472 7.144241

C 5.265307 12.040103 11.068925

H 4.791808 11.671957 11.995803

H 4.658190 11.657239 10.237835

C 7.482237 16.207290 9.862872

H 7.245758 17.226736 9.530082

H 8.139665 16.345867 10.737924

C 8.228980 9.702521 10.368115

H 8.301034 8.652015 10.049749

H 8.809928 9.794309 11.297455

H 8.686798 10.336131 9.595127

C 6.024493 9.800848 9.218637

H 6.222788 8.763411 8.911706

H 6.381909 10.472601 8.427009

H 4.931843 9.901321 9.297513

C 8.754735 17.427149 7.341111

H 8.604799 17.840941 6.332647

H 9.840362 17.361001 7.507606

H 8.340548 18.155995 8.054484

C 8.695061 15.112567 6.380553

H 8.443788 15.486346 5.377129

H 8.308140 14.091075 6.479492

H 9.792595 15.079184 6.442107

**^S^3** (1/1)

Mn 3.295322 3.890698 11.063030

O 3.494826 5.117251 12.320439

H 2.814615 5.826401 12.159284

O 2.018303 5.022616 9.989250

O 1.771466 6.846465 11.271410

N 5.282399 4.222722 10.192430

N 4.395959 2.603116 12.065541

N 1.689757 2.440239 11.643033

N 3.189148 2.641017 9.476387

N -1.995249 8.411818 6.325591

H -2.577662 9.196922 6.591775

H -2.398786 7.804640 5.622095

C 0.594536 6.746414 9.207802

C 0.010570 8.012328 9.400643

H 0.251079 8.562704 10.312419

C -0.854979 8.560836 8.462378

H -1.296842 9.546415 8.633694

C -1.176337 7.856537 7.276460

C -0.592491 6.581090 7.083687

H -0.827878 6.017434 6.176596

C 0.272493 6.045051 8.031068

H 0.714235 5.060545 7.866758

C 1.522275 6.184192 10.237042

C 3.833026 1.504504 12.583314

C 4.578071 0.653905 13.403518

H 4.118547 -0.234200 13.840395

C 5.924640 0.954955 13.626494

H 6.529865 0.306025 14.262996

C 6.509263 2.062967 13.001895

H 7.571712 2.282005 13.117988

C 5.701372 2.876934 12.209141

C 4.255726 2.498209 8.682299

C 4.165165 1.752885 7.505097

H 5.028648 1.648993 6.846412

C 2.949802 1.128613 7.208905

H 2.849570 0.537087 6.296340

C 1.873221 1.228226 8.097525

H 0.935750 0.702629 7.909705

C 2.031623 2.006239 9.244308

C 2.439657 1.212851 12.083368

H 2.556487 0.543745 11.216659

H 1.871703 0.634805 12.821791

C 0.688504 2.875933 12.748517

C 1.448594 3.037818 14.075882

H 0.757659 3.450725 14.825210

H 2.286887 3.739128 13.962962

H 1.824756 2.087280 14.479354

C 6.156767 4.066557 11.403780

H 6.053694 4.972934 12.011975

H 7.216332 3.954942 11.129884

C 5.553939 5.574878 9.469806

C 5.168889 6.773014 10.351056

H 5.436315 7.688865 9.803143

H 5.721004 6.798100 11.300699

H 4.098264 6.810176 10.571919

C 5.525926 3.076491 9.248291

H 6.016123 2.262219 9.804277

H 6.227251 3.349968 8.451683

C 1.005296 2.176424 10.337523

H 0.379437 3.045799 10.100835

H 0.356914 1.288152 10.385401

C -0.435294 1.828794 12.908914

H -1.054778 2.114154 13.771451

H -0.065216 0.811569 13.102640

H -1.097887 1.794695 12.031942

C 0.038523 4.223348 12.409616

H -0.708269 4.444085 13.186449

H -0.494215 4.217515 11.449002

H 0.763707 5.042923 12.405011

C 4.713981 5.623947 8.182668

H 4.806372 6.627813 7.743004

H 3.653343 5.439943 8.396814

H 5.056865 4.910571 7.419857

C 7.051853 5.714988 9.118406

H 7.178018 6.619450 8.505860

H 7.447717 4.873938 8.531035

H 7.680129 5.840034 10.012075

**^T^3** (1/3)

Mn 3.269605 3.862518 11.043076

O 3.446675 5.000542 12.480810

H 2.955043 5.815883 12.250873

O 2.077884 5.024500 10.008150

O 1.882497 6.909145 11.220839

N 5.288769 4.250366 10.173594

N 4.384484 2.563992 12.017563

N 1.673571 2.464522 11.653305

N 3.181533 2.627698 9.475162

N -2.026114 8.264757 6.326153

H -2.595149 9.067800 6.565424

H -2.436717 7.635174 5.647224

C 0.641779 6.723457 9.203214

C 0.060531 7.996554 9.358570

H 0.324045 8.584390 10.240138

C -0.830125 8.504899 8.422482

H -1.269293 9.496204 8.564941

C -1.180850 7.751649 7.274660

C -0.598806 6.469912 7.118754

H -0.855662 5.869873 6.241402

C 0.291148 5.973909 8.064195

H 0.731526 4.984909 7.926867

C 1.594707 6.211842 10.233170

C 3.811741 1.482318 12.559737

C 4.555118 0.635380 13.383510

H 4.088866 -0.239417 13.839398

C 5.907668 0.924415 13.588332

H 6.512710 0.276167 14.225568

C 6.494579 2.024730 12.953979

H 7.557928 2.241324 13.065837

C 5.687799 2.842432 12.163139

C 4.238783 2.513825 8.664308

C 4.136341 1.778021 7.481644

H 4.989748 1.690827 6.807472

C 2.923298 1.142792 7.197511

H 2.817112 0.558410 6.281118

C 1.853912 1.229533 8.096614

H 0.915200 0.705922 7.909507

C 2.020201 1.999388 9.247864

C 2.404514 1.219485 12.092100

H 2.483725 0.538516 11.230816

H 1.833695 0.669235 12.848655

C 0.664420 2.911526 12.748390

C 1.411821 3.038148 14.086497

H 0.723278 3.468216 14.828730

H 2.270234 3.715053 13.975303

H 1.748237 2.071765 14.488219

C 6.147015 4.051374 11.389172

H 6.031389 4.935591 12.027310

H 7.209539 3.949897 11.123030

C 5.581669 5.613952 9.491121

C 5.209856 6.780503 10.418490

H 5.436081 7.717550 9.888110

H 5.801920 6.790365 11.344255

H 4.148140 6.788963 10.683945

C 5.513417 3.114124 9.205358

H 6.040734 2.304489 9.733256

H 6.181133 3.412177 8.389061

C 0.993852 2.192704 10.340008

H 0.372204 3.062153 10.092050

H 0.338738 1.311311 10.403806

C -0.487133 1.892576 12.875958

H -1.104025 2.175884 13.741057

H -0.143092 0.862495 13.051147

H -1.145477 1.893364 11.995086

C 0.068084 4.283322 12.409262

H -0.686465 4.524312 13.172560

H -0.443705 4.304812 11.437199

H 0.831096 5.067829 12.429697

C 4.743522 5.710601 8.205156

H 4.844957 6.725908 7.795129

H 3.681098 5.527062 8.408801

H 5.081847 5.016584 7.422558

C 7.079703 5.743337 9.140234

H 7.219216 6.670919 8.566166

H 7.456251 4.920443 8.515289

H 7.714425 5.818899 10.034820

**^Q^3** (1/5)

Mn 3.181362 4.021268 11.071453

O 3.327121 5.133723 12.481857

H 2.798704 5.941484 12.263024

O 1.996825 5.125056 10.035253

O 1.759968 6.976744 11.282201

N 5.412888 4.300631 10.094424

N 4.374733 2.621202 12.021935

N 1.568182 2.315099 11.732474

N 3.172615 2.649497 9.521311

N -1.974840 8.429334 6.278852

H -2.527639 9.250589 6.491096

H -2.348751 7.822234 5.560040

C 0.591211 6.845280 9.215453

C 0.013065 8.121118 9.368194

H 0.250197 8.698564 10.263959

C -0.842835 8.644335 8.409988

H -1.280797 9.636523 8.548404

C -1.161071 7.904463 7.242812

C -0.581601 6.619853 7.090744

H -0.814603 6.031119 6.199425

C 0.273912 6.108456 8.057547

H 0.711112 5.117435 7.924265

C 1.500240 6.317467 10.264472

C 3.823070 1.535894 12.586214

C 4.592263 0.706724 13.407015

H 4.140460 -0.166725 13.880309

C 5.945042 1.003144 13.583401

H 6.565893 0.368344 14.219280

C 6.511663 2.090841 12.913629

H 7.577729 2.309523 12.992069

C 5.686703 2.894898 12.125838

C 4.230164 2.545373 8.699162

C 4.137646 1.781240 7.532643

H 4.989856 1.711753 6.854557

C 2.951384 1.093755 7.270385

H 2.857783 0.489084 6.365729

C 1.898324 1.149706 8.187207

H 0.983039 0.576566 8.031836

C 2.041089 1.948592 9.322369

C 2.409689 1.174526 12.171125

H 2.521101 0.466415 11.333369

H 1.931458 0.591248 12.969182

C 0.537599 2.719737 12.790432

C 1.263637 2.934087 14.131336

H 0.545257 3.330221 14.864164

H 2.075362 3.666359 14.015152

H 1.671380 2.005339 14.555297

C 6.196536 4.081114 11.334089

H 6.099832 4.975820 11.963610

H 7.271623 3.933786 11.135376

C 5.716341 5.638612 9.415892

C 5.401040 6.805567 10.368120

H 5.531185 7.749839 9.819250

H 6.081880 6.844066 11.230731

H 4.370997 6.767885 10.742950

C 5.540462 3.150694 9.163685

H 6.079791 2.334216 9.671872

H 6.160087 3.392680 8.290077

C 0.977517 2.058036 10.396062

H 0.317747 2.898648 10.140331

H 0.361328 1.143107 10.373504

C -0.553873 1.640861 12.945431

H -1.222172 1.916341 13.774769

H -0.138772 0.648352 13.178873

H -1.175843 1.549509 12.042203

C -0.126444 4.053685 12.410942

H -0.876876 4.299589 13.176808

H -0.655658 4.018195 11.448276

H 0.604154 4.871343 12.386290

C 4.817030 5.772405 8.173912

H 4.960672 6.769298 7.732232

H 3.756495 5.664786 8.439512

H 5.059348 5.039169 7.391225

C 7.198042 5.736592 8.995883

H 7.362173 6.690062 8.472026

H 7.499781 4.933411 8.306735

H 7.875986 5.720218 9.862359

**^S^4** (1/1)

Mn 9.846469 12.046469 2.818649

O 10.094170 13.427023 1.742953

O 9.256638 13.210707 4.371887

O 9.493745 15.299158 3.583963

O 7.262340 17.772635 9.647216

N 11.757432 11.490399 3.829162

N 9.307943 10.526413 4.033154

O 7.023593 15.761643 10.396488

N 8.003252 11.400485 1.812752

N 10.539724 10.797638 1.471104

N 7.327368 16.558662 9.520873

C 9.188454 14.483950 4.475654

C 8.695211 15.011733 5.807381

C 8.598828 16.399112 6.002062

H 8.881128 17.064617 5.185506

C 8.151099 16.915143 7.216073

H 8.069707 17.987939 7.386089

C 7.801718 16.022290 8.232302

C 7.887243 14.637884 8.067648

H 7.604479 13.977155 8.886075

C 8.337187 14.138087 6.846846

H 8.413715 13.062257 6.687566

C 7.052624 13.759123 1.537627

H 6.110126 14.325111 1.578681

H 7.438222 13.856574 0.513537

H 7.762293 14.234679 2.220538

C 6.740009 12.304751 1.921255

C 6.235576 12.278054 3.373609

H 5.425243 13.014452 3.475987

H 7.034010 12.549340 4.075765

H 5.818093 11.303789 3.664519

C 5.626160 11.801229 0.976354

H 4.715491 12.383902 1.177090

H 5.366966 10.742930 1.124180

H 5.878878 11.951650 -0.082991

C 8.473905 11.292849 0.390066

H 8.562704 12.308142 -0.013973

H 7.757994 10.736884 -0.233161

C 9.823545 10.623905 0.350017

C 10.322712 9.841244 -0.690095

H 9.741755 9.695775 -1.601998

C 11.575251 9.238485 -0.524723

H 11.996886 8.629439 -1.327079

C 12.268550 9.380411 0.680575

H 13.221955 8.876168 0.846041

C 11.700348 10.165607 1.686251

C 12.213788 10.254374 3.101560

H 11.819762 9.372354 3.629818

H 13.304272 10.147914 3.131411

C 12.899793 12.545527 3.848739

C 13.379070 12.790889 2.408038

H 14.104955 13.616813 2.418563

H 12.541421 13.083453 1.759923

H 13.890400 11.923693 1.967671

C 14.076843 12.055709 4.719649

H 14.906055 12.770250 4.615111

H 14.463338 11.070103 4.422591

H 13.814788 12.018096 5.786877

C 12.410379 13.882903 4.417914

H 13.273773 14.561360 4.479878

H 12.000433 13.797270 5.433481

H 11.664611 14.357752 3.772951

C 11.305296 11.124582 5.209513

H 11.095563 12.051766 5.757107

H 12.079400 10.569835 5.761397

C 10.039979 10.305084 5.134678

C 9.638404 9.347264 6.065455

H 10.236617 9.156850 6.957760

C 8.462723 8.630360 5.815295

H 8.116936 7.883989 6.533636

C 7.750272 8.839989 4.630805

H 6.856491 8.258828 4.399091

C 8.224620 9.800222 3.735312

C 7.698719 10.025071 2.342675

H 6.629330 9.793878 2.279171

H 8.198356 9.283327 1.700452

H 10.045376 14.259617 2.277803

**^T^4** (1/3)

Mn 9.882336 12.033371 2.843695

O 10.288658 13.399996 1.744290

O 9.251800 13.193792 4.337323

O 9.404423 15.281960 3.529417

O 7.174388 17.714162 9.612573

N 11.809531 11.511371 3.822666

N 9.362483 10.493214 4.032114

O 7.020777 15.700478 10.376895

N 7.958366 11.412095 1.836055

N 10.525596 10.743393 1.509574

N 7.282166 16.502672 9.492455

C 9.142451 14.469297 4.431891

C 8.651218 14.983786 5.768157

C 8.511539 16.368182 5.957612

H 8.760322 17.038752 5.134344

C 8.063341 16.874915 7.175311

H 7.948565 17.945230 7.341562

C 7.757315 15.975936 8.200023

C 7.886385 14.594361 8.040532

H 7.636495 13.928612 8.865541

C 8.336453 14.103619 6.816139

H 8.445755 13.029966 6.661440

C 7.051305 13.754315 1.478924

H 6.137242 14.361687 1.555291

H 7.384333 13.813952 0.433179

H 7.816086 14.212451 2.113638

C 6.707174 12.320669 1.910087

C 6.203592 12.345917 3.363256

H 5.404989 13.097158 3.447848

H 7.007795 12.620030 4.057291

H 5.771690 11.386393 3.681873

C 5.584251 11.801315 0.986218

H 4.686750 12.415126 1.151399

H 5.300140 10.758025 1.188287

H 5.844514 11.889759 -0.078432

C 8.451074 11.267464 0.428459

H 8.572508 12.273620 0.007871

H 7.736361 10.718168 -0.203155

C 9.791860 10.577072 0.399597

C 10.284842 9.803705 -0.651257

H 9.692999 9.656414 -1.555726

C 11.548554 9.220071 -0.509203

H 11.963579 8.619297 -1.321291

C 12.271330 9.378198 0.677520

H 13.242753 8.900796 0.814447

C 11.711656 10.149913 1.696950

C 12.272946 10.277705 3.089590

H 11.928328 9.390288 3.643414

H 13.366290 10.203707 3.076557

C 12.941581 12.572386 3.860064

C 13.433730 12.823141 2.424320

H 14.149301 13.658242 2.442522

H 12.592415 13.103117 1.776127

H 13.962502 11.961997 1.991611

C 14.112629 12.095263 4.744147

H 14.938341 12.814804 4.645219

H 14.507649 11.111157 4.451263

H 13.842974 12.057394 5.809663

C 12.418631 13.901977 4.419319

H 13.264918 14.601410 4.486674

H 12.000750 13.810599 5.431541

H 11.667293 14.347736 3.760009

C 11.363688 11.119125 5.197937

H 11.141612 12.034562 5.761519

H 12.147220 10.570325 5.742372

C 10.107411 10.283847 5.127204

C 9.701511 9.347370 6.077710

H 10.305568 9.161800 6.966944

C 8.507603 8.652179 5.854680

H 8.160133 7.921114 6.587835

C 7.768963 8.870466 4.687619

H 6.851587 8.314436 4.488939

C 8.242693 9.808598 3.768401

C 7.656705 10.047721 2.399665

H 6.580406 9.840458 2.399946

H 8.097289 9.286361 1.736870

H 10.032094 14.235039 2.201438

**^Q^4** (1/5)

Mn 9.849393 12.200358 2.886144

O 10.294523 13.544198 1.771009

O 9.250788 13.320308 4.357009

O 9.448798 15.417082 3.578805

O 7.160871 17.779932 9.673467

N 11.940795 11.446757 3.872530

N 9.345064 10.551979 4.017719

O 6.991067 15.755093 10.404707

N 7.826995 11.396602 1.770716

N 10.507706 10.808016 1.509184

N 7.264034 16.570317 9.536536

C 9.160585 14.612389 4.467956

C 8.660314 15.104838 5.803401

C 8.524512 16.487120 6.010623

H 8.782682 17.170651 5.200971

C 8.067057 16.975774 7.232011

H 7.953915 18.043617 7.414035

C 7.749605 16.061231 8.239113

C 7.875472 14.682112 8.061055

H 7.616572 14.004999 8.873857

C 8.334235 14.207856 6.833248

H 8.442169 13.136508 6.663129

C 6.923901 13.711985 1.351300

H 6.041439 14.351559 1.499027

H 7.168099 13.750968 0.279952

H 7.759396 14.150291 1.910467

C 6.584258 12.290071 1.831140

C 6.110895 12.372339 3.293357

H 5.278163 13.087325 3.361451

H 6.916417 12.722186 3.953060

H 5.738078 11.411424 3.675960

C 5.445639 11.735144 0.949768

H 4.559305 12.377491 1.060045

H 5.143416 10.716118 1.234994

H 5.713233 11.729779 -0.117353

C 8.389414 11.270390 0.403605

H 8.492892 12.278268 -0.020026

H 7.733243 10.696415 -0.272103

C 9.757095 10.620399 0.410103

C 10.237061 9.839528 -0.642151

H 9.624346 9.681291 -1.530933

C 11.504647 9.263282 -0.524648

H 11.910057 8.661972 -1.341211

C 12.235518 9.425608 0.653879

H 13.205612 8.943646 0.786158

C 11.690834 10.198563 1.682319

C 12.307660 10.255257 3.066220

H 11.953871 9.348358 3.583818

H 13.395427 10.128293 2.988855

C 13.078636 12.470646 3.946631

C 13.559039 12.780370 2.516912

H 14.293546 13.598037 2.559360

H 12.717154 13.107661 1.890269

H 14.059527 11.928043 2.035817

C 14.251203 11.938243 4.795239

H 15.086284 12.652845 4.748669

H 14.631151 10.970714 4.433063

H 13.977471 11.825706 5.855080

C 12.575269 13.788560 4.557688

H 13.421994 14.487393 4.625821

H 12.177246 13.672698 5.575704

H 11.810894 14.255549 3.924633

C 11.416219 11.045597 5.201754

H 11.222351 11.954122 5.788581

H 12.136310 10.437745 5.775049

C 10.119905 10.270434 5.082416

C 9.732661 9.287205 5.993270

H 10.365090 9.055192 6.851506

C 8.533660 8.603966 5.773002

H 8.201060 7.840724 6.479770

C 7.782145 8.871220 4.627457

H 6.869013 8.314441 4.410663

C 8.232020 9.851835 3.739404

C 7.593643 10.062138 2.381048

H 6.525127 9.816746 2.438016

H 8.024845 9.286260 1.727061

H 10.071974 14.395226 2.215636

Mn^III^–OH for **1** (1/5)

Mn 5.489249 3.689295 4.678993

O 3.250752 1.333900 4.093901

O 4.144308 3.256240 3.359715

O 5.162512 2.296858 5.775433

N 6.027373 5.306477 3.512408

N 7.629269 2.971806 3.734929

N 6.925361 4.408821 5.981003

N 4.368534 5.603848 5.686801

C 4.015693 6.322556 4.437565

C 5.168395 6.341207 3.454336

C 5.374150 7.375623 2.540840

C 6.486232 7.313302 1.697087

C 7.394859 6.260853 1.821845

C 7.148050 5.266176 2.771626

C 9.033816 1.340969 2.384931

C 6.848378 2.219292 1.490489

C 6.865495 0.595469 3.383855

C 7.606245 1.791132 2.760029

C 8.176085 4.206519 3.115601

C 8.315882 2.661721 5.012765

C 8.085794 3.734701 6.056526

C 9.018432 4.051738 7.045194

C 8.718437 5.078442 7.944437

C 7.532840 5.801977 7.802740

C 6.650164 5.453178 6.777469

C 5.451107 6.308810 6.417617

C 2.413879 6.596065 6.962726

C 3.656297 4.585016 7.851852

C 3.162460 5.302294 6.582061

C 2.188574 4.350971 5.867556

C 2.430913 3.341849 1.158533

C 1.557491 3.350515 0.068328

C 0.631595 2.313740 -0.096238

C 0.579624 1.266593 0.832165

C 1.451366 1.256660 1.922200

C 2.383489 2.293937 2.092903

C 3.307482 2.258835 3.276335

H 3.169734 5.804683 3.964853

H 3.696522 7.362130 4.622705

H 4.680849 8.217065 2.499713

H 6.660201 8.102170 0.962171

H 8.295052 6.219003 1.206612

H 9.630875 2.147958 1.933884

H 9.584913 0.948130 3.252375

H 8.973532 0.530467 1.643400

H 5.842783 2.588363 1.734185

H 7.377449 2.995118 0.918664

H 6.741966 1.349793 0.825637

H 7.401656 0.164955 4.241797

H 5.851680 0.859009 3.708471

H 6.786049 -0.199614 2.628013

H 8.871924 4.686681 3.823438

H 8.785881 3.988015 2.229181

H 7.910011 1.717693 5.400354

H 9.402982 2.524122 4.886335

H 9.963558 3.509437 7.101777

H 9.423959 5.334388 8.737846

H 7.301450 6.639964 8.462352

H 5.846342 7.118076 5.781779

H 5.079608 6.815803 7.317875

H 3.067460 7.336713 7.448671

H 1.941885 7.073362 6.090714

H 1.611742 6.354405 7.675769

H 4.233600 3.687844 7.586618

H 4.265490 5.228959 8.501986

H 2.783779 4.270129 8.443018

H 1.794607 4.757455 4.925385

H 2.654162 3.377659 5.670337

H 1.323643 4.178854 6.525051

H 3.157060 4.144276 1.296031

H 1.598275 4.167821 -0.655939

H -0.051158 2.321458 -0.949788

H -0.143074 0.456942 0.704169

H 1.423940 0.449014 2.655718

H 4.453141 1.752083 5.357241

Mn^III^–OH_2_ for **1** (2/5)

Mn 5.646663 3.809319 4.661063

O 3.559712 1.302033 4.325944

O 4.333005 3.199834 3.424841

O 5.302080 2.237300 5.777247

N 6.067631 5.352166 3.439427

N 7.761263 3.102267 3.749924

N 6.991438 4.574927 5.935488

N 4.391448 5.630224 5.594312

C 3.998350 6.292440 4.321819

C 5.141993 6.325700 3.331988

C 5.283961 7.310797 2.356215

C 6.393489 7.262051 1.508039

C 7.363326 6.272506 1.682184

C 7.184076 5.322881 2.688401

C 9.260915 1.495082 2.474822

C 7.053114 2.237711 1.512397

C 7.113442 0.675019 3.455073

C 7.805869 1.884765 2.806809

C 8.260943 4.337880 3.085200

C 8.478869 2.866854 5.030092

C 8.187433 3.954881 6.038409

C 9.090691 4.349582 7.023099

C 8.737357 5.392156 7.883615

C 7.523434 6.059140 7.708878

C 6.662346 5.640564 6.695074

C 5.436440 6.427143 6.294192

C 2.371933 6.610346 6.780646

C 3.696804 4.750344 7.837843

C 3.186919 5.330511 6.507041

C 2.271173 4.283341 5.855305

C 2.578984 3.015629 1.258667

C 1.689737 2.882217 0.191614

C 0.827657 1.780733 0.131886

C 0.852910 0.809488 1.140777

C 1.739085 0.937902 2.209640

C 2.607652 2.042480 2.273380

C 3.542436 2.160445 3.421053

H 5.423803 2.051976 6.720213

H 3.165877 5.732013 3.874326

H 3.644422 7.323260 4.481142

H 4.541335 8.105207 2.270723

H 6.516467 8.014987 0.726941

H 8.259478 6.244859 1.060740

H 9.834056 2.319804 2.025765

H 9.807016 1.135353 3.359254

H 9.251033 0.674042 1.743241

H 6.027228 2.570814 1.720810

H 7.559444 3.011377 0.918440

H 6.993728 1.340070 0.880518

H 7.614925 0.329032 4.369755

H 6.060270 0.869394 3.688503

H 7.142601 -0.162405 2.743292

H 8.923753 4.881416 3.778419

H 8.889260 4.110497 2.215540

H 8.149773 1.904303 5.445323

H 9.569916 2.799266 4.893580

H 10.058847 3.853728 7.104038

H 9.424704 5.706813 8.671462

H 7.251586 6.910516 8.334350

H 5.799678 7.220587 5.620720

H 5.035332 6.960277 7.164835

H 2.986233 7.426326 7.190218

H 1.863271 6.979059 5.878124

H 1.593272 6.386165 7.524116

H 4.344913 3.878135 7.677280

H 4.240897 5.481152 8.451543

H 2.834371 4.413961 8.430438

H 1.893531 4.587124 4.869252

H 2.765923 3.308797 5.763891

H 1.392150 4.139401 6.499837

H 3.254789 3.870074 1.313666

H 1.667778 3.638242 -0.596475

H 0.132438 1.678257 -0.705059

H 0.179266 -0.049029 1.091832

H 1.770470 0.189898 3.003301

H 4.530827 1.681990 5.320218

Mn^II^–OH for **1** (0/6)

Mn 5.384595 3.487070 4.770474

O 2.839165 1.374451 3.926659

O 3.891958 3.225011 3.200233

O 4.929060 1.921926 5.880298

N 6.029849 5.265084 3.534648

N 7.730436 2.958475 3.784765

N 7.004355 4.450123 6.035678

N 4.381010 5.589479 5.763779

C 4.023826 6.258249 4.491871

C 5.180032 6.300058 3.504838

C 5.376753 7.361256 2.615660

C 6.480178 7.313291 1.757619

C 7.384808 6.254315 1.852220

C 7.138325 5.242936 2.791453

C 9.116126 1.358641 2.372685

C 6.794011 2.053963 1.679272

C 7.116955 0.532679 3.635074

C 7.707329 1.739620 2.878472

C 8.192368 4.184715 3.100707

C 8.438501 2.741373 5.064278

C 8.206176 3.859576 6.067955

C 9.186260 4.280416 6.972503

C 8.880189 5.331379 7.842957

C 7.644282 5.973112 7.744948

C 6.725242 5.515901 6.790315

C 5.465345 6.310217 6.468178

C 2.475407 6.575478 7.122355

C 3.702645 4.494747 7.884225

C 3.193263 5.287310 6.665457

C 2.179527 4.386478 5.938024

C 2.317649 3.315165 0.878085

C 1.506395 3.334190 -0.260442

C 0.513314 2.362088 -0.430141

C 0.335797 1.371429 0.543540

C 1.149193 1.354505 1.679559

C 2.147421 2.325347 1.859030

C 3.023627 2.290020 3.103435

H 3.211856 5.686319 4.022025

H 3.655712 7.289923 4.641596

H 4.687825 8.208121 2.600556

H 6.650003 8.117167 1.037316

H 8.278901 6.222696 1.225780

H 9.577112 2.148214 1.759483

H 9.800048 1.125496 3.203394

H 9.048377 0.458444 1.742597

H 5.785634 2.337408 2.015535

H 7.188792 2.867294 1.052058

H 6.712151 1.163964 1.037148

H 7.839450 0.091517 4.339091

H 6.214712 0.808375 4.202232

H 6.859358 -0.254677 2.910713

H 8.934048 4.693330 3.740340

H 8.740873 3.954603 2.175823

H 8.063608 1.808587 5.508242

H 9.529532 2.614970 4.932303

H 10.167861 3.802768 6.989631

H 9.617556 5.669809 8.574825

H 7.404529 6.829535 8.378884

H 5.806819 7.146283 5.834437

H 5.105398 6.792746 7.388163

H 3.139700 7.259473 7.672658

H 2.043932 7.127431 6.272873

H 1.646414 6.315918 7.798220

H 4.181079 3.562345 7.545843

H 4.409806 5.065530 8.503776

H 2.848836 4.234821 8.528616

H 1.715369 4.873755 5.068443

H 2.643954 3.445380 5.615306

H 1.365699 4.140540 6.637133

H 3.096285 4.065659 1.025159

H 1.647869 4.108846 -1.019071

H -0.121645 2.376287 -1.320115

H -0.438981 0.610718 0.414502

H 1.026316 0.590421 2.449528

H 4.167939 1.547671 5.399241

Mn^II^–OH_2_ for **1** (1/6)

Mn 5.554029 3.770411 4.629713

O 3.348461 1.156845 4.206938

O 4.009861 3.113100 3.337160

O 5.080792 2.032828 5.842161

N 6.055966 5.493051 3.377260

N 7.845376 3.254534 3.708457

N 7.049872 4.740978 5.938488

N 4.385467 5.759819 5.595547

C 4.007439 6.412902 4.314030

C 5.166892 6.496618 3.334355

C 5.339470 7.558294 2.443483

C 6.458474 7.545007 1.603768

C 7.401661 6.521896 1.715909

C 7.178747 5.504784 2.653311

C 9.366491 1.741907 2.329242

C 6.996362 2.209115 1.615072

C 7.457991 0.763772 3.602191

C 7.927774 2.009019 2.824380

C 8.262533 4.487592 2.987654

C 8.585402 3.108963 4.988075

C 8.288053 4.224933 5.974705

C 9.243226 4.723703 6.863593

C 8.878397 5.774997 7.710457

C 7.610275 6.347240 7.597748

C 6.715594 5.817220 6.659666

C 5.428928 6.546157 6.301465

C 2.416083 6.736744 6.874757

C 3.697458 4.756738 7.773552

C 3.188215 5.454184 6.497705

C 2.215573 4.484846 5.803613

C 2.345443 2.777374 1.095774

C 1.501064 2.571993 0.001768

C 0.714723 1.416030 -0.069202

C 0.775366 0.465495 0.957128

C 1.619675 0.670881 2.050380

C 2.411355 1.828435 2.128818

C 3.319090 2.039051 3.313713

H 5.560435 1.418404 6.413164

H 3.209543 5.817661 3.848013

H 3.605162 7.429643 4.464513

H 4.621903 8.380132 2.413041

H 6.610635 8.352102 0.883482

H 8.306593 6.523918 1.105138

H 9.752839 2.541528 1.679987

H 10.070865 1.605313 3.163727

H 9.378513 0.814502 1.737212

H 5.950327 2.343841 1.927705

H 7.285972 3.072877 0.998778

H 7.047108 1.321316 0.967381

H 8.199460 0.425184 4.340818

H 6.503888 0.924517 4.121238

H 7.318585 -0.064337 2.891995

H 8.991567 5.033960 3.609553

H 8.812464 4.247640 2.068201

H 8.293295 2.155679 5.450222

H 9.678261 3.060886 4.837253

H 10.251188 4.305700 6.885563

H 9.596575 6.171997 8.431514

H 7.327085 7.208703 8.205771

H 5.746089 7.379259 5.652245

H 5.031379 7.035819 7.200590

H 3.051112 7.485572 7.371918

H 1.950561 7.209841 5.996984

H 1.606587 6.482759 7.575227

H 4.239355 3.831574 7.528736

H 4.347970 5.397399 8.385647

H 2.835296 4.484470 8.400273

H 1.782095 4.894235 4.880516

H 2.691140 3.523433 5.572679

H 1.377292 4.284107 6.487259

H 2.964675 3.673304 1.162041

H 1.455292 3.314655 -0.798868

H 0.054147 1.255402 -0.925251

H 0.162448 -0.437883 0.903135

H 1.678591 -0.060180 2.858438

H 4.376992 1.532119 5.293861

Mn^III^–OH for **2** (1/5)

Mn 7.393807 13.130101 9.149130

O 6.162230 12.823382 7.802871

O 8.707269 12.240066 8.217636

H 8.312300 11.876639 7.409789

N 8.770421 13.445050 10.671448

N 6.125364 14.218580 10.366779

C 4.787450 12.972607 7.663827

H 4.418065 13.918145 8.113408

H 4.240544 12.160937 8.191008

C 4.340962 12.938909 6.208234

C 5.224339 12.590605 5.178770

H 6.260669 12.363296 5.433275

C 4.785670 12.541265 3.849436

H 5.488148 12.268919 3.056775

C 3.456930 12.838563 3.533598

H 3.114355 12.800904 2.496216

C 2.567157 13.188467 4.557610

H 1.526377 13.426119 4.321213

C 3.007264 13.238629 5.882489

H 2.305811 13.515426 6.676163

N 6.652506 11.553773 10.907412

N 8.208352 15.438199 8.825385

C 8.743112 12.692275 11.783335

C 9.800363 12.745276 12.695894

H 9.782765 12.117985 13.588735

C 10.854414 13.627796 12.456616

H 11.689998 13.687549 13.157354

C 10.817799 14.459662 11.335507

H 11.603111 15.194339 11.151060

C 9.747592 14.341700 10.447060

C 5.184807 13.554175 11.067557

C 4.207076 14.252642 11.776757

H 3.451770 13.708688 12.346018

C 4.226501 15.649068 11.750807

H 3.461094 16.218503 12.282393

C 5.253915 16.312696 11.079484

H 5.323700 17.401656 11.093626

C 6.222401 15.559907 10.409121

C 7.477015 11.914849 12.083960

H 6.885765 12.561783 12.753673

H 7.728512 11.037555 12.695154

C 6.745407 10.069128 10.551652

C 6.114978 9.184666 11.647084

H 6.232614 8.126165 11.370463

H 5.036751 9.373067 11.762530

H 6.594689 9.317447 12.628894

C 9.618411 15.204410 9.210810

H 10.171055 16.143901 9.383833

H 10.117214 14.681506 8.384101

C 8.068181 16.048151 7.429661

C 6.572651 16.189593 7.102766

H 6.462449 16.584488 6.082355

H 6.047599 16.885065 7.773145

H 6.080702 15.210472 7.144241

C 5.265307 12.040103 11.068925

H 4.791808 11.671957 11.995803

H 4.658190 11.657239 10.237835

C 7.482237 16.207290 9.862872

H 7.245758 17.226736 9.530082

H 8.139665 16.345867 10.737924

C 8.228980 9.702521 10.368115

H 8.301034 8.652015 10.049749

H 8.809928 9.794309 11.297455

H 8.686798 10.336131 9.595127

C 6.024493 9.800848 9.218637

H 6.222788 8.763411 8.911706

H 6.381909 10.472601 8.427009

H 4.931843 9.901321 9.297513

C 8.754735 17.427149 7.341111

H 8.604799 17.840941 6.332647

H 9.840362 17.361001 7.507606

H 8.340548 18.155995 8.054484

C 8.695061 15.112567 6.380553

H 8.443788 15.486346 5.377129

H 8.308140 14.091075 6.479492

H 9.792595 15.079184 6.442107

Mn^III^–OH_2_ for **2** (2/5)

Mn 7.339088 13.156740 9.235944

O 6.186344 12.810579 7.909599

O 8.837038 12.307173 8.038319

H 9.697604 11.929212 8.281535

H 8.549552 11.884598 7.211694

N 8.763591 13.460698 10.640494

N 6.079290 14.208150 10.449029

C 4.799805 12.878252 7.670735

H 4.348572 13.753795 8.171106

H 4.309365 11.981002 8.095956

C 4.493920 12.938891 6.185651

C 5.184996 12.122754 5.275682

H 5.977466 11.463553 5.637489

C 4.867934 12.150177 3.914832

H 5.414645 11.510260 3.217263

C 3.851940 12.990880 3.444796

H 3.603966 13.011247 2.380550

C 3.158616 13.806502 4.344212

H 2.367154 14.469795 3.985911

C 3.481995 13.783322 5.705481

H 2.940984 14.430661 6.401701

N 6.676926 11.549510 10.921795

N 8.081853 15.418372 8.831964

C 8.777546 12.705896 11.757833

C 9.857866 12.785077 12.638328

H 9.877917 12.160656 13.532581

C 10.886214 13.688858 12.368377

H 11.738218 13.766135 13.046854

C 10.804796 14.521045 11.249139

H 1.569156 15.272414 11.046832

C 9.716516 14.385190 10.389260

C 5.169653 13.521049 11.165217

C 4.193548 14.202671 11.891109

H 3.459439 13.646745 12.476014

C 4.186963 15.599602 11.858775

H 3.421623 16.155162 12.404562

C 5.184832 16.284911 11.162837

H 5.231690 17.374969 11.171654

C 6.153298 15.550850 10.476001

C 7.537802 11.904996 12.085692

H 6.957429 12.526185 12.787972

H 7.821082 11.018433 12.666523

C 6.749116 10.047971 10.580095

C 6.194638 9.184461 11.731873

H 6.264401 8.124627 11.446484

H 5.135050 9.395929 11.937288

H 6.759324 9.302600 12.668487

C 9.520485 15.250853 9.165088

H 10.024195 16.215627 9.336193

H 10.027254 14.778965 8.311806

C 7.870408 16.027470 7.433469

C 6.370591 16.291219 7.220547

H 6.211379 16.603245 6.178614

H 5.974814 17.093741 7.857990

H 5.780507 15.381669 7.385672

C 5.283513 12.011092 11.149782

H 4.866498 11.624154 12.093819

H 4.646624 11.619756 10.345602

C 7.393908 16.198665 9.894973

H 7.156825 17.218285 9.566883

H 8.079522 16.330740 10.748313

C 8.214407 9.668754 10.311260

H 8.269387 8.592395 10.094762

H 8.876231 9.856838 11.168297

H 8.610136 10.200047 9.435963

C 5.936296 9.749883 9.307894

H 6.136701 8.713457 9.000346

H 6.216209 10.409997 8.475823

H 4.851082 9.822593 9.469289

C 8.652057 17.348173 7.284927

H 8.386868 17.815282 6.325170

H 9.740476 17.190606 7.279792

H 8.410922 18.073535 8.077157

C 8.329989 15.038471 6.349943

H 8.205722 15.515773 5.366974

H 7.715580 14.129100 6.360729

H 9.389488 14.759908 6.436511

Mn^II^–OH for **2** (0/6)

Mn 7.557370 13.062931 8.867998

O 6.096291 12.871562 7.451724

O 9.021963 12.097424 7.881295

H 8.616722 11.840897 7.039788

N 8.805833 13.551475 10.807589

N 6.227465 14.236740 10.393802

C 4.794422 13.286641 7.407694

H 4.683196 14.395649 7.528061

H 4.172125 12.867880 8.241050

C 4.063633 12.926025 6.112771

C 4.718530 12.227899 5.089965

H 5.762903 11.953409 5.252150

C 4.050553 11.896203 3.903721

H 4.578547 11.350333 3.115971

C 2.712603 12.259633 3.722851

H 2.189205 12.001799 2.798083

C 2.046991 12.958714 4.739710

H 1.000119 13.248016 4.608942

C 2.718242 13.286691 5.920351

H 2.189839 13.833059 6.709586

N 6.839709 11.411212 10.727676

N 8.250259 15.550781 8.771785

C 8.639205 12.774589 11.885555

C 9.104013 13.171164 13.146054

H 8.963408 12.522482 14.012895

C 9.726010 14.413730 13.271993

H 10.098230 14.749931 14.242649

C 9.824837 15.241463 12.152651

H 10.257870 16.240746 12.228767

C 9.336129 14.776128 10.925721

C 5.658783 13.553145 11.395832

C 5.089755 14.212830 12.492055

H 4.626387 13.640990 13.298445

C 5.148017 15.606578 12.540892

H 4.713251 16.148380 13.384178

C 5.808928 16.296810 11.523979

H 5.917172 17.382614 11.560426

C 6.354624 15.568186 10.459385

C 7.952378 11.432588 11.698097

H 7.632350 11.072126 12.693355

H 8.724177 10.734062 11.349823

C 6.573999 10.003499 10.187882

C 6.123920 9.036928 11.303427

H 5.954425 8.037078 10.874637

H 5.180185 9.353410 11.774320

H 6.884362 8.927812 12.092251

C 9.413502 15.637813 9.677476

H 9.608281 16.679744 9.992623

H 10.303177 15.308693 9.125275

C 8.589150 16.002255 7.349060

C 7.382638 15.738024 6.427864

H 7.686664 15.908605 5.384210

H 6.539527 16.418525 6.619497

H 7.029019 14.700333 6.533185

C 5.627541 12.036933 11.294087

H 5.389511 11.633521 12.295761

H 4.777529 11.784500 10.646487

C 7.087908 16.272187 9.328808

H 6.355002 16.415995 8.523768

H 7.366029 17.282748 9.681106

C 7.855710 9.459057 9.524033

H 7.601822 8.558388 8.944937

H 8.617448 9.150104 10.256235

H 8.297220 10.209891 8.847887

C 5.486434 10.065422 9.097958

H 5.424869 9.085328 8.601706

H 5.727471 10.832525 8.345551

H 4.484374 10.270314 9.503436

C 8.957585 17.499701 7.294530

H 9.213009 17.776205 6.259831

H 9.832419 17.736265 7.920036

H 8.123563 18.146708 7.607812

C 9.768304 15.165733 6.810533

H 9.850547 15.325031 5.724825

H 9.610264 14.091965 7.004716

H 10.735720 15.466593 7.240940

Mn^II^–OH_2_ for **2** (1/6)

Mn 7.049248 13.307078 9.179374

O 6.123099 12.791513 7.475347

O 8.592843 12.295803 7.569030

H 8.978286 11.411062 7.633855

H 7.691835 12.187991 7.166400

N 8.786943 13.366541 10.545197

N 6.182987 14.457806 10.851704

C 4.836613 12.905306 6.981628

H 4.449220 13.946307 7.078269

H 4.115301 12.282919 7.559423

C 4.685192 12.514545 5.514932

C 5.791419 12.242938 4.699425

H 6.794239 12.302195 5.125638

C 5.622659 11.901658 3.350642

H 6.499680 11.693110 2.731317

C 4.342362 11.827998 2.795864

H 4.210011 11.561530 1.743940

C 3.228352 12.097549 3.602269

H 2.220941 12.040727 3.180552

C 3.401291 12.434884 4.946395

H 2.524324 12.639668 5.569531

N 6.513281 11.694400 11.090847

N 8.077368 15.581213 8.995342

C 8.862039 12.569503 11.616324

C 10.043113 12.500314 12.366627

H 10.112553 11.830153 13.225776

C 11.110888 13.326172 12.012420

H 12.041379 13.294570 12.583896

C 10.969741 14.225673 10.950920

H 11.767504 14.925172 10.694839

C 9.774440 14.217914 10.227929

C 5.358457 13.827145 11.702541

C 4.673009 14.538921 12.690115

H 4.008269 14.021977 13.384469

C 4.869883 15.920772 12.772961

H 4.331260 16.507061 13.520948

C 5.793179 16.540181 11.929118

H 6.008176 17.606816 12.018454

C 6.469589 15.758560 10.984200

C 7.591569 11.876402 12.091173

H 7.212701 12.504537 12.915097

H 7.863034 10.927292 12.574147

C 6.343635 10.241275 10.660117

C 5.844320 9.350593 11.817786

H 5.757516 8.310715 11.468410

H 4.849802 9.658443 12.174155

H 6.531964 9.347966 12.677463

C 9.502900 15.167186 9.074625

H 10.186292 16.029482 9.164529

H 9.757372 14.644499 8.142236

C 7.747780 16.352677 7.718412

C 6.240134 16.664303 7.705936

H 5.990189 17.198161 6.777243

H 5.925100 17.306556 8.540559

H 5.643086 15.741608 7.730814

C 5.248674 12.319396 11.550870

H 4.895321 11.900415 12.509698

H 4.466959 12.104497 10.808142

C 7.662040 16.313524 10.217829

H 7.490196 17.379294 10.015371

H 8.495040 16.302135 10.940653

C 7.698077 9.709126 10.156239

H 7.572966 8.671690 9.813226

H 8.473915 9.699338 10.934937

H 8.069031 10.299362 9.307573

C 5.342134 10.156396 9.494387

H 5.351411 9.134117 9.087740

H 5.614249 10.853016 8.687661

H 4.307836 10.361402 9.807485

C 8.547830 17.669194 7.617433

H 8.262712 18.197878 6.695503

H 9.632156 17.489129 7.567022

H 8.351900 18.354106 8.456475

C 8.056532 15.489119 6.483570

H 7.697193 16.014501 5.586073

H 7.541198 14.521216 6.543703

H 9.133102 15.316654 6.342359

Mn^III^–OH for **3** (1/5)

Mn -0.184309 0.919975 -0.933935

O -2.471267 -1.357747 -1.555092

O -1.499299 0.531222 -2.280634

O -0.586523 -0.455928 0.158093

N 0.445978 2.518683 -2.087633

N 1.960431 0.129675 -1.818496

N 1.242525 1.597129 0.404797

N -1.262545 2.881148 0.039882

C -1.554493 3.605813 -1.220971

C -0.373520 3.583050 -2.170075

C -0.104758 4.609458 -3.076359

C 1.028311 4.507632 -3.887758

C 1.894677 3.423395 -3.737611

C 1.584969 2.438542 -2.795740

C 3.341842 -1.548920 -3.132763

C 1.205996 -0.602394 -4.077536

C 1.128249 -2.221524 -2.182093

C 1.921466 -1.051836 -2.790862

C 2.565314 1.342963 -2.424668

C 2.598356 -0.199836 -0.521006

C 2.377056 0.884428 0.513127

C 3.293681 1.172736 1.525421

C 3.005633 2.212302 2.413711

C 1.849794 2.975411 2.238411

C 0.982972 2.653174 1.190929

C -0.176420 3.548729 0.798814

C -3.214040 3.945285 1.260985

C -2.070634 1.890745 2.183500

C -2.502771 2.624694 0.900779

C -3.490995 1.709206 0.159509

C -3.163827 0.651739 -4.528819

C -4.002840 0.690059 -5.634508

C -4.982631 -0.314277 -5.835904

C -5.074690 -1.355682 -4.877691

C -4.230039 -1.381678 -3.777405

C -3.257643 -0.381987 -3.576483

C -2.373302 -0.441460 -2.384943

H -2.401847 3.112602 -1.716935

H -1.845507 4.655952 -1.048577

H -0.765988 5.475158 -3.136415

H 1.251275 5.289917 -4.616483

H 2.810292 3.348842 -4.326468

H 3.973306 -0.763964 -3.575805

H 3.861936 -1.952654 -2.251245

H 3.272198 -2.362221 -3.870441

H 0.207153 -0.202509 -3.855425

H 1.771519 0.156477 -4.637211

H 1.088212 -1.468795 -4.744615

H 1.632510 -2.669452 -1.313659

H 0.117500 -1.923398 -1.878157

H 1.037709 -3.013959 -2.939545

H 3.260360 1.799047 -1.700242

H 3.188868 1.102149 -3.295794

H 2.147127 -1.126784 -0.142511

H 3.683049 -0.377468 -0.615469

H 4.217741 0.598483 1.608619

H 3.698274 2.446728 3.224951

H 1.629870 3.823288 2.889331

H 0.262996 4.343699 0.173876

H -0.553699 4.068526 1.689377

H -2.547665 4.662479 1.764542

H -3.643985 4.438414 0.376109

H -4.043980 3.733910 1.951528

H -1.520710 0.972261 1.933209

H -1.454986 2.512169 2.849371

H -2.969783 1.609682 2.751417

H -3.846833 2.131636 -0.790791

H -3.054816 0.720987 -0.030473

H -4.377486 1.566275 0.795014

H -2.413994 1.432542 -4.390409

H -3.912768 1.501255 -6.361922

N -5.835161 -0.262084 -6.902418

H -5.821724 -2.142356 -5.013623

H -4.309880 -2.188238 -3.045821

H -1.304384 -0.971907 -0.286715

H -5.631399 0.365347 -7.670495

H -6.387812 -1.077458 -7.136553

Mn^III^–OH_2_ for **3** (2/5)

Mn 5.700218 3.800612 4.656783

O 3.571152 1.301305 4.196087

O 4.277311 3.274820 3.389191

O 5.324320 2.325028 5.698429

N 6.115399 5.378805 3.465360

N 7.863585 3.145432 3.714128

N 7.075580 4.569004 5.941909

N 4.450577 5.615208 5.640511

C 4.050119 6.298793 4.382858

C 5.190599 6.358678 3.390853

C 5.327478 7.377398 2.449407

C 6.434511 7.361765 1.597178

C 7.407021 6.370692 1.738556

C 7.232410 5.386035 2.712443

C 9.383648 1.643259 2.331030

C 7.081786 2.265097 1.517003

C 7.363474 0.680725 3.431760

C 7.931171 1.942992 2.757751

C 8.325575 4.402137 3.065167

C 8.585077 2.907633 4.987997

C 8.283302 3.974484 6.017402

C 9.194695 4.368428 6.996021

C 8.832847 5.386055 7.882251

C 7.603559 6.030723 7.735293

C 6.738468 5.612578 6.723178

C 5.497533 6.394971 6.351113

C 2.457047 6.565161 6.891892

C 3.780926 4.644232 7.849199

C 3.256624 5.290245 6.554659

C 2.319517 4.278265 5.877174

C 2.518735 3.094410 1.192692

C 1.640539 2.965871 0.136348

C 0.790105 1.827980 0.034744

C 0.869405 0.833286 1.051164

C 1.751380 0.971026 2.103448

C 2.598682 2.102728 2.201114

C 3.525160 2.248223 3.299054

H 5.756151 2.124708 6.540500

H 3.222231 5.738861 3.926658

H 3.687216 7.324094 4.560207

H 4.583598 8.173288 2.394301

H 6.554217 8.141086 0.841797

H 8.303374 6.368303 1.116671

H 9.859278 2.480945 1.800229

H 10.018834 1.370224 3.186711

H 9.381630 0.786523 1.641300

H 6.042913 2.498134 1.788287

H 7.482923 3.105274 0.932327

H 7.069773 1.390209 0.851320

H 8.008515 0.304139 4.238620

H 6.356881 0.832871 3.839096

H 7.303517 -0.117874 2.678461

H 9.001299 4.938400 3.752069

H 8.934054 4.202833 2.174489

H 8.271467 1.934290 5.389158

H 9.677666 2.856902 4.849977

H 10.173852 3.890961 7.053408

H 9.524235 5.699320 8.667199

H 7.323227 6.865394 8.379509

H 5.849409 7.209485 5.696878

H 5.101873 6.901507 7.240110

H 3.081367 7.354171 7.337632

H 1.950828 6.983099 6.009243

H 1.677234 6.315833 7.626239

H 4.393601 3.760400 7.626354

H 4.362928 5.334085 8.475954

H 2.923094 4.312548 8.451601

H 1.918468 4.628855 4.916178

H 2.814126 3.310982 5.731051

H 1.456426 4.107575 6.536752

H 3.165853 3.970775 1.256624

H 1.591513 3.738501 -0.634441

N -0.068143 1.696214 -0.997599

H 0.222248 -0.044741 0.989481

H 1.798711 0.198779 2.872822

H 4.249205 1.545875 4.918489

H -0.126085 2.398262 -1.724162

H -0.671672 0.887999 -1.078787

Mn^II^–OH for **3** (0/6)

Mn -0.304545 0.731429 -0.826980

O -2.902608 -1.280307 -1.690365

O -1.763690 0.517808 -2.417199

O -0.828777 -0.809545 0.289812

N 0.433163 2.485688 -2.052881

N 2.043580 0.118274 -1.773451

N 1.331673 1.637418 0.463070

N -1.243323 2.874454 0.144761

C -1.553463 3.551278 -1.134836

C -0.379677 3.548886 -2.101668

C -0.132475 4.597775 -2.993047

C 0.981387 4.506495 -3.833860

C 1.846463 3.416640 -3.720561

C 1.550527 2.420041 -2.779796

C 3.394001 -1.534507 -3.158042

C 1.113697 -0.754068 -3.895298

C 1.343829 -2.282807 -1.930105

C 1.991361 -1.100084 -2.678370

C 2.560880 1.326181 -2.449958

C 2.721514 -0.122411 -0.482407

C 2.509420 1.002222 0.518803

C 3.485825 1.384909 1.444293

C 3.201410 2.445194 2.310698

C 1.992682 3.133062 2.188477

C 1.077390 2.712131 1.213814

C -0.144031 3.554353 0.865923

C -3.128934 3.936107 1.474020

C -1.999179 1.806835 2.252126

C -2.456665 2.620133 1.026489

C -3.495121 1.762903 0.281255

C -3.272638 0.617563 -4.792516

C -4.035741 0.646039 -5.956880

C -5.065586 -0.299478 -6.169235

C -5.289868 -1.270111 -5.165104

C -4.517333 -1.285786 -4.007618

C -3.494083 -0.346018 -3.793388

C -2.666041 -0.388455 -2.530225

H -2.375647 3.005580 -1.617914

H -1.888335 4.595698 -0.994776

H -0.791199 5.467963 -3.023238

H 1.190023 5.299943 -4.555548

H 2.747776 3.349577 -4.333753

H 3.894809 -0.764025 -3.764332

H 4.053874 -1.790783 -2.314764

H 3.304121 -2.433188 -3.787587

H 0.112115 -0.426838 -3.579540

H 1.554366 0.039024 -4.517945

H 1.007234 -1.642896 -4.535463

H 2.040045 -2.753281 -1.218585

H 0.447709 -1.971577 -1.372237

H 1.062531 -3.058852 -2.657870

H 3.307622 1.809221 -1.796572

H 3.118384 1.075637 -3.364184

H 2.308878 -1.042911 -0.046719

H 3.810082 -0.284581 -0.595312

H 4.448389 0.870882 1.480530

H 3.935555 2.754519 3.058528

H 1.771865 3.996875 2.819258

H 0.241760 4.373091 0.234947

H -0.500530 4.055148 1.777476

H -2.447563 4.590674 2.039073

H -3.521025 4.507735 0.618425

H -3.980622 3.709552 2.133355

H -1.554286 0.855634 1.919864

H -1.278164 2.348272 2.882117

H -2.872515 1.582796 2.883765

H -3.928182 2.272772 -0.591482

H -3.065088 0.805698 -0.041829

H -4.327240 1.546397 0.968522

H -2.478778 1.351494 -4.640124

H -3.842580 1.405247 -6.720540

N -5.857724 -0.246832 -7.297601

H -6.080114 -2.013269 -5.307191

H -4.695497 -2.040123 -3.238271

H -1.597201 -1.150668 -0.205506

H -5.491457 0.254532 -8.099216

H -6.370223 -1.085880 -7.545415

Mn^II^–OH_2_ for **3** (1/6)

Mn -0.005355 1.163289 -1.056144

O -2.608646 -1.040852 -0.744529

O -1.782155 0.593021 -2.036306

O -0.553303 -0.254665 0.489605

N 0.614684 2.562037 -2.627919

N 2.017506 0.083574 -2.155634

N 1.825242 1.990120 -0.122010

N -0.647588 3.444979 -0.305007

C -1.087065 3.978113 -1.622633

C -0.083433 3.702607 -2.730500

C 0.139917 4.577732 -3.795917

C 1.106781 4.232433 -4.746431

C 1.863981 3.071580 -4.579607

C 1.605690 2.256197 -3.469906

C 3.047548 -1.868300 -3.433960

C 0.720414 -1.050796 -3.949080

C 1.187532 -2.275014 -1.823138

C 1.759512 -1.261847 -2.833788

C 2.533685 1.104263 -3.105877

C 2.893774 -0.026497 -0.962069

C 2.945778 1.253332 -0.146478

C 4.092856 1.676125 0.529526

C 4.043868 2.885751 1.229521

C 2.890937 3.670879 1.176558

C 1.790419 3.198879 0.450131

C 0.607797 4.104864 0.138568

C -2.215422 4.951952 1.013304

C -1.183050 2.906219 2.071485

C -1.739145 3.504734 0.765638

C -2.952580 2.653011 0.353082

C -3.864618 0.270191 -3.914737

C -4.908879 0.091946 -4.814108

C -5.910360 -0.881025 -4.576779

C -5.808363 -1.665554 -3.401582

C -4.758375 -1.476555 -2.511905

C -3.764964 -0.506843 -2.745567

C -2.649073 -0.313965 -1.773736

H -0.074646 -0.883325 1.045810

H -2.034087 3.489093 -1.891334

H -1.282561 5.063869 -1.590805

H -0.421374 5.510359 -3.876353

H 1.291224 4.887355 -5.601128

H 2.657801 2.813373 -5.283345

H 3.479878 -1.250947 -4.235405

H 3.822066 -2.031297 -2.669256

H 2.813858 -2.848817 -3.875297

H -0.227056 -0.665828 -3.545883

H 1.072031 -0.358656 -4.728270

H 0.517035 -2.012718 -4.442721

H 1.945831 -2.636365 -1.112987

H 0.344691 -1.867386 -1.250252

H 0.824036 -3.155476 -2.372908

H 3.428429 1.578983 -2.668847

H 2.895260 0.641991 -4.033779

H 2.502127 -0.826650 -0.318262

H 3.927386 -0.314068 -1.223980

H 5.004930 1.077375 0.501071

H 4.917895 3.232415 1.785627

H 2.852890 4.646085 1.666001

H 0.962954 4.770163 -0.666111

H 0.435446 4.774808 0.991516

H -1.394872 5.625927 1.302651

H -2.714216 5.378284 0.129949

H -2.946538 4.960085 1.835395

H -0.852756 1.868538 1.919418

H -0.349982 3.486654 2.492612

H -1.981484 2.897122 2.828261

H -3.436317 3.006427 -0.568232

H -2.687458 1.595450 0.227566

H -3.706973 2.712820 1.151650

H -3.100225 1.023939 -4.112348

H -4.965600 0.706790 -5.716633

N -6.967041 -1.030762 -5.437408

H -6.568306 -2.425211 -3.198438

H -4.690434 -2.086851 -1.609157

H -1.400901 -0.696067 0.104263

H -6.895533 -0.633629 -6.366549

H -7.529588 -1.870805 -5.374383

Mn^III^–OH for **4** (1/5)

Mn -0.176966 0.922742 -0.934723

O -2.483416 -1.367738 -1.583235

O -1.507695 0.525427 -2.293775

O -0.579645 -0.452332 0.158035

N 0.441789 2.512144 -2.093149

N 1.959966 0.128405 -1.814993

N 1.243066 1.599463 0.403393

N -1.264609 2.872955 0.032108

C -1.560269 3.598306 -1.228342

C -0.381404 3.574028 -2.179709

C -0.116156 4.596328 -3.091261

C 1.016893 4.494001 -3.902619

C 1.887215 3.413560 -3.747572

C 1.581530 2.432109 -2.801340

C 3.350319 -1.551260 -3.119692

C 1.218528 -0.610899 -4.077227

C 1.134512 -2.226233 -2.177456

C 1.927467 -1.056236 -2.785636

C 2.564306 1.341739 -2.424126

C 2.601525 -0.197529 -0.517532

C 2.377101 0.886676 0.515671

C 3.289368 1.175892 1.531444

C 2.997096 2.215739 2.418047

C 1.841414 2.978367 2.238552

C 0.978721 2.655355 1.188323

C -0.180569 3.547408 0.789805

C -3.222592 3.935995 1.244495

C -2.069586 1.895284 2.184672

C -2.504510 2.616264 0.895603

C -3.486832 1.689610 0.160784

C -3.154076 0.662151 -4.537250

C -3.993835 0.704299 -5.648989

C -4.940528 -0.309414 -5.809232

C -5.072327 -1.358641 -4.896454

C -4.226302 -1.387430 -3.790422

C -3.264740 -0.381058 -3.603991

C -2.368278 -0.443780 -2.392136

H -2.411035 3.108103 -1.721577

H -1.848839 4.648597 -1.054211

H -0.780019 5.459677 -3.155157

H 1.236922 5.273448 -4.635202

H 2.803000 3.339491 -4.336096

H 3.983662 -0.765161 -3.557825

H 3.865482 -1.956349 -2.236040

H 3.285253 -2.363245 -3.859146

H 0.219634 -0.207092 -3.863239

H 1.787877 0.143961 -4.638222

H 1.101021 -1.479992 -4.740674

H 1.634057 -2.668981 -1.303801

H 0.120457 -1.931854 -1.881117

H 1.051421 -3.021889 -2.932243

H 3.255639 1.802233 -1.699061

H 3.191129 1.099080 -3.292198

H 2.155906 -1.126667 -0.137745

H 3.686618 -0.369822 -0.614481

H 4.213293 0.602079 1.618676

H 3.686503 2.450808 3.231808

H 1.618601 3.826213 2.888467

H 0.258567 4.339253 0.160890

H -0.561059 4.071361 1.676318

H -2.558978 4.662333 1.738387

H -3.659063 4.417381 0.356448

H -4.048492 3.726080 1.940214

H -1.510996 0.979428 1.944327

H -1.462143 2.526924 2.848320

H -2.968239 1.610362 2.751327

H -3.838266 2.098348 -0.797131

H -3.047480 0.699473 -0.011378

H -4.376642 1.552564 0.792745

H -2.404375 1.438768 -4.384736

H -3.925929 1.503619 -6.385621

N -5.833486 -0.270574 -6.983706

H -5.824979 -2.128598 -5.060470

H -4.300141 -2.190127 -3.055777

H -1.292121 -0.982320 -0.269629

O -5.702914 0.654641 -7.770946

O -6.655784 -1.165752 -7.106270

Mn^III^–OH_2_ for **4** (2/5)

Mn 0.117829 1.242163 -1.102564

O -2.289038 -0.970828 -0.757507

O -1.483211 0.702799 -2.011017

O -0.321769 0.019781 0.372622

N 0.533137 2.474453 -2.630524

N 1.969088 0.059276 -2.161356

N 1.754687 1.970509 -0.195090

N -0.715399 3.345270 -0.346245

C -1.217603 3.862020 -1.647398

C -0.262497 3.552816 -2.779477

C -0.162111 4.331884 -3.930608

C 0.768178 3.970301 -4.908466

C 1.614720 2.879993 -4.698269

C 1.490248 2.146148 -3.518224

C 3.038431 -1.921829 -3.343891

C 0.772757 -1.066395 -4.043529

C 1.050197 -2.295001 -1.885720

C 1.716068 -1.297219 -2.851341

C 2.484763 1.080446 -3.115198

C 2.865619 -0.055871 -0.984629

C 2.868753 1.207074 -0.155443

C 3.960726 1.608353 0.611198

C 3.882211 2.808146 1.321661

C 2.746258 3.611362 1.206298

C 1.687544 3.176191 0.409712

C 0.523053 4.069248 0.052797

C -2.362145 4.805706 0.916249

C -1.151985 2.936521 2.093304

C -1.786127 3.383563 0.764279

C -2.931102 2.411351 0.440566

C -3.583362 0.492717 -3.834348

C -4.652921 0.367536 -4.718605

C -5.624703 -0.599286 -4.456536

C -5.563755 -1.442297 -3.345122

C -4.490536 -1.308542 -2.468030

C -3.498559 -0.342830 -2.708414

C -2.361517 -0.220909 -1.745799

H 0.187383 -0.360859 1.103793

H -2.184206 3.388377 -1.867238

H -1.390015 4.949610 -1.623060

H -0.797433 5.210373 -4.051304

H 0.852141 4.557618 -5.825082

H 2.378645 2.609965 -5.428782

H 3.597101 -1.265331 -4.027060

H 3.701576 -2.199491 -2.511329

H 2.810786 -2.843664 -3.898683

H -0.154094 -0.565024 -3.733126

H 1.235593 -0.476098 -4.846925

H 0.500984 -2.038676 -4.478464

H 1.677666 -2.540585 -1.017255

H 0.068204 -1.961716 -1.525481

H 0.888007 -3.237078 -2.428806

H 3.327333 1.617874 -2.649868

H 2.908267 0.620046 -4.015780

H 2.521892 -0.889826 -0.357232

H 3.904773 -0.287175 -1.269630

H 4.859652 0.991215 0.638664

H 4.721294 3.132925 1.940137

H 2.686361 4.577927 1.708515

H 0.870861 4.685371 -0.792541

H 0.337829 4.783472 0.864106

H -1.582743 5.564174 1.084440

H -2.957959 5.109104 0.043082

H -3.030184 4.828389 1.789425

H -0.681086 1.948422 2.002899

H -0.413429 3.649656 2.484624

H -1.946043 2.855495 2.849334

H -3.408783 2.604683 -0.529568

H -2.600424 1.366413 0.476355

H -3.711590 2.528393 1.206098

H -2.809036 1.238382 -4.014726

H -4.742279 1.002582 -5.598683

N -6.762564 -0.735602 -5.391432

H -6.345683 -2.182371 -3.181286

H -4.411666 -1.949526 -1.589455

H -1.137535 -0.556556 0.084491

O -6.798117 0.010451 -6.357054

O -7.602238 -1.586616 -5.144961

Mn^II^–OH for **4** (0/6)

Mn -0.303966 0.737460 -0.821509

O -2.922172 -1.297855 -1.722824

O -1.791022 0.519645 -2.423615

O -0.848539 -0.801364 0.282395

N 0.418208 2.480060 -2.060028

N 2.029521 0.114108 -1.774075

N 1.328396 1.640077 0.460806

N -1.245925 2.876248 0.143968

C -1.560973 3.553816 -1.134587

C -0.391869 3.545914 -2.107124

C -0.143791 4.593615 -2.999362

C 0.968578 4.499592 -3.841837

C 1.832266 3.408717 -3.728434

C 1.535756 2.412941 -2.787181

C 3.381615 -1.543585 -3.151855

C 1.104512 -0.764028 -3.896980

C 1.327756 -2.287467 -1.927504

C 1.978241 -1.107110 -2.676497

C 2.546326 1.320158 -2.455366

C 2.713072 -0.123670 -0.484488

C 2.505943 1.003980 0.514284

C 3.485500 1.389116 1.435193

C 3.204584 2.452693 2.298737

C 1.996134 3.141410 2.178249

C 1.077102 2.717847 1.208501

C -0.144605 3.559244 0.860424

C -3.134142 3.940964 1.467135

C -1.994316 1.824200 2.263068

C -2.456893 2.624143 1.030604

C -3.491358 1.754185 0.294703

C -3.265132 0.631493 -4.799417

C -4.013973 0.659733 -5.974694

C -4.996917 -0.315465 -6.164627

C -5.248158 -1.310038 -5.214944

C -4.489058 -1.321371 -4.046819

C -3.493384 -0.355870 -3.827598

C -2.672709 -0.395231 -2.539863

H -2.390235 3.013779 -1.612000

H -1.889392 4.599822 -0.992653

H -0.800334 5.465438 -3.028428

H 1.177762 5.292265 -4.564164

H 2.733403 3.340385 -4.341656

H 3.885518 -0.773823 -3.756424

H 4.038478 -1.801403 -2.306815

H 3.291946 -2.441898 -3.781864

H 0.101817 -0.436675 -3.585982

H 1.546272 0.027427 -4.520711

H 0.999600 -1.654588 -4.534830

H 2.019197 -2.753272 -1.208441

H 0.426604 -1.975712 -1.378439

H 1.053631 -3.067453 -2.653748

H 3.293406 1.805589 -1.804263

H 3.103153 1.066591 -3.369027

H 2.302214 -1.043160 -0.044824

H 3.800809 -0.286483 -0.602116

H 4.447745 0.874498 1.470214

H 3.941474 2.764037 3.042981

H 1.778409 4.007742 2.806566

H 0.240185 4.374879 0.224978

H -0.499343 4.064267 1.770264

H -2.452613 4.605911 2.019747

H -3.535553 4.500286 0.607803

H -3.979856 3.716945 2.134911

H -1.544121 0.872748 1.939643

H -1.277197 2.375033 2.889130

H -2.866594 1.599733 2.895877

H -3.923178 2.249394 -0.586952

H -3.055652 0.793788 -0.010856

H -4.324908 1.544659 0.982290

H -2.488727 1.375236 -4.616907

H -3.850070 1.417466 -6.740020

N -5.791004 -0.295413 -7.403622

H -6.023657 -2.052060 -5.401331

H -4.653717 -2.080651 -3.281140

H -1.611748 -1.159972 -0.205486

O -5.557450 0.582686 -8.222561

O -6.645261 -1.157917 -7.553626

Mn^II^–OH_2_ for **4** (1/6)

Mn 0.016737 1.108014 -0.972189

O -2.723198 -1.033578 -0.782306

O -1.796203 0.593748 -2.017558

O -0.708505 -0.256405 0.567012

N 0.549354 2.472019 -2.614207

N 2.050631 0.054104 -2.129279

N 1.860075 2.008703 -0.140465

N -0.670132 3.382852 -0.274629

C -1.163571 3.865587 -1.593209

C -0.190372 3.585153 -2.725958

C -0.041057 4.430041 -3.828274

C 0.893905 4.082312 -4.808999

C 1.693029 2.950657 -4.638016

C 1.510416 2.167216 -3.490917

C 3.099481 -1.924915 -3.344549

C 0.743441 -1.190753 -3.843634

C 1.283043 -2.302709 -1.674225

C 1.810009 -1.324465 -2.742375

C 2.489541 1.055992 -3.135372

C 2.986894 0.017885 -0.977259

C 3.010231 1.318723 -0.194600

C 4.165624 1.807303 0.420135

C 4.096758 3.032443 1.090189

C 2.911583 3.768786 1.064013

C 1.802286 3.232887 0.398096

C 0.579260 4.090624 0.109405

C -2.222643 4.901035 1.051528

C -1.128728 2.899642 2.131188

C -1.731140 3.454886 0.826900

C -2.943226 2.577122 0.470501

C -3.781243 0.345617 -3.985881

C -4.796513 0.209667 -4.931001

C -5.811333 -0.719866 -4.691702

C -5.838240 -1.513286 -3.542060

C -4.815165 -1.365638 -2.608030

C -3.782843 -0.437863 -2.821007

C -2.684454 -0.288032 -1.789390

H -0.217183 -0.906691 1.087502

H -2.103671 3.343290 -1.818961

H -1.386833 4.946169 -1.584763

H -0.636198 5.340912 -3.914763

H 1.019663 4.712467 -5.692459

H 2.461520 2.690544 -5.368658

H 3.509839 -1.317548 -4.165131

H 3.886580 -2.056068 -2.586755

H 2.877680 -2.919498 -3.759752

H -0.202410 -0.800949 -3.442528

H 1.064603 -0.535718 -4.666474

H 0.546721 -2.181082 -4.280247

H 2.059846 -2.606192 -0.957362

H 0.434780 -1.892160 -1.110276

H 0.937914 -3.220084 -2.173188

H 3.388173 1.571619 -2.756470

H 2.822846 0.573801 -4.064079

H 2.667253 -0.784199 -0.296956

H 4.020252 -0.221807 -1.283498

H 5.099878 1.245526 0.367084

H 4.978146 3.429476 1.598940

H 2.853643 4.756387 1.525755

H 0.883972 4.749484 -0.720880

H 0.415254 4.773039 0.953720

H -1.407332 5.593197 1.310717

H -2.744564 5.300764 0.169162

H -2.937990 4.916599 1.887201

H -0.808512 1.855641 2.004409

H -0.278856 3.490791 2.500699

H -1.898611 2.920697 2.916762

H -3.468305 2.912375 -0.434828

H -2.662083 1.524016 0.347510

H -3.667505 2.631490 1.296808

H -2.974056 1.061925 -4.141741

H -4.813943 0.807610 -5.841217

N -6.890803 -0.867648 -5.685095

H -6.648622 -2.225973 -3.394645

H -4.803085 -1.966842 -1.698282

H -1.525934 -0.701338 0.149746

O -6.852176 -0.162292 -6.682599

O -7.768451 -1.687896 -5.459793

Mn^III^–OH (g) for **1** (1/5)

Mn 5.476782 3.695068 4.658272

O 3.285523 1.363171 4.110749

O 4.148938 3.288454 3.348115

O 5.182058 2.320372 5.755631

N 6.044694 5.335098 3.504861

N 7.633111 2.984512 3.722220

N 6.926313 4.420856 5.972161

N 4.376287 5.629923 5.679320

C 4.035027 6.351496 4.431659

C 5.193535 6.374598 3.451777

C 5.411469 7.415947 2.546991

C 6.527023 7.351959 1.707231

C 7.427261 6.291354 1.828690

C 7.167406 5.291040 2.770564

C 9.028050 1.354208 2.361688

C 6.843938 2.247307 1.474591

C 6.856202 0.611095 3.357881

C 7.601862 1.805962 2.739243

C 8.186373 4.217095 3.113376

C 8.296951 2.659577 5.006358

C 8.071921 3.726508 6.058777

C 8.990423 4.010112 7.072197

C 8.687789 5.022765 7.986262

C 7.514153 5.765150 7.836530

C 6.647576 5.448449 6.786753

C 5.456797 6.318432 6.422554

C 2.418567 6.609159 6.958479

C 3.653897 4.581475 7.828210

C 3.162954 5.316388 6.567513

C 2.190418 4.376080 5.837821

C 2.424933 3.294739 1.138167

C 1.539845 3.269829 0.059044

C 0.609961 2.230847 -0.059132

C 0.566366 1.214976 0.902794

C 1.450140 1.236687 1.981644

C 2.385392 2.277936 2.106842

C 3.314858 2.268753 3.279118

H 3.194545 5.829054 3.953097

H 3.709761 7.391268 4.615274

H 4.723971 8.262952 2.505740

H 6.709405 8.144425 0.977485

H 8.328837 6.247950 1.214343

H 9.629724 2.157197 1.907743

H 9.581240 0.955697 3.225619

H 8.967701 0.544567 1.619590

H 5.840104 2.619091 1.721709

H 7.376454 3.022128 0.903135

H 6.729774 1.384189 0.803122

H 7.390969 0.169531 4.211173

H 5.843199 0.872183 3.686395

H 6.771253 -0.179608 2.598376

H 8.884851 4.688859 3.825972

H 8.795230 3.999706 2.224711

H 7.861187 1.724294 5.384292

H 9.384175 2.498975 4.894220

H 9.924125 3.448821 7.142114

H 9.379496 5.249943 8.800902

H 7.280239 6.588481 8.514454

H 5.863421 7.130534 5.795656

H 5.081418 6.819197 7.325956

H 3.070647 7.347153 7.451974

H 1.943630 7.095007 6.092255

H 1.615592 6.367254 7.670088

H 4.233192 3.686960 7.556450

H 4.255406 5.217576 8.494245

H 2.780480 4.254976 8.411195

H 1.804091 4.789097 4.894915

H 2.646095 3.398482 5.639967

H 1.318172 4.206109 6.485770

H 3.156888 4.097710 1.238341

H 1.573501 4.060679 -0.694372

H -0.082712 2.212263 -0.904331

H -0.159725 0.403942 0.809218

H 1.433433 0.454435 2.742495

H 4.469542 1.749308 5.370714

Mn^III^–OH_2_ (g) for **1** (2/5)

Mn 5.638093 3.802981 4.671190

O 3.490418 1.306196 4.279431

O 4.271326 3.202497 3.378487

O 5.280837 2.346140 5.741593

N 6.074436 5.365071 3.455562

N 7.770324 3.110521 3.749242

N 7.011475 4.582760 5.955741

N 4.404289 5.658207 5.624931

C 4.022908 6.331307 4.356027

C 5.165909 6.359164 3.363349

C 5.320260 7.360300 2.404364

C 6.427063 7.312568 1.551185

C 7.383408 6.307036 1.711234

C 7.192153 5.341893 2.702214

C 9.270351 1.504701 2.459469

C 7.047587 2.229833 1.520470

C 7.140229 0.675811 3.472769

C 7.816979 1.884414 2.806845

C 8.265864 4.343233 3.079633

C 8.489877 2.877997 5.026557

C 8.205649 3.961380 6.047486

C 9.115486 4.344355 7.033453

C 8.766594 5.377937 7.908664

C 7.551269 6.047566 7.743541

C 6.686196 5.638410 6.726423

C 5.454794 6.432647 6.336176

C 2.404279 6.656242 6.839980

C 3.689057 4.735953 7.846128

C 3.188398 5.364835 6.534461

C 2.250966 4.353122 5.858277

C 2.543749 2.924606 1.153652

C 1.662438 2.735353 0.093977

C 0.801122 1.629021 0.090545

C 0.819910 0.708747 1.146566

C 1.698719 0.888564 2.211175

C 2.567783 2.001447 2.220961

C 3.490603 2.194962 3.335748

H 5.551284 2.264402 6.666084

H 3.183978 5.779118 3.908979

H 3.673127 7.364742 4.518444

H 4.590056 8.168754 2.332196

H 6.559046 8.077006 0.781585

H 8.279190 6.281852 1.087430

H 9.832346 2.322906 1.983688

H 9.836667 1.160170 3.337505

H 9.261402 0.671287 1.742075

H 6.018977 2.551620 1.735145

H 7.539296 3.007683 0.918638

H 6.992227 1.334585 0.885047

H 7.685549 0.304576 4.352492

H 6.107337 0.886583 3.772992

H 7.116814 -0.154147 2.752395

H 8.948236 4.878853 3.761904

H 8.876442 4.110554 2.197575

H 8.161398 1.911657 5.434730

H 9.582004 2.808159 4.887052

H 10.084109 3.846247 7.110097

H 9.456436 5.681789 8.699801

H 7.285651 6.891778 8.383217

H 5.819842 7.238999 5.677246

H 5.055872 6.949302 7.218905

H 3.030830 7.447720 7.279579

H 1.905190 7.065319 5.949028

H 1.614462 6.435057 7.572394

H 4.294708 3.840470 7.649721

H 4.268103 5.428186 8.473292

H 2.823038 4.421140 8.445319

H 1.856317 4.696305 4.891343

H 2.737848 3.379408 5.725527

H 1.379178 4.192799 6.508596

H 3.221436 3.779594 1.165234

H 1.641178 3.445525 -0.735260

H 0.110017 1.483076 -0.743257

H 0.146138 -0.150457 1.135933

H 1.719793 0.176301 3.037098

H 4.162919 1.558701 4.999244

Mn^II^–OH (g) for **1** (0/6)

Mn 5.347629 3.520049 4.717862

O 2.916144 1.548085 4.030339

O 3.933180 3.350837 3.157182

O 5.221734 2.041273 5.971975

N 6.058495 5.332589 3.528794

N 7.698266 2.988204 3.752898

N 6.988506 4.485197 5.995400

N 4.397132 5.656161 5.736523

C 4.046713 6.323417 4.466703

C 5.208714 6.362825 3.483417

C 5.412463 7.413495 2.581349

C 6.520634 7.352381 1.730804

C 7.422600 6.291755 1.841311

C 7.168064 5.293047 2.791720

C 9.055621 1.396954 2.312599

C 6.771600 2.188204 1.599957

C 7.009764 0.591350 3.513490

C 7.648225 1.803292 2.805604

C 8.204934 4.221883 3.128419

C 8.321715 2.692827 5.058864

C 8.133425 3.803721 6.077589

C 9.075016 4.112324 7.066493

C 8.776610 5.137620 7.969288

C 7.586439 5.857223 7.836200

C 6.710395 5.509707 6.799119

C 5.488551 6.352448 6.447797

C 2.493332 6.594691 7.118681

C 3.744853 4.507932 7.837045

C 3.218073 5.318732 6.637067

C 2.207003 4.423284 5.900253

C 2.329703 3.243840 0.853474

C 1.508859 3.161407 -0.274678

C 0.545705 2.150814 -0.367947

C 0.407091 1.223195 0.671203

C 1.228238 1.307040 1.797472

C 2.196272 2.317559 1.898565

C 3.072344 2.386213 3.136847

H 3.244396 5.743499 3.988659

H 3.673069 7.356702 4.609806

H 4.723222 8.260248 2.548341

H 6.694414 8.145915 0.999392

H 8.317235 6.248699 1.215348

H 9.547808 2.184965 1.720452

H 9.721812 1.127992 3.147459

H 8.975869 0.511605 1.663225

H 5.765205 2.494586 1.920809

H 7.207919 3.007334 1.007312

H 6.673286 1.323613 0.926373

H 7.717832 0.091494 4.192560

H 6.129162 0.877520 4.107815

H 6.708709 -0.151197 2.759733

H 8.911261 4.709269 3.823307

H 8.799571 4.013522 2.225227

H 7.810730 1.811084 5.473789

H 9.402488 2.460289 4.980605

H 10.017011 3.563048 7.129377

H 9.481437 5.387423 8.766557

H 7.350079 6.681431 8.513536

H 5.865807 7.166718 5.804855

H 5.129743 6.856473 7.358524

H 3.155935 7.279765 7.671365

H 2.045605 7.153709 6.281478

H 1.674173 6.321180 7.800850

H 4.265597 3.600579 7.485305

H 4.420741 5.088496 8.483569

H 2.893843 4.198297 8.462837

H 1.755901 4.908318 5.022296

H 2.651507 3.468573 5.587224

H 1.382683 4.189213 6.590901

H 3.088210 4.023227 0.945857

H 1.619288 3.886785 -1.085921

H -0.096944 2.085700 -1.250515

H -0.344931 0.432281 0.600533

H 1.142373 0.598299 2.623538

H 4.448020 1.519532 5.702896

Mn^II^–OH_2_ (g) for **1** (1/6)

Mn 5.558909 3.784160 4.591847

O 3.393981 1.220362 4.287568

O 4.134105 3.070619 3.272640

O 5.126658 2.086347 5.829618

N 6.102712 5.543374 3.385775

N 7.848042 3.287156 3.688919

N 7.047392 4.719104 5.940261

N 4.403321 5.772265 5.575952

C 4.042668 6.448831 4.302944

C 5.208999 6.539773 3.330904

C 5.380887 7.597239 2.433299

C 6.503746 7.583905 1.599102

C 7.450788 6.564468 1.721870

C 7.226699 5.552954 2.664080

C 9.344984 1.742178 2.321809

C 7.037241 2.375476 1.522001

C 7.315018 0.836536 3.465377

C 7.901924 2.071385 2.758121

C 8.301879 4.532781 3.019297

C 8.558319 3.078262 4.974615

C 8.271520 4.173984 5.990019

C 9.216556 4.631272 6.912451

C 8.854094 5.671007 7.775437

C 7.600190 6.272420 7.649063

C 6.715450 5.782126 6.679925

C 5.443456 6.535282 6.308629

C 2.405408 6.723561 6.829769

C 3.688529 4.746433 7.732957

C 3.191468 5.449593 6.456376

C 2.245674 4.473927 5.736009

C 2.404823 2.656993 1.086908

C 1.530638 2.411163 0.027053

C 0.698949 1.285941 0.053140

C 0.742709 0.406286 1.140848

C 1.616022 0.650007 2.201295

C 2.453587 1.777705 2.181044

C 3.383651 2.028456 3.326396

H 5.280893 1.654351 6.677935

H 3.244607 5.865240 3.821506

H 3.643651 7.466181 4.466153

H 4.658015 8.414437 2.389247

H 6.654547 8.385902 0.872462

H 8.356440 6.565866 1.111339

H 9.822600 2.558002 1.757841

H 9.991722 1.490099 3.176271

H 9.332902 0.864224 1.659107

H 6.000038 2.603410 1.807160

H 7.428510 3.211172 0.923106

H 7.022010 1.494617 0.863699

H 7.974814 0.444752 4.254139

H 6.330011 1.035974 3.906791

H 7.194359 0.028707 2.729355

H 8.997263 5.064663 3.691345

H 8.895891 4.314032 2.120923

H 8.222108 2.119765 5.395779

H 9.653278 2.999096 4.845561

H 10.215281 4.191479 6.950977

H 9.563690 6.035271 8.522256

H 7.322691 7.123891 8.274402

H 5.784121 7.371093 5.673685

H 5.036375 7.019028 7.207858

H 3.029024 7.481867 7.328518

H 1.932980 7.191139 5.952631

H 1.596688 6.464835 7.529007

H 4.266987 3.847096 7.478762

H 4.300096 5.393401 8.378212

H 2.821542 4.429754 8.330753

H 1.837079 4.874023 4.797559

H 2.730736 3.512440 5.526627

H 1.385835 4.270602 6.391147

H 3.060276 3.529406 1.080644

H 1.495456 3.096603 -0.823401

H 0.014500 1.093821 -0.777091

H 0.092938 -0.472001 1.160154

H 1.664544 -0.023583 3.058391

H 4.365373 1.600514 5.288403

Mn^III^–OH (g) for **2** (1/5)

Mn 7.382403 13.088668 9.157646

O 6.165387 12.732933 7.833628

O 8.741175 12.244272 8.283816

H 8.433567 11.867987 7.445221

N 8.748923 13.467177 10.689249

N 6.087893 14.179319 10.369032

C 4.784571 12.824729 7.655119

H 4.340840 13.667067 8.226625

H 4.289952 11.904363 8.037851

C 4.396265 12.985207 6.193655

C 5.204201 12.468557 5.171617

H 6.144682 11.980795 5.434641

C 4.813812 12.578627 3.833724

H 5.453050 12.170798 3.046292

C 3.608833 13.204653 3.500597

H 3.303643 13.289687 2.454806

C 2.797169 13.723558 4.514089

H 1.855568 14.218374 4.262706

C 3.191491 13.616599 5.850677

H 2.551967 14.031110 6.636726

N 6.674880 11.538029 10.963688

N 8.140474 15.399746 8.804630

C 8.748347 12.726730 11.807915

C 9.825171 12.793080 12.696420

H 9.833583 12.175308 13.596671

C 10.873425 13.673025 12.419811

H 11.726898 13.740611 13.098566

C 10.811515 14.487649 11.287088

H 11.597121 15.214618 11.072472

C 9.720189 14.356598 10.424932

C 5.158441 13.507506 11.074396

C 4.160926 14.198925 11.765506

H 3.410487 13.651865 12.339362

C 4.150568 15.595086 11.712412

H 3.367969 16.158187 12.226425

C 5.168899 16.267826 11.034671

H 5.211697 17.358829 11.027050

C 6.157826 15.522014 10.385713

C 7.487851 11.943694 12.130801

H 6.884514 12.603348 12.778542

H 7.749145 11.086556 12.767599

C 6.816039 10.048605 10.628596

C 6.202101 9.165735 11.733830

H 6.350255 8.106081 11.478352

H 5.117225 9.321550 11.838891

H 6.669545 9.323470 12.718524

C 9.558838 15.193239 9.172498

H 10.102885 16.144284 9.314961

H 10.047629 14.653645 8.349584

C 7.961110 15.971600 7.392573

C 6.460320 16.177244 7.126742

H 6.319650 16.486353 6.081363

H 6.010014 16.962179 7.751935

H 5.908898 15.239677 7.267450

C 5.276620 11.994007 11.097122

H 4.790881 11.623242 12.018579

H 4.697961 11.589220 10.255172

C 7.416504 16.173801 9.835093

H 7.178964 17.192962 9.498425

H 8.074621 16.312681 10.710556

C 8.311881 9.726502 10.462310

H 8.419451 8.677568 10.149851

H 8.880401 9.834263 11.398143

H 8.764949 10.366985 9.691567

C 6.114400 9.741169 9.294576

H 6.338972 8.703845 9.007175

H 6.462977 10.405334 8.492811

H 5.018397 9.813698 9.360215

C 8.706872 17.312799 7.239205

H 8.485091 17.743747 6.251809

H 9.799139 17.192750 7.298642

H 8.401281 18.059068 7.990106

C 8.485100 14.973656 6.346446

H 8.326612 15.399766 5.344976

H 7.938560 14.024442 6.401484

H 9.561705 14.772472 6.440052

Mn^III^–OH_2_ (g) for **2** (2/5)

Mn 7.274912 13.254904 9.229934

O 6.104475 12.961477 7.922594

O 8.659644 12.347931 7.873323

H 9.500802 11.870582 7.935828

H 8.203515 12.071598 7.059223

N 8.778250 13.467587 10.594080

N 6.067673 14.254950 10.546279

C 4.709046 12.988792 7.627985

H 4.270234 13.948387 7.951792

H 4.204931 12.180991 8.187812

C 4.501756 12.794604 6.144194

C 4.685794 11.525771 5.567105

H 4.947858 10.672170 6.199054

C 4.509099 11.338044 4.195291

H 4.641766 10.345673 3.757953

C 4.142838 12.417486 3.382749

H 3.995506 12.269238 2.310472

C 3.951536 13.682027 3.947588

H 3.649870 14.522404 3.318081

C 4.135307 13.870056 5.320438

H 3.970955 14.859323 5.756319

N 6.675966 11.582196 10.877226

N 8.036518 15.519733 8.920573

C 8.824254 12.668104 11.680149

C 9.943399 12.687353 12.515495

H 9.991432 12.027957 13.384545

C 10.981145 13.580599 12.237180

H 11.865343 13.610821 12.878612

C 10.864909 14.465571 11.160699

H 11.636555 15.212116 10.962290

C 9.734954 14.388917 10.345829

C 5.169086 13.540284 11.248804

C 4.211887 14.195535 12.024693

H 3.481238 13.623357 12.600104

C 4.215367 15.594132 12.054734

H 3.463570 16.130113 12.639209

C 5.206847 16.304183 11.371086

H 5.257509 17.393355 11.431667

C 6.154510 15.594272 10.630368

C 7.569000 11.896428 12.027759

H 7.020939 12.529521 12.746449

H 7.834345 10.994438 12.595031

C 6.749426 10.088922 10.482978

C 6.178525 9.184422 11.592803

H 6.291604 8.132306 11.293336

H 5.104303 9.352197 11.759324

H 6.699875 9.295405 12.555829

C 9.486771 15.324514 9.181260

H 10.007826 16.276215 9.379373

H 9.946397 14.902333 8.275818

C 7.775572 16.214743 7.561226

C 6.273023 16.515755 7.440077

H 6.059701 16.853894 6.416339

H 5.932105 17.316587 8.111153

H 5.672099 15.615722 7.621813

C 5.286777 12.029111 11.164335

H 4.897814 11.597319 12.101990

H 4.631691 11.666618 10.360076

C 7.397808 16.247334 10.051151

H 7.179573 17.291588 9.792459

H 8.112411 16.306345 10.889852

C 8.219988 9.722558 10.221814

H 8.277188 8.676462 9.889509

H 8.858449 9.803689 11.112752

H 8.647487 10.345466 9.423863

C 5.959636 9.849247 9.186852

H 6.121173 8.812390 8.859054

H 6.287182 10.518660 8.380490

H 4.873748 9.963131 9.317497

C 8.577762 17.525692 7.450889

H 8.271845 18.059274 6.539321

H 9.661064 17.353495 7.368391

H 8.398679 18.210668 8.294563

C 8.164587 15.275747 6.408558

H 8.019076 15.807709 5.457430

H 7.522673 14.385355 6.389456

H 9.220460 14.968991 6.432887

Mn^II^–OH (g) for **2** (0/6)

Mn 7.501416 13.030115 8.853259

O 5.938542 12.799666 7.624246

O 9.024547 12.194063 7.919452

H 8.726862 11.839595 7.069474

N 8.762918 13.512218 10.816442

N 6.224335 14.270993 10.405317

C 4.638806 13.199088 7.507759

H 4.492274 14.290044 7.720336

H 3.959210 12.691930 8.245066

C 4.032598 12.939556 6.128413

C 4.784685 12.303861 5.133594

H 5.806295 12.011581 5.384660

C 4.235333 12.059463 3.869575

H 4.836171 11.561474 3.102562

C 2.923853 12.448035 3.582859

H 2.493859 12.257688 2.595548

C 2.163524 13.084719 4.571926

H 1.136062 13.393586 4.357570

C 2.715848 13.326687 5.831519

H 2.114961 13.826023 6.600093

N 6.780679 11.430538 10.774052

N 8.250032 15.518758 8.773140

C 8.579299 12.808983 11.933718

C 9.143753 13.220857 13.151295

H 8.989385 12.630297 14.057456

C 9.885749 14.399662 13.181831

H 10.337869 14.744652 14.115277

C 10.012152 15.155912 12.013760

H 10.549186 16.106809 12.013820

C 9.420688 14.678914 10.839065

C 5.577804 13.565003 11.342956

C 5.001279 14.195247 12.451016

H 4.471343 13.609259 13.204951

C 5.135990 15.580320 12.579004

H 4.696131 16.101088 13.433418

C 5.872550 16.284547 11.627987

H 6.034957 17.360371 11.727319

C 6.420436 15.583036 10.542984

C 7.765196 11.524354 11.860558

H 7.297976 11.360792 12.852405

H 8.494854 10.712746 11.737376

C 6.636418 10.002915 10.243821

C 6.206763 9.026969 11.360652

H 6.090006 8.016158 10.940364

H 5.237962 9.305421 11.806179

H 6.950578 8.955983 12.169948

C 9.511423 15.440786 9.530653

H 9.931126 16.444895 9.741580

H 10.240325 14.902757 8.910821

C 8.451255 15.969995 7.325021

C 7.162120 15.692443 6.531233

H 7.322720 15.959086 5.475851

H 6.304440 16.291010 6.875546

H 6.880679 14.629666 6.589398

C 5.499596 12.060809 11.140255

H 5.061702 11.611972 12.054329

H 4.790630 11.887386 10.319901

C 7.231849 16.311677 9.477525

H 6.505021 16.673977 8.737091

H 7.666250 17.218973 9.943026

C 7.987487 9.556143 9.648367

H 7.845194 8.608907 9.106464

H 8.750107 9.357591 10.418251

H 8.391303 10.312677 8.953037

C 5.589381 9.964357 9.115141

H 5.644896 8.986795 8.613876

H 5.770272 10.756318 8.369916

H 4.559275 10.062812 9.491450

C 8.801899 17.471738 7.244833

H 9.007337 17.745305 6.198490

H 9.704796 17.718124 7.826657

H 7.980924 18.118552 7.592493

C 9.584406 15.149479 6.676605

H 9.528387 15.268662 5.584266

H 9.491815 14.077656 6.926378

H 10.583418 15.500823 6.979526

Mn^II^–OH_2_ (g) for **2** (1/6)

Mn 7.046553 13.450313 9.143599

O 6.009662 13.119919 7.516247

O 8.427329 12.439386 7.471940

H 8.842000 11.589941 7.272886

H 7.525509 12.448499 7.046510

N 8.766047 13.341745 10.514257

N 6.215616 14.557155 10.859883

C 4.772569 13.282915 6.881975

H 4.604261 14.351976 6.627194

H 3.933034 12.995195 7.549335

C 4.672162 12.464253 5.607074

C 5.537467 12.724409 4.530774

H 6.264945 13.537585 4.610604

C 5.470703 11.968258 3.358925

H 6.146509 12.188288 2.528229

C 4.531664 10.936527 3.241574

H 4.475238 10.347116 2.323055

C 3.663011 10.670109 4.302307

H 2.924011 9.869179 4.217092

C 3.737015 11.428662 5.476275

H 3.052159 11.214393 6.302447

N 6.397355 11.772218 10.930381

N 8.184982 15.665679 9.087836

C 8.785667 12.480584 11.535873

C 9.954644 12.300364 12.286626

H 9.982388 11.578768 13.106167

C 11.071243 13.081887 11.983908

H 11.995089 12.962198 12.554992

C 10.990600 14.047390 10.974912

H 11.832538 14.709243 10.761985

C 9.801879 14.151983 10.248013

C 5.347586 13.921969 11.662393

C 4.692407 14.608187 12.687779

H 3.988694 14.089943 13.342308

C 4.966875 15.969103 12.859279

H 4.453079 16.537947 13.637936

C 5.932317 16.586385 12.062143

H 6.202633 17.632768 12.220278

C 6.572946 15.828390 11.073494

C 7.469247 11.833261 11.952516

H 7.112417 12.429070 12.810501

H 7.681181 10.840808 12.375028

C 6.164536 10.354501 10.404894

C 5.674816 9.397851 11.511619

H 5.516170 8.396628 11.084234

H 4.713148 9.719027 11.940331

H 6.396342 9.281294 12.334935

C 9.585928 15.178026 9.147259

H 10.314177 15.998326 9.282908

H 9.815786 14.694239 8.187216

C 7.875655 16.499502 7.841631

C 6.370866 16.821093 7.848633

H 6.110079 17.373760 6.934431

H 6.065448 17.449043 8.698374

H 5.776373 15.895792 7.861523

C 5.158675 12.435977 11.403780

H 4.753124 11.971053 12.321318

H 4.388846 12.316270 10.627591

C 7.797490 16.360561 10.338837

H 7.674167 17.441123 10.180358

H 8.625331 16.279263 11.064333

C 7.491374 9.841752 9.818215

H 7.340900 8.839804 9.390857

H 8.290337 9.753523 10.569080

H 7.841295 10.506073 9.015518

C 5.125187 10.378610 9.270944

H 5.103198 9.392747 8.784076

H 5.382597 11.128815 8.509454

H 4.103875 10.568331 9.633526

C 8.697619 17.804650 7.803275

H 8.432632 18.376007 6.901394

H 9.780005 17.610430 7.753207

H 8.506678 18.464098 8.663896

C 8.176183 15.690122 6.569118

H 7.837783 16.268385 5.696853

H 7.639820 14.732674 6.571162

H 9.249167 15.499523 6.423805

Mn^III^–OH (g) for **3** (1/5)

Mn -0.194153 0.927466 -0.957161

O -2.439648 -1.320623 -1.547753

O -1.487841 0.569180 -2.299960

O -0.568267 -0.433123 0.132429

N 0.469688 2.549009 -2.093297

N 1.967660 0.143869 -1.829559

N 1.244024 1.607887 0.398230

N -1.252176 2.906834 0.031637

C -1.530801 3.634105 -1.227577

C -0.341980 3.617706 -2.170672

C -0.059529 4.651675 -3.066217

C 1.078359 4.548796 -3.871416

C 1.935765 3.456442 -3.724868

C 1.611395 2.465320 -2.793054

C 3.337849 -1.536309 -3.152552

C 1.209513 -0.567118 -4.095330

C 1.115457 -2.200381 -2.212676

C 1.919359 -1.033602 -2.811932

C 2.580938 1.354871 -2.422319

C 2.579069 -0.202487 -0.525727

C 2.361779 0.874427 0.518320

C 3.260913 1.128061 1.556865

C 2.968453 2.152897 2.460537

C 1.826275 2.935423 2.276558

C 0.979001 2.646488 1.203081

C -0.170329 3.557135 0.805296

C -3.209243 3.953962 1.255968

C -2.072921 1.882126 2.155750

C -2.500739 2.635732 0.883072

C -3.485399 1.732097 0.123769

C -3.164345 0.618364 -4.557210

C -4.014963 0.625418 -5.653661

C -4.998481 -0.381789 -5.810692

C -5.083726 -1.392451 -4.821164

C -4.227937 -1.389528 -3.731835

C -3.251283 -0.385570 -3.573930

C -2.363456 -0.423310 -2.391019

H -2.370337 3.134589 -1.731670

H -1.829853 4.684211 -1.057562

H -0.714923 5.522638 -3.126634

H 1.311432 5.335115 -4.593362

H 2.853532 3.381098 -4.311565

H 3.978073 -0.756454 -3.594036

H 3.855332 -1.949640 -2.273514

H 3.267962 -2.346697 -3.893042

H 0.212962 -0.161646 -3.872688

H 1.782939 0.188951 -4.652321

H 1.083179 -1.426187 -4.769862

H 1.612663 -2.660981 -1.346729

H 0.104683 -1.902394 -1.909540

H 1.020885 -2.987463 -2.974865

H 3.276346 1.801749 -1.690842

H 3.205748 1.115567 -3.294294

H 2.096343 -1.119415 -0.160016

H 3.662567 -0.404413 -0.603602

H 4.171967 0.534499 1.653838

H 3.644450 2.357696 3.294091

H 1.602178 3.768014 2.946457

H 0.282025 4.354555 0.190929

H -0.553153 4.070863 1.698353

H -2.546196 4.666427 1.771971

H -3.638956 4.459145 0.377198

H -4.042176 3.739791 1.941595

H -1.520919 0.966394 1.898002

H -1.465757 2.494654 2.838699

H -2.973801 1.588975 2.714213

H -3.831419 2.161178 -0.827525

H -3.059554 0.739794 -0.067009

H -4.380098 1.591097 0.747717

H -2.409132 1.399456 -4.452720

H -3.931067 1.413157 -6.407439

N -5.853909 -0.369386 -6.874168

H -5.835568 -2.179663 -4.923202

H -4.297442 -2.170908 -2.972714

H -1.292889 -0.972622 -0.281618

H -5.715214 0.278317 -7.637181

H -6.472988 -1.150560 -7.041176

Mn^III^–OH_2_ (g) for **3** (2/5)

Mn 5.695076 3.809309 4.652178

O 3.564310 1.316023 4.223900

O 4.343237 3.246380 3.370459

O 5.376732 2.331397 5.703918

N 6.119395 5.411853 3.471371

N 7.830387 3.159130 3.693275

N 7.079050 4.574598 5.940555

N 4.463293 5.645366 5.663009

C 4.069853 6.342343 4.411822

C 5.207070 6.403801 3.413606

C 5.351027 7.433707 2.483544

C 6.452237 7.415388 1.622213

C 7.412576 6.408861 1.746109

C 7.230867 5.414746 2.710377

C 9.320507 1.589965 2.351276

C 7.084623 2.330277 1.454223

C 7.202480 0.731175 3.367058

C 7.869910 1.957028 2.723242

C 8.312250 4.409528 3.049847

C 8.558598 2.900120 4.959522

C 8.278234 3.959701 6.006119

C 9.197307 4.326746 6.989733

C 8.852472 5.338340 7.891542

C 7.631222 6.003034 7.754799

C 6.757173 5.610679 6.738440

C 5.515977 6.406569 6.383078

C 2.470487 6.600015 6.921788

C 3.772403 4.657298 7.861211

C 3.257658 5.321516 6.572591

C 2.318433 4.324802 5.876376

C 2.594644 3.014666 1.136831

C 1.724149 2.855182 0.081695

C 0.849984 1.729277 0.026432

C 0.902143 0.778852 1.087348

C 1.775883 0.945838 2.137550

C 2.647099 2.068264 2.193711

C 3.554064 2.231576 3.292866

H 5.650412 2.253779 6.627478

H 3.235704 5.789983 3.956383

H 3.710494 7.368716 4.597996

H 4.617541 8.241217 2.440408

H 6.576798 8.202858 0.874974

H 8.304190 6.404764 1.115895

H 9.874722 2.421563 1.889601

H 9.897072 1.227388 3.215336

H 9.307069 0.773540 1.614660

H 6.057962 2.642586 1.690671

H 7.567171 3.124412 0.866201

H 7.025124 1.450128 0.798383

H 7.756883 0.342051 4.233291

H 6.171636 0.931907 3.680600

H 7.175696 -0.081918 2.627749

H 9.001027 4.930510 3.737056

H 8.912965 4.202662 2.154461

H 8.233176 1.925019 5.348753

H 9.650295 2.835400 4.812607

H 10.169657 3.833083 7.044057

H 9.549346 5.629090 8.681386

H 7.367096 6.830860 8.416100

H 5.868626 7.233135 5.742619

H 5.124429 6.896698 7.284225

H 3.096901 7.380268 7.381313

H 1.965763 7.033883 6.045803

H 1.685198 6.354383 7.651304

H 4.376252 3.768743 7.631520

H 4.358260 5.332275 8.500852

H 2.913354 4.324627 8.460772

H 1.908510 4.696748 4.926668

H 2.809976 3.360131 5.702160

H 1.457120 4.136754 6.533273

H 3.259727 3.879617 1.161836

H 1.697386 3.591108 -0.725333

N -0.003140 1.567209 -1.001988

H 0.239697 -0.089445 1.060670

H 1.802039 0.207511 2.940320

H 4.235201 1.552620 4.940358

H -0.047754 2.234723 -1.761848

H -0.625019 0.769231 -1.048320

Mn^II^–OH (g) for **3** (0/6)

Mn -0.330006 0.761143 -0.893205

O -2.801232 -1.101954 -1.623366

O -1.700373 0.654842 -2.486939

O -0.521147 -0.710489 0.363477

N 0.480589 2.550963 -2.059163

N 2.027347 0.148101 -1.799853

N 1.315587 1.669315 0.425604

N -1.224750 2.932714 0.105207

C -1.519705 3.609618 -1.173359

C -0.332112 3.609318 -2.126155

C -0.070061 4.651215 -3.023466

C 1.055852 4.550669 -3.846542

C 1.917168 3.459151 -3.714191

C 1.604915 2.471190 -2.769685

C 3.358140 -1.489538 -3.211778

C 1.121211 -0.613746 -3.974839

C 1.257250 -2.221612 -2.060356

C 1.955441 -1.032482 -2.750363

C 2.593914 1.363094 -2.408849

C 2.604145 -0.171358 -0.478467

C 2.431737 0.945694 0.536270

C 3.358282 1.218417 1.549685

C 3.074819 2.253396 2.446267

C 1.915768 3.016460 2.283931

C 1.054631 2.702387 1.223835

C -0.126709 3.589093 0.843208

C -3.124045 3.938547 1.445402

C -1.968272 1.804868 2.185801

C -2.436360 2.637338 0.976317

C -3.463609 1.783285 0.213128

C -3.271357 0.590694 -4.823932

C -4.069930 0.539354 -5.964395

C -5.089190 -0.427707 -6.084802

C -5.273784 -1.337296 -5.022144

C -4.468930 -1.276561 -3.888778

C -3.454325 -0.313926 -3.766927

C -2.601999 -0.276211 -2.522446

H -2.326367 3.054240 -1.672768

H -1.863518 4.654730 -1.040624

H -0.728757 5.521124 -3.073904

H 1.274967 5.336926 -4.573678

H 2.824374 3.383849 -4.318715

H 3.891935 -0.719631 -3.791629

H 3.994881 -1.782457 -2.362078

H 3.261290 -2.371313 -3.863749

H 0.119478 -0.271008 -3.676256

H 1.601201 0.189851 -4.555010

H 1.007024 -1.473133 -4.652670

H 1.930772 -2.747050 -1.365591

H 0.373463 -1.904975 -1.486596

H 0.947922 -2.951820 -2.822792

H 3.299840 1.823645 -1.695463

H 3.203035 1.134182 -3.297365

H 2.047152 -1.031565 -0.077658

H 3.676828 -0.446000 -0.528094

H 4.277334 0.634493 1.635929

H 3.767564 2.476281 3.261926

H 1.692299 3.848041 2.956659

H 0.295316 4.389108 0.210246

H -0.489037 4.105804 1.745441

H -2.450087 4.596363 2.017185

H -3.529395 4.516815 0.599676

H -3.969693 3.694020 2.105948

H -1.472107 0.880020 1.843878

H -1.287012 2.359134 2.849686

H -2.844614 1.525177 2.790415

H -3.876186 2.288706 -0.672250

H -3.050245 0.813185 -0.095583

H -4.311480 1.578565 0.884398

H -2.481354 1.338828 -4.734208

H -3.912112 1.255003 -6.777640

N -5.920726 -0.453717 -7.197467

H -6.061107 -2.094148 -5.095731

H -4.606487 -1.979240 -3.064234

H -1.308984 -1.200440 0.075362

H -5.557103 -0.023152 -8.039795

H -6.401014 -1.327931 -7.377234

Mn^II^–OH_2_ (g) for **3** (1/6)

Mn 0.006729 1.153856 -1.095570

O -2.569302 -0.945527 -0.698034

O -1.693261 0.562393 -2.100516

O -0.512058 -0.236586 0.434178

N 0.659141 2.596252 -2.631154

N 2.023149 0.113948 -2.181855

N 1.823275 1.972148 -0.115856

N -0.629850 3.446775 -0.315968

C -1.059600 3.991272 -1.629665

C -0.052333 3.726050 -2.737880

C 0.158730 4.597896 -3.809835

C 1.125453 4.257411 -4.761856

C 1.894323 3.103724 -4.592020

C 1.646803 2.292896 -3.477179

C 3.007653 -1.861559 -3.455234

C 0.744726 -0.915747 -4.049023

C 1.054870 -2.196385 -1.930809

C 1.725953 -1.205786 -2.899411

C 2.574695 1.145047 -3.096371

C 2.870376 -0.045712 -0.975173

C 2.929900 1.215488 -0.126682

C 4.066026 1.606870 0.587103

C 4.018538 2.805631 1.306678

C 2.880286 3.611579 1.237937

C 1.790562 3.169972 0.476270

C 0.622409 4.092310 0.147121

C -2.222895 4.924293 1.005043

C -1.174733 2.878922 2.049087

C -1.731698 3.484445 0.747351

C -2.928564 2.619737 0.316670

C -3.803095 0.175344 -3.944011

C -4.856827 -0.038182 -4.821301

C -5.879968 -0.965488 -4.507512

C -5.793176 -1.666096 -3.279810

C -4.734884 -1.444556 -2.412152

C -3.718191 -0.520620 -2.723617

C -2.599878 -0.296650 -1.780295

H -0.311764 -0.570924 1.316004

H -2.004054 3.501380 -1.907617

H -1.258446 5.077628 -1.590318

H -0.414657 5.523040 -3.897314

H 1.298712 4.908482 -5.622133

H 2.684990 2.848879 -5.301184

H 3.522303 -1.238745 -4.202964

H 3.727579 -2.109544 -2.660033

H 2.745402 -2.805371 -3.955874

H -0.183363 -0.459399 -3.675241

H 1.174343 -0.258583 -4.819727

H 0.481644 -1.859613 -4.548519

H 1.754081 -2.596113 -1.180867

H 0.200128 -1.752780 -1.404043

H 0.684488 -3.058536 -2.503912

H 3.444333 1.621857 -2.612031

H 2.979345 0.695436 -4.014003

H 2.440509 -0.852277 -0.363806

H 3.904178 -0.351652 -1.220049

H 4.969675 0.993962 0.575311

H 4.883109 3.127351 1.892336

H 2.847591 4.577919 1.746088

H 0.998795 4.752613 -0.653012

H 0.444151 4.763719 0.999100

H -1.411539 5.608802 1.298173

H -2.731626 5.351674 0.127672

H -2.952188 4.922102 1.828496

H -0.808632 1.857482 1.873926

H -0.367633 3.475776 2.497942

H -1.981335 2.823176 2.794522

H -3.402046 2.961876 -0.614168

H -2.652566 1.564175 0.202535

H -3.699556 2.673832 1.099361

H -3.019611 0.892173 -4.196722

H -4.905182 0.512790 -5.764716

N -6.936149 -1.160559 -5.353492

H -6.572984 -2.386600 -3.018273

H -4.674396 -1.988009 -1.467573

H -1.435550 -0.632327 0.062110

H -6.914482 -0.776286 -6.288069

H -7.577513 -1.925213 -5.192774

Mn^III^–OH (g) for **4** (1/5)

Mn -0.191656 0.932150 -0.953917

O -2.459007 -1.330298 -1.566879

O -1.504847 0.562956 -2.305573

O -0.565226 -0.428424 0.137156

N 0.458053 2.545071 -2.095522

N 1.959262 0.142237 -1.830804

N 1.244632 1.608541 0.396409

N -1.253745 2.902339 0.033835

C -1.537325 3.635179 -1.221920

C -0.354726 3.613963 -2.172676

C -0.077518 4.644011 -3.073986

C 1.056339 4.538630 -3.884725

C 1.916088 3.448092 -3.737198

C 1.597826 2.460257 -2.800209

C 3.338691 -1.537394 -3.148535

C 1.210599 -0.577846 -4.098121

C 1.117880 -2.206635 -2.209978

C 1.918131 -1.038705 -2.811548

C 2.570323 1.353924 -2.427887

C 2.579851 -0.200915 -0.529327

C 2.362663 0.874859 0.515384

C 3.261789 1.128099 1.553763

C 2.969335 2.152261 2.458281

C 1.826865 2.934841 2.275380

C 0.979138 2.646574 1.202465

C -0.170134 3.556055 0.804591

C -3.210681 3.954365 1.256939

C -2.069832 1.890355 2.167965

C -2.500908 2.635117 0.891269

C -3.484889 1.725253 0.138886

C -3.151304 0.619992 -4.567742

C -3.997021 0.629046 -5.675634

C -4.948849 -0.383660 -5.795837

C -5.080357 -1.400802 -4.849718

C -4.228240 -1.399216 -3.746970

C -3.261316 -0.390942 -3.598871

C -2.365461 -0.426199 -2.392840

H -2.386081 3.145611 -1.720188

H -1.827588 4.686421 -1.045880

H -0.733561 5.514476 -3.134798

H 1.284707 5.321888 -4.611458

H 2.831389 3.372031 -4.327604

H 3.977748 -0.756268 -3.589156

H 3.855382 -1.949745 -2.268761

H 3.272262 -2.347972 -3.889069

H 0.213791 -0.170900 -3.879865

H 1.783946 0.176312 -4.657382

H 1.086211 -1.439364 -4.769807

H 1.616495 -2.664730 -1.343653

H 0.107092 -1.910356 -1.905342

H 1.024120 -2.995617 -2.970193

H 3.265223 1.804166 -1.698116

H 3.194950 1.112926 -3.299238

H 2.106143 -1.121576 -0.161341

H 3.663728 -0.395809 -0.614453

H 4.173071 0.534795 1.650153

H 3.645665 2.356657 3.291638

H 1.603226 3.767102 2.945811

H 0.280671 4.351812 0.187105

H -0.552709 4.071954 1.696207

H -2.546964 4.672239 1.764273

H -3.646620 4.451943 0.377008

H -4.039341 3.743284 1.948588

H -1.513948 0.975149 1.917235

H -1.465732 2.509284 2.847552

H -2.969529 1.597039 2.728140

H -3.831266 2.147756 -0.815386

H -3.057194 0.731835 -0.042235

H -4.379816 1.587766 0.763084

H -2.394374 1.395552 -4.446616

H -3.937597 1.397209 -6.446114

N -5.852316 -0.376700 -6.974097

H -5.840420 -2.168720 -4.992052

H -4.297664 -2.176390 -2.984335

H -1.284533 -0.980361 -0.257675

O -5.712026 0.528606 -7.778396

O -6.670424 -1.274939 -7.055191

Mn^III^–OH_2_ (g) for **4** (2/5)

Mn 0.121917 1.242919 -1.097837

O -2.380456 -0.913912 -0.743476

O -1.523084 0.676427 -2.065758

O -0.259967 0.035525 0.242732

N 0.556078 2.512492 -2.611511

N 1.954317 0.083580 -2.176704

N 1.780441 1.969086 -0.170608

N -0.674523 3.388535 -0.310808

C -1.164582 3.922783 -1.608531

C -0.210689 3.616260 -2.743634

C -0.082648 4.424757 -3.872983

C 0.849724 4.071917 -4.853624

C 1.673664 2.960570 -4.659567

C 1.520464 2.197514 -3.500221

C 2.991783 -1.915211 -3.370579

C 0.764337 -0.991523 -4.099089

C 0.965244 -2.233820 -1.941785

C 1.680029 -1.260965 -2.892902

C 2.496203 1.107443 -3.111299

C 2.838371 -0.070286 -0.993714

C 2.876737 1.182873 -0.142374

C 3.979320 1.554421 0.627491

C 3.923206 2.747957 1.354514

C 2.800574 3.573610 1.249598

C 1.733000 3.164887 0.447600

C 0.574828 4.080675 0.102968

C -2.292621 4.866965 0.981778

C -1.133516 2.937510 2.115567

C -1.752838 3.435011 0.798265

C -2.917577 2.497906 0.445099

C -3.616695 0.335755 -3.933311

C -4.677847 0.135240 -4.809993

C -5.650045 -0.807872 -4.472889

C -5.600801 -1.556669 -3.297361

C -4.536131 -1.353370 -2.422760

C -3.540171 -0.405661 -2.736836

C -2.418326 -0.189487 -1.813832

H 0.146983 0.034513 1.119772

H -2.136337 3.458987 -1.830450

H -1.333649 5.012237 -1.575828

H -0.697155 5.320972 -3.979140

H 0.954697 4.681466 -5.754505

H 2.441702 2.698819 -5.390217

H 3.574167 -1.276623 -4.052172

H 3.642124 -2.213895 -2.535072

H 2.752266 -2.832437 -3.927949

H -0.161667 -0.479908 -3.801348

H 1.251340 -0.400874 -4.888277

H 0.484487 -1.950509 -4.557713

H 1.592568 -2.553163 -1.097092

H 0.033960 -1.818660 -1.539330

H 0.711854 -3.146916 -2.498883

H 3.347598 1.622076 -2.633548

H 2.916593 0.650931 -4.016697

H 2.455838 -0.901436 -0.384447

H 3.872315 -0.333367 -1.274093

H 4.869958 0.923193 0.651802

H 4.768324 3.049212 1.978359

H 2.761694 4.534257 1.767406

H 0.933193 4.710492 -0.729170

H 0.400194 4.780280 0.930718

H -1.500369 5.604264 1.183974

H -2.872686 5.212080 0.113119

H -2.972428 4.888950 1.845804

H -0.694290 1.937593 1.994624

H -0.371936 3.614471 2.527155

H -1.924178 2.858516 2.875009

H -3.413926 2.750536 -0.502804

H -2.596248 1.449498 0.419237

H -3.685417 2.581183 1.227452

H -2.839364 1.063096 -4.170427

H -4.773775 0.686158 -5.745577

N -6.790030 -1.022328 -5.413533

H -6.390014 -2.279596 -3.089294

H -4.472831 -1.925405 -1.496285

H -1.565789 -0.663827 -0.179973

O -6.796308 -0.347339 -6.424902

O -7.616010 -1.851996 -5.090843

Mn^II^–OH (g) for **4** (0/6)

Mn -0.328708 0.766211 -0.882586

O -2.845000 -1.147925 -1.653823

O -1.729927 0.623241 -2.480759

O -0.571945 -0.712441 0.348683

N 0.456157 2.544293 -2.066485

N 2.018178 0.150747 -1.794889

N 1.309620 1.678893 0.427662

N -1.237406 2.929048 0.107104

C -1.538633 3.607020 -1.169913

C -0.355749 3.604385 -2.129142

C -0.095307 4.647658 -3.024909

C 1.029085 4.548587 -3.850389

C 1.891540 3.457918 -3.720018

C 1.581258 2.467826 -2.777112

C 3.359623 -1.490801 -3.194971

C 1.116111 -0.639305 -3.961974

C 1.265873 -2.227340 -2.031925

C 1.954773 -1.040279 -2.734860

C 2.575355 1.364022 -2.417800

C 2.611372 -0.153641 -0.476457

C 2.434228 0.966902 0.533652

C 3.366930 1.255569 1.536659

C 3.082117 2.294751 2.427859

C 1.915661 3.047021 2.269089

C 1.047789 2.717247 1.219487

C -0.141113 3.594217 0.841315

C -3.142172 3.933054 1.442466

C -1.972867 1.811901 2.197621

C -2.446744 2.633086 0.982790

C -3.467844 1.765518 0.226726

C -3.241007 0.580032 -4.839047

C -4.013593 0.536955 -5.998725

C -5.000296 -0.445461 -6.108802

C -5.231425 -1.379709 -5.095698

C -4.449455 -1.321827 -3.943807

C -3.450752 -0.345349 -3.804828

C -2.617431 -0.309635 -2.529395

H -2.353221 3.057278 -1.662380

H -1.877657 4.652994 -1.034339

H -0.753382 5.518168 -3.072118

H 1.246746 5.335963 -4.576616

H 2.798723 3.385058 -4.324735

H 3.887222 -0.722042 -3.781812

H 3.999843 -1.773377 -2.344568

H 3.267467 -2.378121 -3.839894

H 0.112277 -0.303062 -3.664618

H 1.587314 0.163600 -4.549935

H 1.008632 -1.505252 -4.632338

H 1.945159 -2.744176 -1.336449

H 0.383167 -1.910268 -1.456287

H 0.957356 -2.965970 -2.786526

H 3.286694 1.831896 -1.714659

H 3.177011 1.130178 -3.309796

H 2.074979 -1.022949 -0.067849

H 3.687160 -0.412372 -0.535755

H 4.292012 0.680800 1.619069

H 3.779949 2.530023 3.235565

H 1.691659 3.882782 2.936311

H 0.273599 4.396284 0.206202

H -0.505018 4.109837 1.743282

H -2.471156 4.600122 2.006764

H -3.554255 4.501683 0.593541

H -3.983984 3.688207 2.107642

H -1.477821 0.884648 1.861571

H -1.290648 2.372534 2.854836

H -2.846711 1.535916 2.807367

H -3.884223 2.260340 -0.662937

H -3.043795 0.796888 -0.070981

H -4.314326 1.559205 0.899020

H -2.459195 1.330178 -4.712599

H -3.871551 1.242325 -6.817041

N -5.823317 -0.497164 -7.333766

H -6.012839 -2.127582 -5.228116

H -4.591502 -2.028229 -3.124082

H -1.355453 -1.207095 0.058891

O -5.601041 0.332713 -8.202852

O -6.678550 -1.365920 -7.407452

Mn^II^–OH_2_ (g) for **4** (1/6)

Mn 0.004224 1.162745 -1.095187

O -2.598906 -0.972554 -0.694262

O -1.717946 0.545751 -2.083070

O -0.516110 -0.231261 0.461633

N 0.640532 2.588598 -2.640863

N 2.002379 0.105501 -2.180564

N 1.817118 1.969565 -0.119423

N -0.631668 3.451077 -0.322723

C -1.062907 4.000397 -1.635088

C -0.064917 3.722673 -2.748874

C 0.146103 4.588672 -3.825292

C 1.107154 4.239155 -4.779926

C 1.872469 3.083390 -4.607266

C 1.625733 2.278306 -3.488385

C 2.989898 -1.877987 -3.442206

C 0.730236 -0.934564 -4.047823

C 1.033014 -2.204047 -1.921731

C 1.707259 -1.218817 -2.893333

C 2.553231 1.132243 -3.102578

C 2.859170 -0.051296 -0.978751

C 2.923083 1.211098 -0.132642

C 4.062401 1.602995 0.575352

C 4.019789 2.804304 1.291071

C 2.883165 3.612775 1.223049

C 1.789714 3.170780 0.467478

C 0.624789 4.095222 0.134882

C -2.223057 4.942695 0.986731

C -1.170552 2.912036 2.052559

C -1.730909 3.500780 0.744622

C -2.927099 2.629237 0.326449

C -3.793452 0.165721 -3.941787

C -4.839623 -0.049473 -4.837096

C -5.820883 -0.983480 -4.503182

C -5.788700 -1.705234 -3.309410

C -4.736751 -1.480479 -2.423070

C -3.734820 -0.546215 -2.732842

C -2.610405 -0.317399 -1.761300

H -0.303794 -0.549943 1.347009

H -2.017105 3.525755 -1.905999

H -1.246661 5.088998 -1.594215

H -0.422500 5.516591 -3.914483

H 1.279686 4.885382 -5.643951

H 2.660263 2.823135 -5.317606

H 3.510123 -1.256826 -4.187291

H 3.704892 -2.128541 -2.643573

H 2.727523 -2.820915 -3.944321

H -0.197567 -0.473094 -3.680207

H 1.162576 -0.284019 -4.822295

H 0.466108 -1.881129 -4.541493

H 1.727607 -2.596693 -1.164043

H 0.173584 -1.758975 -1.403813

H 0.668578 -3.071739 -2.490228

H 3.423121 1.611726 -2.621586

H 2.957317 0.676881 -4.017402

H 2.436906 -0.859367 -0.364046

H 3.891185 -0.354990 -1.232066

H 4.965144 0.988825 0.561908

H 4.887198 3.126449 1.872236

H 2.855129 4.581617 1.726614

H 1.002397 4.748139 -0.670572

H 0.449697 4.773544 0.981820

H -1.410243 5.632570 1.262431

H -2.741559 5.357148 0.109013

H -2.944010 4.950417 1.817401

H -0.795804 1.891649 1.889942

H -0.368819 3.519238 2.496657

H -1.977427 2.857636 2.797700

H -3.398919 2.956022 -0.610791

H -2.649297 1.572043 0.231306

H -3.699872 2.695350 1.106336

H -3.008402 0.887523 -4.170192

H -4.914023 0.485636 -5.783348

N -6.937450 -1.216129 -5.452772

H -6.580560 -2.423275 -3.097495

H -4.675538 -2.023807 -1.479152

H -1.412111 -0.640179 0.125127

O -6.936425 -0.567840 -6.485318

O -7.778537 -2.038387 -5.135904
